# Immune checkpoint inhibitors in gastrointestinal malignancies: an Umbrella review

**DOI:** 10.1186/s12935-023-03183-3

**Published:** 2024-01-05

**Authors:** Maryam Noori, Farideh Jafari-Raddani, Zeinab Davoodi-Moghaddam, Mahda Delshad, Saeid Safiri, Davood Bashash

**Affiliations:** 1 Student Research Committee, School of Medicine, Iran University of Medical Sciences, Tehran, Iran; 2 Department of Hematology and Blood Banking, School of Allied Medical Sciences, Shahid Beheshti University of Medical Sciences, Tehran, Iran; 3 Department of Laboratory Sciences, School of Allied Medical Sciences, Zanjan University of Medical Sciences, Zanjan, Iran; 4 Department of Community Medicine, Faculty of Medicine, Social Determinants of Health Research Center, Tabriz University of Medical Sciences, Tabriz, Iran; 5 Hematology and Oncology Research Center, Tabriz University of Medical Sciences, Tabriz, Iran

**Keywords:** Immune checkpoint inhibitor, Gastrointestinal malignancies, Microsatellite instability, Programmed death-1, PD-1, PD-L1

## Abstract

In the Modern era, immune checkpoint inhibitors (ICIs) have been the cornerstone of success in the treatment of several malignancies. Despite remarkable therapeutic advances, complex matrix together with significant molecular and immunological differences have led to conflicting outcomes of ICI therapy in gastrointestinal (GI) cancers. As far we are aware, to date, there has been no study to confirm the robustness of existing data, and this study is the first umbrella review to provide a more comprehensive picture about ICIs’ efficacy and safety in GI malignancies. Systematic search on PubMed, Scopus, Web of Science, EMBASE, and Cochrane library identified 14 meta-analyses. The pooled analysis revealed that ICIs application, especially programmed death-1 (PD-1) inhibitors such as Camrelizumab and Sintilimab, could partially improve response rates in patients with GI cancers compared to conventional therapies. However, different GI cancer types did not experience the same efficacy; it seems that hepatocellular carcinoma (HCC) and esophageal cancer (EC) patients are likely better candidates for ICI therapy than GC and CRC patients. Furthermore, application of ICIs in a combined-modal strategy are perceived opportunity in GI cancers. We also assessed the correlation of PD-L1 expression as well as microsatellite status with the extent of the response to ICIs; overall, high expression of PD-L1 in GI cancers is associated with better response to ICIs, however, additional studies are required to precisely elaborate ICI responses with respect to microsatellite status in different GI tumors. Despite encouraging ICI efficacy in some GI cancers, a greater number of serious and fatal adverse events have been observed; further highlighting the fact that ICI therapy in GI cancers is not without cost, and further studies are required to utmost optimization of this approach in GI cancers.

**Supplementary Information:**

The online version contains supplementary material available at 10.1186/s12935-023-03183-3.

## Introduction

Cancers of the gastrointestinal (GI) tract are the major leading causes of cancer-related death. Colorectal, pancreatic, liver, and esophageal cancers were the third, fourth, fifth, and seventh cause of cancer-related death in the USA in 2018, respectively [1]. Medical treatment for GI cancer can vary significantly depending on the type of cancer as well as the patient’s characteristics, such as age [2]. In some cases, neoadjuvant therapy is used to shrink the cancer before an operation. This approach has proven to be highly effective, making surgery an option for patients with otherwise inoperable tumors. Additionally, it can make the operation safer and more effective, increasing the patient's chances of a successful outcome [3, 4]. However, most patients are diagnosed in the advanced stages, so the opportunity for an extreme cure is lost [5]. Due to their prevalence as well as the limited number of treatment options, there is an urgent need to identify innovative evidence-based treatments for these insidious cancers.

The use of immunotherapy, particularly checkpoint-directed treatment, has revolutionized the treatment of oncological diseases [6]. Despite being antigenic and evoking immune responses, cancer can escape destruction through a variety of mechanisms including upregulation of immune checkpoints such as programmed death-1 (PD-1) and cytotoxic T-lymphocyte-associated antigen 4 (CTLA-4) [7]. Since Ipilimumab as the first immune checkpoint inhibitor (ICI) was approved by the Food and Drug Administration (FDA) for the treatment of melanoma patients in 2011 [8], ICIs have been investigated in a variety of cancers to the extent that now many anti-PD-1, anti-programmed-death ligand 1 (PD-L1), and anti-CTLA-4 medications like Nivolumab, Pembrolizumab, and Atezolizumab achieved FDA approval for non-small-cell lung cancer (NSCLC) [9–11], renal cell carcinoma [12, 13], head and neck squamous cell carcinoma [14, 15], and urothelial cancer [16].

In the field of GI cancers, many clinical trials assessing the safety and efficacy of different regimens such as ICI monotherapy, the combination of anti-PD-1/PD-L1 and anti-CTLA-4, or the combination of ICI with other treatment options like conventional chemotherapy, mitogen-activated protein kinase (MEK) inhibitors, and tyrosine kinase inhibitor (TKI) have been conducted; however, in many cases, there are conflicting results. While several randomized controlled trials (RCTs) indicated that ICIs could significantly improve survival of GI cancer patients [17–21], other studies failed to report a differential outcome [22–26].

As far we are aware, to date, there has been no study to confirm the robustness of existing data, and this study is the first umbrella review to provide a more comprehensive picture to address the questions of how effective are ICIs in various GI cancers? Which GI cancer is most likely to benefit from ICI therapy? Which type of ICI has the best performance? Can predictive biomarkers like PD-L1 expression and microsatellite status help to select patients who benefit the most from ICI therapy? In terms of adverse events (AEs), how does ICIs work compare with conventional therapy? And on which types of GI cancer or drug should future studies be focused?

## Material and methods

Present umbrella review was conducted in accordance with the same approach as in the guidance outlined in the *Cochrane Handbook for Systematic Reviews of Interventions* on overviews of systematic reviews [27].

### Systematic search

Two independent review authors searched PubMed, Scopus, Web of Science, EMBASE, and Cochrane library databases from inception to September 1st, 2022 to find published systematic reviews of RCTs evaluating the efficacy and safety of ICIs in patients presented with GI cancers. The following terms were used for the systematic search: (“gastrointestinal tumors” OR “esophageal cancer” OR “gastric cancer” OR “colorectal cancer” OR “pancreatic cancer” OR “hepatocellular carcinoma” OR “biliary tract cancer”) AND (“immune checkpoint inhibitor” OR “anti-CTLA-4” OR “anti-PD-1” OR “anti-PD-L1”) AND (“systematic review” OR “meta-analysis”). The complete search strategy is represented in Additional file 1: Table S1. As a hot topic, the literatures on immunotherapeutic area are updating rapidly and the RCTs are publishing frequently. Therefore, we conducted a manual search on Google Scholar search engine to cover the time gap between the latest database screening of the systematic reviews to the date we conducted database screening.

### Outcomes

Our primary outcome was efficacy of ICIs for patients with GI malignancies. The efficacy was reported as overall survival (OS), progression-free survival (PFS), objective response rate (ORR), disease control rate (DCR), complete response (CR), partial response (PR), stable disease (SD), and progressive disease (PD). In addition, the safety analysis, as secondary outcome, was comprised of the following variables; treatment-related adverse events (TRAEs), ≥ grade 3 TRAEs, grade 5 AEs, serious AEs, AEs led to treatment discontinuation, and AEs led to death. The definition of efficacy and safety outcomes are summarized in Additional file 1: Table S2.

### Study selection

Eligible meta-analyses were found through the following criteria: (1) included RCTs that were performed on adult patients with GI cancer aged 18 years or older; (2) investigated the effect of ICI therapy as compared with a control group not being any of ICIs; (3) considered either efficacy or safety outcomes; and (4) reported the effect sizes as hazard ratio (HR), risk ratio (RR), or odds ratio (OR) together with their 95% confidence intervals (CIs). We excluded any type of study other than meta-analysis, studies with no effect size (e.g. scoping reviews, narrative reviews, and systematic reviews without meta-analysis), and meta-analyses targeted patients with mixed type of cancers. Whenever multiple meta-analyses were found for an outcome, the one which was the most up-to-dated and had the highest number of primary RCTs was selected. Also, the reference list of all screened meta-analyses with the same outcome was searched to identify any potential RCTs that were not included in the selected meta-analyses. Also, further RCTs that were published in the time gap were retrieved manually by searching the Google scholar. We added these RCTs to the results of the selected meta-analyses in our umbrella review. In summary, we chose one meta-analysis for each outcome in a group of patients with the same malignancy, screened the reference list of all related meta-analyses as well as the Google scholar search engine for finding potential RCTs not included in the selected meta-analyses and included them in our study, and then performed our own meta-analysis.

### Data extraction

The following data were extracted by two authors independently from eligible meta-analyses: first author’s name, year of publication, title of systematic review, number of RCTs included in the analysis, site of tumor, efficacy and safety outcomes (i.e. OS, PFS, ORR, DCR, CR, PR, SD, PD, or AEs), type of effect sizes (i.e. HR, RR, or OR), and the variables used for subgroup analysis. We also extracted the following information from each primary RCT included in the meta-analyses and those RCTs that were found manually: first author’s name, year of publication, title of RCTs, ID of RCTs, NCT identifier, number of participants, name of medications and their target, and effect sizes for efficacy and safety outcomes.

### Assessment of methodological quality

Quality assessment was carried out independently by two reviewers and disagreement was resolved by consensus. The methodological quality of each meta-analysis was evaluated by an instrument for the assessment of multiple systematic reviews (AMSTAR) 2 tool. This checklist scores from 0 to 16 according to the information provided by individual studies. The final quality of each systematic review was classified as “high”, “moderate”, “low”, and “critically low”.

### Data synthesis and analysis

After selecting eligible meta-analyses, the results of RCTs that had been missed in the biggest meta-analyses were also added. For the sake of accuracy, we reviewed the full-texts of all primary RCTs to ensure that the reported information was correct and that all outcomes of interest were included in our review. Then we conducted our own meta-analysis. In this case, we re-calculated the HRs for OS and PFS outcomes and RRs for ORR, DCR, CR, PR, SD, PD, and AEs outcomes together with their 95% confidence intervals (CIs). For each meta-analysis, we examined between-study heterogeneity by calculating I^2^ statistic using the Cochrane’s Q test [28]. Whenever an evidence of obvious heterogeneity was detected (I^2^ ≥ 50%), we applied a random-effect model; otherwise a fixed-effect model was used [28]. For the overall analysis, we performed subgroup analyses according to the type of drug, the molecular target, and PD-L1 expression level according to combined positive score (CPS) or tumor proportion score (TPS). Several other subgroup analysis was performed for each cancer type. All analyses were performed using Stata software, version 17.0 (StataCorp), with statistical significance defined as p < 0.05.

## Results

**Fig. 1:**
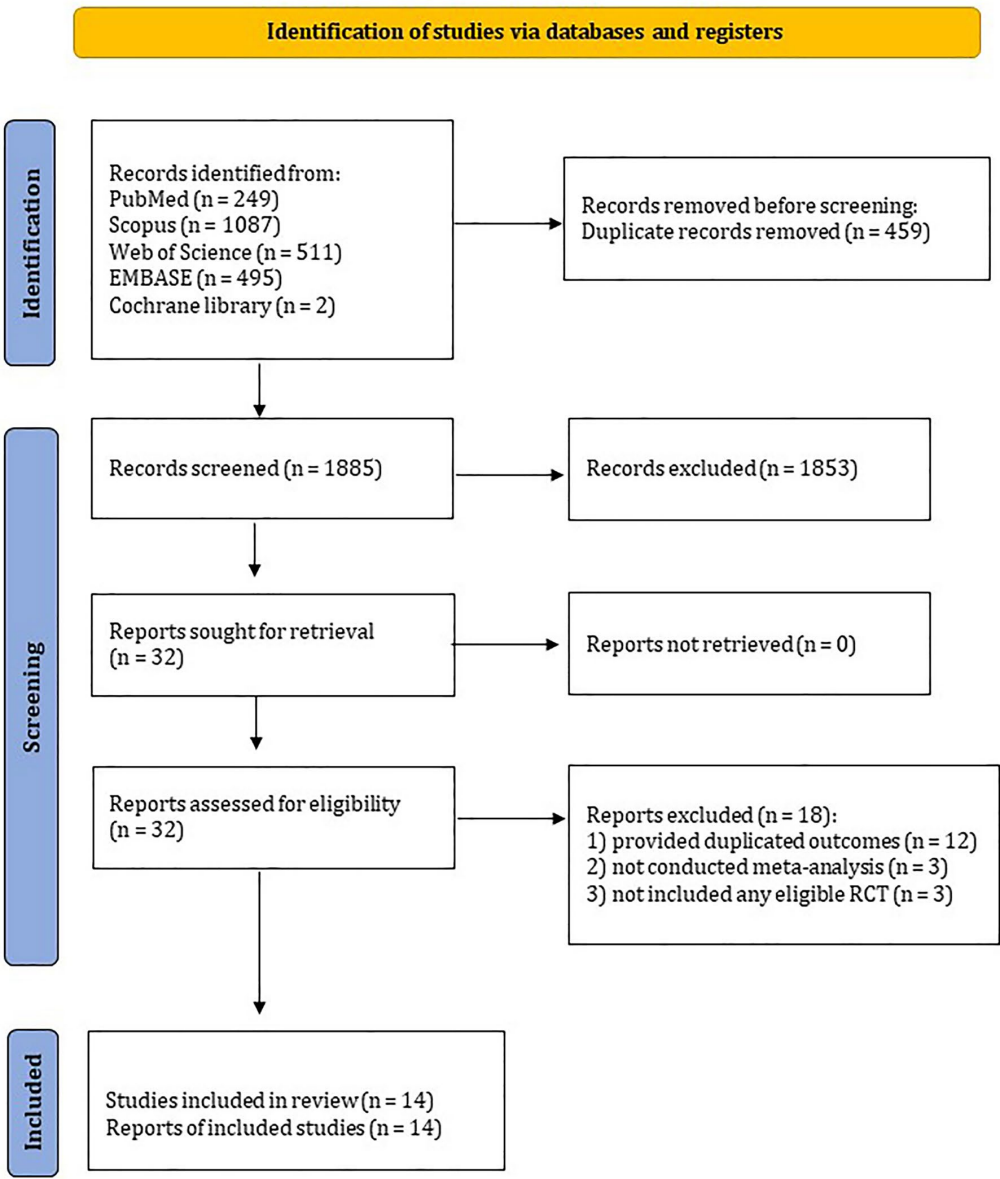
Literature search and study selection process

A total of 2344 publication were found through initial database searching. We reviewed the title and abstracts of all records and finally 32 articles were fully assessed for eligibility. Of these, 18 publications were excluded due to the following reasons: 12 records provided duplicated outcomes [29–40], three records did not conduct meta-analysis [41–43], and three records did not include eligible RCTs [42, 44, 45]. Eventually, 14 systematic reviews [46–59] containing 27 primary RCTs met the eligibility criteria and included to the present umbrella review. Furthermore, after screening the reference list of all meta-analyses and hand-searching of Google scholar engine, 15 additional primary RCTs were identified that were not included in the selected meta-analyses, resulting in a total of 42 primary RCTs to be included in the final analysis. The flow diagram of study selection is depicted in Fig. 1.

### Characteristics of the studies included in the umbrella review

The systematic search identified one eligible meta-analysis for hepatocellular carcinoma (HCC) [57], three meta-analyses for gastric cancer (GC) [47, 54, 56], three for esophageal cancer (EC) [48, 50, 59], five for both GC and EC [46, 49, 51–53], and two for colorectal cancers (CRC) [55, 58]. No meta-analysis was conducted on patients with pancreatic or biliary tract cancers. All included meta-analyses were published in 2021 and 2022. Each included meta-analysis reported unique outcomes for either main or subgroup analyses. All meta-analyses reported the efficacy outcomes, while the safety outcomes were only reported in nine meta-analyses [46, 48, 51–53, 55, 57–59]. The pooled OS and PFS outcomes were calculated in all meta-analyses, except for the meta-analyses performed by Formica et al. [47] that only calculated OS outcome. The detail characteristics of included meta-analyses are provided in Additional file 1: Table S3.

Ten primary RCTs targeted patients with HCC, 12 RCTs performed on patients with GC, 10 RCTs on patients with EC, seven RCTs on patients with CRC, two RCTs on patients with pancreatic cancer (PaC), and one RCT on patients with biliary tract cancer (BTC). A total of 23808 patients were included throughout primary RCTs. Pembrolizumab was administrated in 11 study arms, Nivolumab in seven arms, Atezolizumab in five arms, Sintilimab in four arms, Camrelizumab in three arms, Avelumab in three arms, Durvalumab in three arms, Durvalumab plus Tremelimumab in three arms, Nivolumab plus Ipilimumab in two arms, Tislelizumab in two arms, Ipilimumab and Toripalimab in one arm each. Furthermore, 28 trial arms used a medication that target PD-1, 11 arms targeted PD-L1, three arms targeted PD-L1 plus CTLA-4, two arms targeted PD-1 plus CTLA-4, and one arm targeted CTLA-4. The control group drugs were predefined routine regimens or best supportive care in all RCTs.

### Methodological quality

The detailed responses to each AMSTAR item for every meta-analysis were presented in Additional file 1: Table S4. All meta-analyses had a total score of ≥ 6 with a mean score of 7.8 points. The methodological quality of all 14 included meta-analyses was critically low. The major justifications for low quality scores were due to the fact that meta-analyses did not report the funding source of included RCTs, did not discuss the impact of methodological quality of primary studies on the overall results, and did not provide a list of excluded studies at full-text reviewing step.

### The efficacy of ICIs in GI cancers

#### Hepatocellular carcinoma (HCC)

**Fig. 2:**
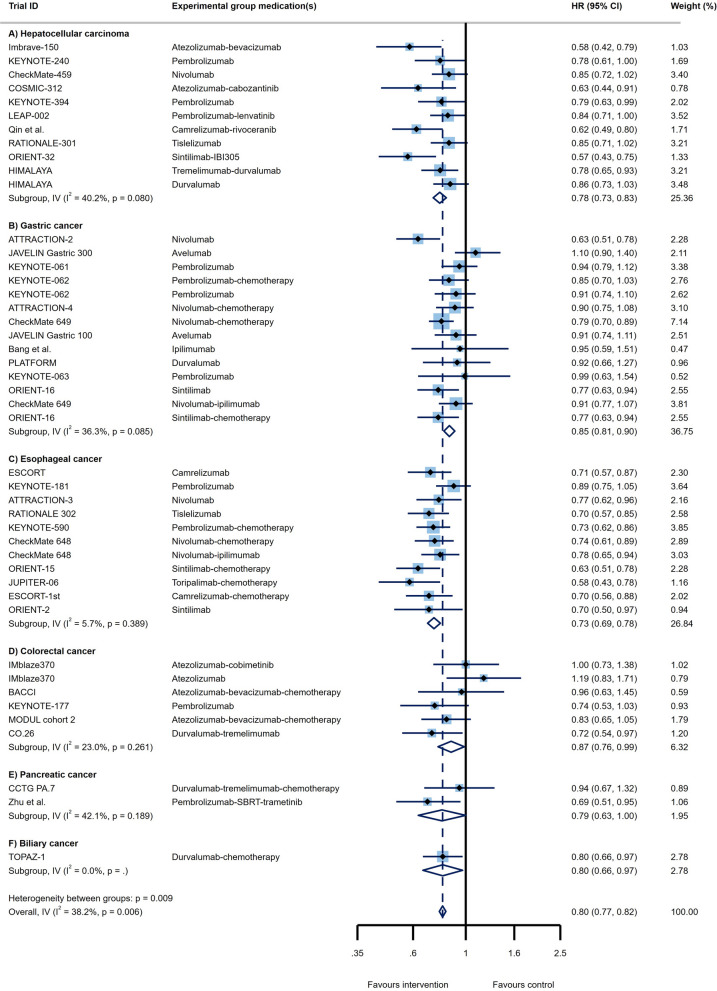
Forest plots of OS analysis in different types of GI cancers

**Fig. 3:**
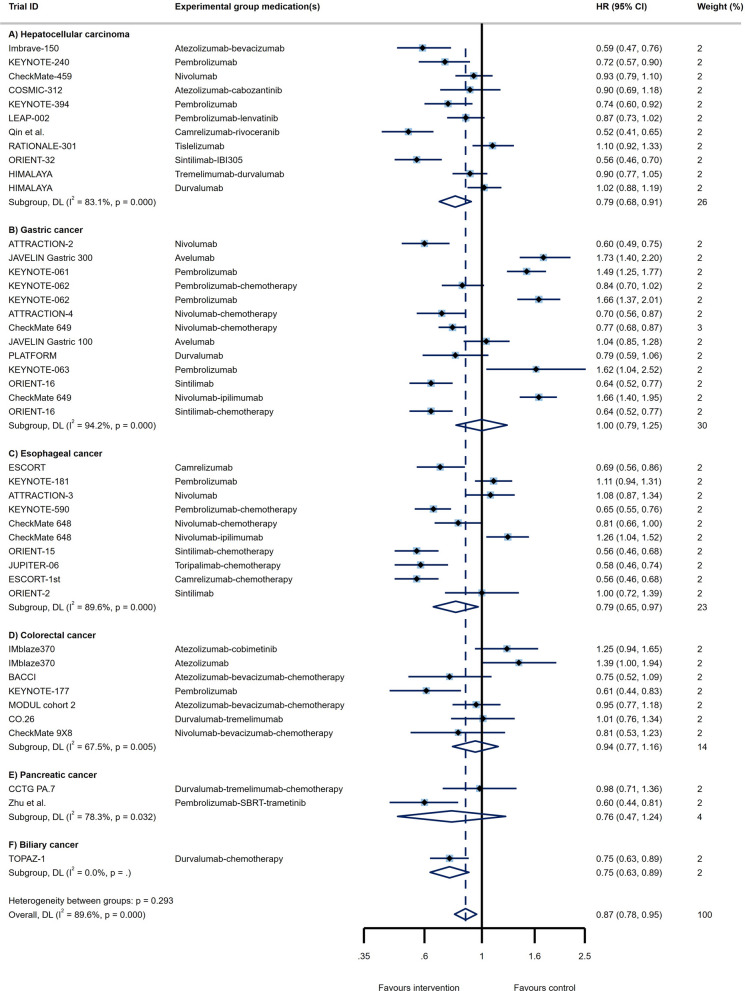
Forest plots of PFS analysis in different types of GI cancers

**Fig. 4:**
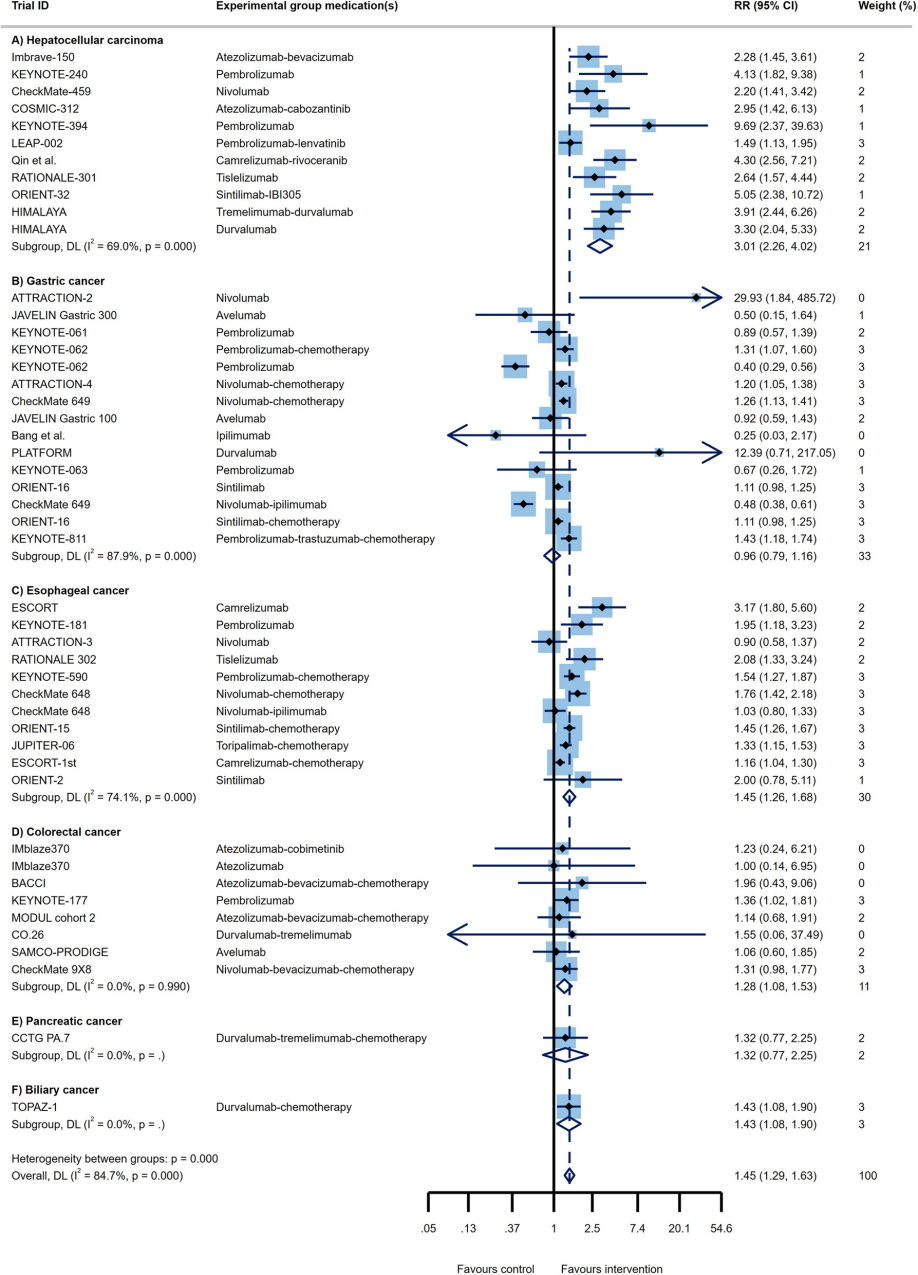
Forest plots of ORR analysis in different types of GI cancers

**Fig. 5:**
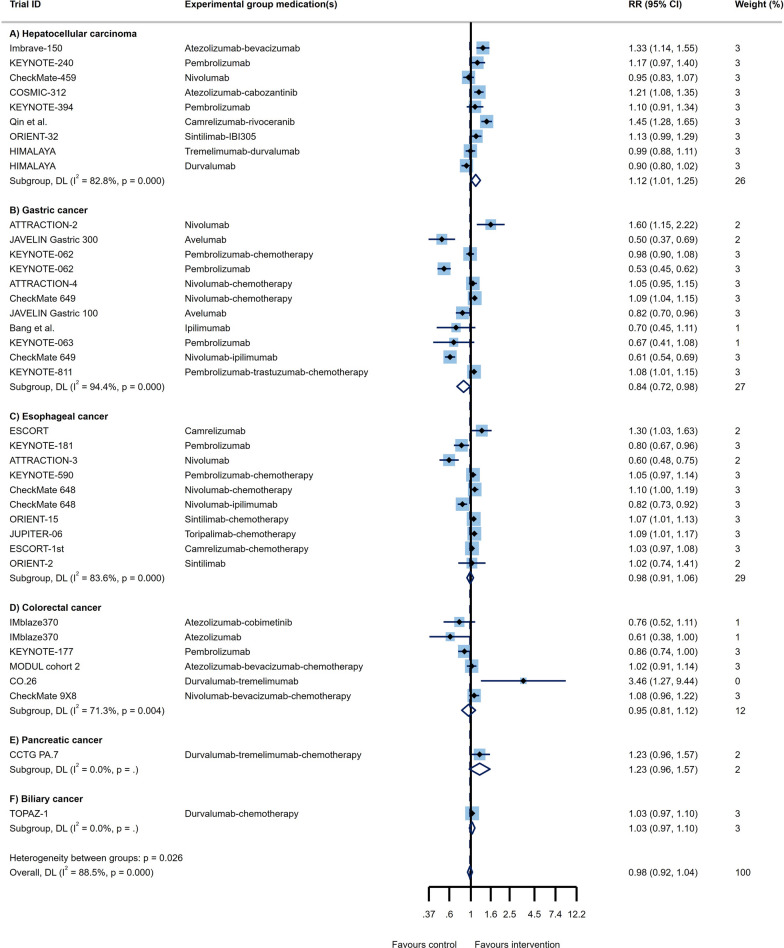
Forest plots of DCR analysis in different types of GI cancers

Through analysis of 11 RCT arms, the OS of HCC patients showed a beneficial effect of ICI therapy over conventional therapies (HR = 0.78, 95% CI 0.73 to 0.83; Fig. 2A) with moderate heterogeneity (I^2^ = 40.2%). The PFS of these patients also demonstrated improved outcomes (HR = 0.79, 95% CI 0.68 to 0.91, I^2^ = 83.1%; Fig. 3A). Impressive result of ORR (RR = 3.01, 95% CI 2.26 to 4.02, I^2^ = 69%; Fig. 4A) together with good DCR (RR = 1.12, 95% CI: 1.01 to 1.25; Fig. 5A) may provide sufficient data to suggest ICI therapy as an effective therapeutic strategy in HCC. However, it is worth noting that despite the promising results in ORR, the heterogeneity between studies was high (I^2^ = 82.8%); this issue can be justified by the differences in the types of medication and ICI targets. Differences in patient characteristics may have also accounted for the high between study heterogeneity.

Given this, we performed a subgroup analysis to investigate whether clinical variables are in charge of different outcomes in HCC cases. According to Table 1A, ICIs were able to improve the OS of all groups of patients except for those who were female, had Barcelona liver stage B, and had an etiology of hepatitis C. Tumoral macrovascular invasion (MVI) of hepatic and/or portal vein branches is a common phenomenon in HCC and is associated with poorer prognosis [60]. Several studies reported that immunotherapy with PD-1 inhibitors may be a feasible treatment option for MVI [61, 62]. In line with this finding, our OS subgroup analysis revealed that MVI and/or extrahepatic spread at study entry were associated with significant favorable survival outcomes compared to those who had not this feature (HR = 0.72 vs. HR = 0.91; p _interaction_ = 0.017). According to the subgroup analysis of PFS outcome, all groups of patients took advantage of ICI therapy except for those aged < 65 years. Both groups of patients with low (< 400 ng/ml) and high (≥ 400 ng/ml) levels of alpha-fetoprotein (AFP) have improved PFS in response to ICIs; however, lower level of AFP was associated with significant better outcome (HR = 0.46 vs. HR = 0.64; p _interaction_ = 0.021). Notably, our results indicated patients with HCC associated with viral hepatitis B, C, or a non-viral etiology had significant different OS and PFS in subgroup analysis (p _interaction_ = 0.001 and p _interaction_ = 0.028, respectively).

**Table 1:** The results of subgroups analysis based on clinical variables in different GI cancers

	OS	PFS		OS	PFS		OS	PFS
	NO	HR (95%CI)		NO	HR (95%CI)		NO	HR (95%CI)
A) Hepatocellular carcinoma								
Age				0.783				0.145
≥ 65 yr	4	0.80 (0.71, 0.91)	0.0% (0.454)		2	0.65 (0.52, 0.82)	0.0% (0.795)	
< 65 yr	4	0.82 (0.72, 0.94)	0.0% (0.892)		1	0.89 (0.63, 1.26)	NA	
Sex				0.647				0.629
Male	4	0.77 (0.69, 0.86)	0.0% (0.457)		2	0.66 (0.55, 0.80)	30.3% (0.231)	
Female	4	0.82 (0.64, 1.07)	0.0% (0.442)		2	0.60 (0.40, 0.89)	0.0% (0.968)	
ECOG performance status				0.689				0.614
0	5	0.77 (0.68, 0.88)	0.0% (0.474)		3	0.60 (0.50, 0.72)	0.0%, (0.592)	
1	5	0.74 (0.65, 0.86)	44.6% (0.124)		3	0.64 (0.53, 0.78)	24.3%, (0.267)	
Location				0.078				0.171
Asia	7	0.75 (0.67, 0.84)	0.0% (0.534)		4	0.63 (0.54, 0.75)	44.9% (0.142)	
Rest of the world	6	0.86 (0.78, 0.96)	0.0% (0.557)		3	0.74 (0.63, 0.87)	0.0% (0.872)	
AFP at baseline				0.368				0.021
≥ 400 ng/mL	5	0.66 (0.57, 0.77)	0.0% (0.942)		2	0.64 (0.53, 0.78)	35.5% (0.213)	
< 400 ng/mL	5	0.75 (0.60, 0.93)	59.8% (0.041)		2	0.46 (0.37, 0.56)	0.0%, (0.541)	
Barcelona liver stage				0.332				0.843
B	6	0.89 (0.63, 1.24)	50.7% (0.071)		3	0.63 (0.45, 0.89)	48.0% (0.146)	
C	6	0.74 (0.66, 0.84)	34.5%, (0.177)		3	0.61 (0.53, 0.70)	0.0% (0.682)	
Macrovascular invasion at study entry				0.365				0.598
Yes	5	0.71 (0.60, 0.84)	0.0%, (0.425)		3	0.58 (0.46, 0.74)	0.0% (0.546)	
No	5	0.78 (0.70, 0.87)	29.0% (0.228)		3	0.63 (0.54, 0.73)	6.4% (0.344)	
Extrahepatic spread at study entry				0.06				0.213
Yes	5	0.70 (0.62, 0.78)	49.7% (0.093)		3	0.58 (0.50, 0.67)	0.0% (0.440)	
No	5	0.84 (0.72, 0.98)	0.0%, (0.811)		3	0.70 (0.54, 0.89)	0.0% (0.368)	
MVI and/or extrahepatic spread at study entry				0.017				0.103
Yes	6	0.72 (0.66, 0.80)	15.8%, (0.312)		3	0.55 (0.47, 0.65)	0.0% (0.932)	
No	6	0.91 (0.78, 1.08)	46.4%, (0.097)		3	0.73 (0.54, 0.98)	15.7% (0.305)	
Etiology				0.001				0.028
Hepatitis B	7	0.65 (0.57, 0.74)	0.0%, (0.514)		4	0.54 (0.46, 0.64)	0.0%, (0.508)	
Hepatitis C	6	0.92 (0.77, 1.09)	42.1%, (0.125)		3	0.60 (0.43, 0.84)	0.0%, (0.633)	
Nonviral	6	0.87 (0.77, 0.98)	0.0%, (0.502)		3	0.78 (0.63, 0.96)	0.0%, (0.658)	
B) Gastric cancer								
Age				0.844				0.987
≥ 65 yr	9	0.84 (0.77, 0.93)	39.3%, (0.106)		2	1.06 (0.64, 1.74)	75.3%, (0.044)	
< 65 yr	9	0.85 (0.79, 0.93)	13.0%, (0.326)		2	1.07 (0.32, 3.52)	96.5%, (0.000)	
Sex				0.046				0.688
Male	9	0.82 (0.76, 0.88)	33.9%, (0.147)		2	1.03 (0.48, 2.18)	93.6%, (0.000)	
Female	9	0.94 (0.84, 1.06)	0.0%, (0.517)		2	1.40 (0.38, 5.18)	94.3%, (0.000)	
ECOG performance status				621				0.700
0	8	0.89 (0.77, 1.03)	43.0%, (0.092)		2	1.29 (0.46, 3.67)	93.4%, (0.000)	
1	8	0.84 (0.74, 0.97)	54.4%, (0.032)		2	0.99 (0.41, 2.39)	94.1%, (0.000)	
Location				0.884				0.372
Asia	8	0.85 (0.75, 0.97)	32.1%, (0.171)		2	1.91 (1.38, 2.64)	12.3%, (0.286)	
Rest of the world	7	0.86 (0.80, 0.93)	0.0%, (0.732)		1	1.57 (1.19, 2.08)	NA	
Lauren histological type				0.449				0.09
Diffuse type	6	0.85 (0.76, 0.96)	43.7%, (0.114)		1	0.84 (0.62, 1.14)	NA	
Intestinal type	6	0.80 (0.71, 0.90)	12.9%, (0.332)		1	0.56 (0.39, 0.80)	NA	
Primary sites				0.986				0.666
Gastric cancer	9	0.87 (0.78, 0.97)	51.1%, (0.037)		2	1.16 (0.44, 3.09)	95.9%, (0.000)	
Gastroesophageal junction cancer	9	0.87 (0.76, 0.99)	0.0%, (0.641)		2	0.89 (0.44, 1.81)	65.8%, (0.087)	
Liver metastasis				0.344				0.204
Yes	3	0.72 (0.63, 0.84)	0.0%, (0.899)		1	0.59 (0.42, 0.83)	NA	
No	3	0.82 (0.66, 1.03)	72.2%, (0.027)		1	0.79 (0.59, 1.05)	NA	
Lymph node metastasis				0.405				0.559
Yes	1	0.94 (0.77, 1.15)	NA		1	0.73 (0.58, 0.92)	NA	
No	1	0.77 (0.50, 1.18)	NA		1	0.61 (0.35, 1.06)	NA	
Peritoneal metastasis				0.130				0.002
Yes	2	0.98 (0.61, 1.56)	72.1%, (0.058)		1	1.04 (0.76, 1.43)	NA	
No	2	0.66 (0.55, 0.79)	0.0%, (0.687)		1	0.51 (0.37, 0.70)	NA	
Number of organs with metastases				0.245				0.014
< 2	4	0.72 (0.60, 0.85)	0.0%, (0.672)		1	0.42 (0.26, 0.69)	NA	
≥ 2	4	0.84 (0.68, 1.04)	78.4%, (0.003)		1	0.84 (0.66, 1.07)	NA	
Microsatellite status				0.000				
Stable	5	0.87 (0.79, 0.95)	42.1%, (0.178)					
Unstable (Instability-high)	3	0.33 (0.20, 0.52)	0.0%, (0.988)					
Prior gastrectomy				0.924				0.956
Yes	4	0.89 (0.73, 1.08)	0.0%, (0.913)		1	0.71 (0.46, 1.10)	NA	
No	4	0.90 (0.80, 1.00)	0.0%, (0.826)		1	0.70 (0.54, 0.90)	NA	
Disease status				0.313				
Metastatic	2	0.91 (0.77, 1.07)	0.0%, (0.633)					
Locally advanced	1	2.84 (0.31,25.74)	NA					
C) Esophageal cancer								
Age				0.175				0.133
≥ 65 yr	9	0.67 (0.60, 0.75)	0.0% (0.701)		6	0.58 (0.51, 0.66)	37.9%, (0.154)	
< 65 yr	9	0.74 (0.68, 0.81)	0.0%,(0.623)		6	0.66 (0.60, 0.73)	44.3%, (0.110)	
Sex				0.029				0.588
Male	10	0.72 (0.67, 0.77)	26.6%, (0.199)		6	0.63 (0.54, 0.75)	67.2%, (0.009)	
Female	10	0.90 (0.75, 1.08)	9.1%, (0.359)		6	0.69 (0.54, 0.89)	0.0%, (0.655)	
ECOG performance status				0.623				0.674
0	10	0.74 (0.66, 0.83)	0.0%, (0.449)		6	0.61 (0.50, 0.76)	34.6%, (0.177)	
1	10	0.71 (0.66, 0.78)	0.0%, (0.739)		6	0.65 (0.56, 0.75)	53.5%, (0.056)	
Location				0.145				0.889
Asia	4	0.71 (0.62, 0.80)	18.8% (0.296)		2	0.68 (0.51, 0.91)	69.6%, (0.070)	
Rest of the world	4	0.84 (0.69, 1.02)	54.6%, (0.086)		1	0.70 (0.56, 0.88)	NA	
Histology				0.240				0.239
Adenocarcinoma	2	0.92 (0.61, 1.38)	73.3%, (0.053)		1	0.63 (0.46, 0.87)	NA	
Squamous cell carcinoma	11	0.72 (0.67, 0.77)	0.0%, (0.860)		10	0.79 (0.65, 0.95)	84.9%, (0.000)	
Liver metastasis				0.358				0.451
Yes	4	0.64 (0.51, 0.80)	0.0%, (0.792)		3	0.75 (0.48, 1.15)	67.0%, (0.048)	
No	4	0.72 (0.63, 0.83)	0.0%, (0.738)		3	0.62 (0.48, 0.79)	61.8%, (0.073)	
Lymph node metastasis				0.319				
Yes	2	0.80 (0.65, 0.98)	0.0%, (0.540)					
No	2	0.68 (0.55, 0.86)	0.0%, (0.702)					
Number of organs with metastases				0.169				0.138
< 2	5	0.79 (0.69, 0.91)	0.0%, (0.923)		2	0.70 (0.56, 0.86)	0.0%, (0.350)	
≥ 2	5	0.70 (0.62, 0.79)	0.0%, (0.454)		2	0.56 (0.46, 0.68)	44.0%, (0.181)	
Disease status				0.063				0.408
Metastatic	6	0.66 (0.60, 0.74)	0.0%, (0.802)		4	0.58 (0.52, 0.65)	0.0%, (0.409)	
Locally advanced	6	0.80 (0.68, 0.95)	0.0%, (0.833)		4	0.65 (0.50, 0.85)	0.0%, (0.847)	
Smoking history				0.806				0.852
Never	5	0.77 (0.62, 0.95)	0.0%, (0.565)		2	0.79 (0.47, 1.32)	58.8%, (0.119)	
Current or former	5	0.75 (0.67, 0.83)	0.0%, (0.867)		2	0.73 (0.39, 1.36)	85.8%, (0.008)	
D) Colorectal cancer								
Age				0.570				0.804
≥ 65 yr	3	0.89 (0.52, 1.54)	69.1%, (0.039)		3	1.07 (0.81, 1.40)	0.0%, (0.539)	
< 65 yr	3	1.06 (0.85, 1.32)	0.1%, (0.368)		3	1.11 (0.91, 1.35)	47.8%, (0.147)	
Sex				0.963				0.080
Male	2	0.73 (0.55, 0.95)	0.0%, (0.383)		1	0.77 (0.57, 1.05)	NA	
Female	2	0.72 (0.50, 1.03)	39.0%, (0.200)		1	1.21 (0.81, 1.80)	NA	
ECOG performance status				0.926				0.806
0	4	0.91 (0.55, 1.50)	75.0%, (0.007)		3	1.24 (0.66, 2.32)	86.2%, (0.001)	
1	4	0.88 (0.71, 1.09)	13.0%, (0.327)		3	1.14 (0.90, 1.44)	0.0%, (0.623)	
Location				0.753				
Asia	1	0.65 (0.27, 1.56)	NA					
Rest of the world	1	0.76 (0.52, 1.09)	NA					
BRAF status				0.507				
Wild type	3	0.61 (0.39, 0.95)	0.0%, (0.720)					
Variant	2	0.71 (0.52, 0.96)	0.0%, (0.375)					
KRAS/NRAS status				0.569				
Wild type	2	0.61 (0.39, 0.95)	0.0%, (0.720)					
Variant	2	0.71 (0.52, 0.96)	0.0%, (0.375)					
Liver metastasis				0.044				0.751
Yes	1	1.14 (0.72, 1.81)	NA		2	0.86 (0.69, 1.09)	0.0%, (0.512)	
No	1	0.33 (0.11, 1.00)	NA		2	0.80 (0.51, 1.24)	0.0%, (0.523)	
Site of primary tomur				0.358				0.519
Right	3	0.78 (0.58, 1.06)	0.0%, (0.715)		3	1.00 (0.71, 1.39)	0.0%, (0.947)	
Left	3	0.95 (0.71, 1.28)	0.0%, (0.812)		3	1.13 (0.92, 1.40)	26.0%, (0.259)	
Microsatellite status				0.402				0.622
Stable	3	0.91 (0.64, 1.28)	68.9%, (0.040)		3	1.07 (0.71, 1.61)	77.2%, (0.012)	
Unstable (Instability-high)	1	0.74 (0.53, 1.03)	NA		2	0.84 (0.35, 2.01)	60.5%, (0.111)	
Number of organs with metastases								0.668
< 2					1	0.98 (0.68, 1.41)	NA	
≥ 2					1	0.88 (0.63, 1.22)	NA	
E) Pancreatic cancer								
Age				0.491				0.177
≥ 65 yr	2	0.92 (0.67, 1.25)	0.0%, (0.837)		1	0.73 (0.48, 1.11)	NA	
< 65 yr	2	0.72 (0.39, 1.34)	69.6%, (0.070)		1	0.48 (0.31, 0.75)	NA	
Sex				0.486				0.234
Male	2	0.72 (0.52, 0.99)	42.7%, (0.187)		1	0.52 (0.35, 0.77)	NA	
Female	2	0.85 (0.60, 1.20)	0.0%, (0.947)		1	0.76 (0.47, 1.23)	NA	
ECOG performance status				0.444				0.056
0	2	0.72 (0.32, 1.65)	75.4%, (0.044)		1	0.47 (0.31, 0.71)	NA	
1	2	1.01 (0.81, 1.26)	0.0%, (0.461)		1	0.87 (0.54, 1.41)	NA	
F) Biliary tract cancer								
Age				0.949				0.211
≥ 65 yr	1	0.79 (0.60, 1.04)	NA		1	0.84 (0.66, 1.07)	NA	
< 65 yr	1	0.80 (0.61, 1.04)	NA		1	0.68 (0.54, 0.85)	NA	
Sex				0.797				0.696
Male	1	0.78 (0.60, 1.01)	NA		1	0.73 (0.58, 0.92)	NA	
Female	1	0.82 (0.62, 1.08)	NA		1	0.78 (0.62, 0.99)	NA	
ECOG performance status				0.255				0.938
0	1	0.90 (0.68, 1.20)	NA		1	0.77 (0.61, 0.98)	NA	
1	1	0.72 (0.56, 0.93)	NA		1	0.76 (0.60, 0.96)	NA	
Location				0.290				0.127
Asia	1	0.72 (0.56, 0.93)	NA		1	0.67 (0.54, 0.84)	NA	
Rest of the world	1	0.89 (0.66, 1.20)	NA		1	0.87 (0.68, 1.12)	NA	
Disease status				0.108				0.012
Metastatic	1	0.49 (0.27, 0.90)	NA		1	0.42 (0.26, 0.68)	NA	
Locally advanced	1	0.83 (0.68, 1.02)	NA		1	0.81 (0.68, 0.97)	NA	

#### Gastric cancer (GC)

Fourteen study arms assessed the efficacy of different ICIs in treating GC patients. While the results showed improvement in OS (HR = 0.85, 95% CI: 0.81 to 0.90, I^2^ = 36.3%; Fig. 2B), overall PFS with HR = 1 suggests similar efficacy of the ICI and control treatments in these patients (95% CI 0.79 to 1.25, I^2^ = 94.2%; Fig. 3B). Besides, the response rates to ICIs did not reach the statistical significance as ORR had RR = 0.96 (95% CI 0.79 to 1.16, I^2^ = 87.9%; Fig. 4B). Furthermore, the DCR was found to be better in the control group relative to ICI therapy group (RR = 0.84, 95% CI 0.72 to 0.98, I^2^ = 94.4%; Fig. 5B). Of course, the high heterogeneity of studies should be considered in the final conclusion.

Regarding OS subgroup analysis, we found that ICIs prolonged survival of all groups of patients, except for female patients, those with ECOG performance status of 0, patients with no liver metastasis, both group of patients presented with or without lymph node metastasis, patients who had peritoneal metastasis, patients who had two or more organs with metastasis, patients who had prior gastrectomy, and patients who had metastatic or locally advanced esophageal tumors. Moreover, our results revealed that GC patients with high microsatellite instability (MSI) had a significant longer OS compared to stable microsatellite status when treated with ICIs (HR = 0.33 vs. HR = 0.87; p _interaction_ = 0.000). Besides, the favorable PFS outcomes were seen only in GC patients with intestinal histological type, liver metastasis, lymph node metastasis, and no history of gastrectomy. Since peritoneal metastasis associated with GC and involvement of more than 2 organs have poor prognosis, it is not surprising that these patients experience worse PFS outcomes relative to their counterparts (HR = 1.04 vs. HR = 0.51; p_interaction_ = 0.002 and HR = 0.84 vs. HR = 0.42; p_interaction_ = 0.014, respectively). The results of subgroup analysis are summarized in Table 1B.

#### Esophageal cancer (EC)

Although the published evidence from several randomized controlled clinical trials of immunotherapy for esophageal squamous cell carcinoma has shown promising outcome [18, 63, 64], there are controversial results about all outcomes in advance and metastatic stages of these patients. The pooled results of survival analysis among 11 RCT arms showed acceptable OS (HR = 0.73, 95% CI 0.69 to 0.78; Fig. 2C) with a heterogeneity of I^2^ = 5.7% and promising PFS outcomes (HR = 0.79, 95% CI 0.65 to 0.97, I^2^ = 89.6%; Fig. 3C). Although the ORR in ICI groups was better than in the control group (RR = 1.45, 95% CI 1.26 to 1.68, I^2^ = 74.1%; Fig. 4C), the DCR were not satisfactory with an RR close to 1 (RR = 0.98, 95% CI 0.91 to 1.06, I^2^ = 83.6%; Fig. 5C).

After OS subgroup analysis based on clinical variables, all groups of patients showed prolonged OS in favor of ICI therapy except for female patients, those living outside Asia, and those presented with adenocarcinoma of esophagus. Indeed, improvement in OS was significantly better in males than females (HR = 0.72 vs. HR = 0.9; p _interaction_ = 0.029; Table 1C), proposing that ICI therapy may be more effective in male rather than female with EC. In terms of PFS, all groups of patients showed prolonged PFS when received ICIs, except for those with liver metastasis and both groups patients who never smoke or reporting a history of smoking.

#### Colorectal cancer (CRC)

According to our results based on six trial arms, while patients with CRC had a favorable OS (HR = 0.87, 95% CI 0.76 to 0.99, I^2^ = 23.0%; Fig. 2D) and provided an acceptable ORR (RR = 1.28, 95% CI 1.08 to 1.53, I^2^ = 0.0%; Fig. 4D), the outcomes in terms of PFS as well as DCR did not show an obvious improvement. Thus, application of ICIs is unlikely helpful in patients with CRC. Moreover, the only groups of patients that were able to take advantage of ICI therapy were male patients, patients with wild type BRAF status, and both groups of patients with wild type or a variant of KRAS/NIRAS status. The results of subgroup analysis revealed that liver metastasis makes the OS outcome worse (HR = 1.14 vs. HR = 0.33; p _interaction_ = 0.044; Table 1D). In terms of PFS, we found that ICI therapy had no beneficial impact in any of the patients’ subgroups.

#### Pancreatic cancer (PaC)

Two RCTs showed a beneficial response in OS (HR = 0.79, 95% CI 0.63 to 1.00, I^2^ = 42.1%; Fig. 2E) but not for PFS (HR = 0.76, 95% CI 0.47 to 1.24, I^2^ = 78.3%; Fig. 3E), ORR (RR = 1.32, 95% CI 0.77 to 2.25, I^2^ = NA; Fig. 4E), and DCR (RR = 1.23, 95% CI 0.96 to 1.57, I^2^ = NA; Fig. 5E). However, there isn't still enough evidence to make a definitive judgment about their efficacies in PaC. According to subgroup analysis, only make patients showed prolonged OS in response to ICIs. Furthermore, those who aged < 65 years, male patients, and patients with ECOG performance status of 0 had significantly improved PFS (Table 1E).

#### Biliary tract cancer (BTC)

One RCT was conducted to determine whether PD-L1 targeting using Durvalumab works well among BTC patients or not. In this regard, satisfactory survival outcomes (OS: HR = 0.80, 95% CI 0.66 to 0.97; Fig. 2F and PFS: HR = 0.75, 95% CI 0.63 to 0.89; Fig. 3F) along with acceptable clinical responses (ORR: RR = 1.43, 95% CI 1.08 to 1.90; Fig. 4F and DCR: RR = 1.03, 95% CI 0.97 to 1.10; Fig. 5F) were obtained. Also, in the subgroup analysis, ICIs did not change the OS of patients except for those with ECOG performance status 1, living in Asia, and presented with metastatic disease. Furthermore, PFS subgroup analysis revealed that ICIs prolong the survival rate of all patients except for those who aged ≥ 65 years and not living in Asia. In this case, patients with metastatic status experience better PFS rather than the locally advanced stage of the disease (HR = 0.42 vs. HR:0.81; p _interaction_ = 0.012; Table 1F).

#### Pooled analysis in GI cancers

**Fig. 6:**
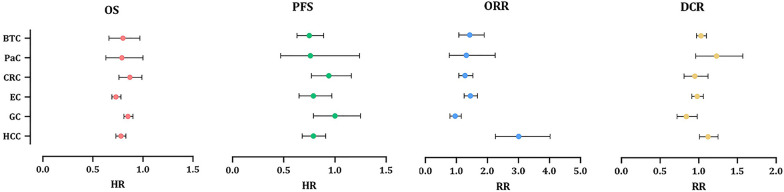
Forest plots of pooled OS, PFS, ORR, and DCR analysis in different types of GI cancers

Although ICIs are used in different GI cancers, they are not equally effective in treating all of them; while some demonstrate superior responses over conventional therapies, others don’t. Through pooled analysis, we tried to answer the question of which GI cancer is most likely to benefit from ICI therapy and which is least likely to do so. Among all GI cancers, HCC and EC seem to have the best response to ICIs not only with high DCR and ORR but also with low PFS and OS. Following HCC and EC, BTC showed promising responses according to notable ORR, DCR, PFS, and OS. Noteworthy, due to the fact that only one RCT investigated the effects of ICIs in BTC, a definitive conclusion cannot be reached. Similarly, more RCTs are needed to come to a conclusive conclusion regarding the effects of ICIs in PaC, since OS was promising, but PFS, ORR, and DCR did not meet the expectation. Finally, neither GC nor CRC had satisfactory results. Furthermore, the pooled results of CR, PR, SD, and PD for all GI cancers are represented in Additional file 1: Fig S1-S4. All in all, overall OS (HR = 0.80, 95% CI 0.77 to 0.82, p = 0.006, I^2^ = 38.2%; Fig. 2), PFS (HR = 0.87, 95% CI 0.78 to 0.95, p = 0.000, I^2^ = 89.6%; Fig. 3), and ORR (RR = 1.45, 95% CI 1.29 to 1.63, p = 0.000, I^2^ = 84.7%; Fig. 4), demonstrate that ICI therapy outperforms conventional therapies in GI cancers; however, DCR were not satisfactory with an RR close to 1 (RR = 0.98, 95% CI 0.92 to 1.04, p = 0.000, I^2^ = 88.5%; Fig. 5). Figure 6 represents pooled results of OS, PFS, ORR, and DCR in six types of GI cancers.

#### Subgroup analysis based on drug type

Since drug development paradigms for immunotherapy are evolving, comparing the effects of different drugs seems critical in order to make the best treatment decision. Notably, our results indicated patients who were treated with different drugs, had significantly different OS and PFS in subgroup analysis (p _interaction_ = 0.001 and p _interaction_ = 0.000, respectively). Among the eleven types of drugs examined and according to the results of one trial, Toripalimab had the best OS (HR = 0.58, 95% CI 0.43 to 0.78) and PFS (HR = 0.58, 95% CI 0.46 to 0.74); suggesting further investigation of this drug in clinical trials is necessary. In addition, a total of three trials conducted with Camrelizumab have reported this drug had notable OS (HR = 0.68, 95% CI 0.60 to 0.77, I^2^ = 0.0%) and PFS (HR = 0.59, 95% CI 0.50 to 0.69, I^2^ = 42.3%). Following Camrelizumab, Sintilimab also showed significant OS (HR = 0.70, 95% CI 0.63 to 0.77, I^2^ = 12.5%) and PFS (HR = 0.64, 95% CI 0.55 to 0.75, I^2^ = 60.7%). Avelumab, on the other hand, had the worst OS (HR = 0.99, 95% CI 0.85 to 1.15, I^2^ = 34.9%), indicating this agent has similar effects to conventional therapies. Moreover, combination of Nivolumab and Ipilimumab reduced the PFS of the patients compared to the control agents (HR = 1.47, 95% CI 1.30 to 1.67, I^2^ = 78.3%). The results of OS and PFS subgroup analysis based on different drug types are detailed in Table 2A.

**Table 2:** Detailed on subgroups analysis based on drug types, target, and PD-L1 cutoff

	OS					PFS				
	HR	95% CI	No. of trails	I^2^(P-value)	P _interaction_	HR	95% CI	No. of trails	I^2^(P-value)	P _interaction_
A) Drug					**0.002**					**0.000**
Pembrolizumab	0.83	(0.78, 0.89)	11	0.0%, (0.560)		0.92	(0.74, 1.14)	11	91.5%, (0.000)	
Nivolumab	0.79	(0.73, 0.84)	6	33.3%, (0.186)		0.92	(0.74, 1.14)	6	91.3%, (0.000)	
Atezolizumab	0.83	(0.73, 0.94)	6	60.8%, (0.026)		0.93	(0.72, 1.19)	6	79.9%, (0.000)	
Sintilimab	0.70	(0.63, 0.77)	5	12.5%, (0.334)		0.64	(0.55, 0.75)	5	60.7%, (0.038)	
Durvalumab	0.84	(0.75, 0.95)	3	0.0%, (0.737)		0.88	(0.79, 0.98)	3	73.3%, (0.024)	
Camrelizumab	0.68	(0.60, 0.77)	3	0.0%, (0.681)		0.59	(0.50, 0.69)	3	42.3%, (0.177)	
Durvalumab + Tremelimumab	0.79	(0.69, 0.91)	3	0.0%, (0.495)		0.93	(0.82, 1.06)	3	0.0%, (0.743)	
Tislelizumab	0.78	(0.68, 0.89)	2	50.2%, (0.156)		1.10	(0.91, 1.32)	1	NA	
Avelumab	0.99	(0.85, 1.15)	2	34.9%, (0.215)		1.34	(0.81, 2.20)	2	90.7%, (0.001)	
Nivolumab + Ipilimumab	0.85	(0.75, 0.96)	2	33.1%, (0.222)		1.47	(1.30, 1.67)	2	78.3%, (0.032)	
Ipilimumab	0.95	(0.59, 1.51)	1	NA		**-**	**-**	**-**	**-**	
Toripalimab	0.58	(0.43, 0.78)	1	NA		0.58	(0.46, 0.74)	1	NA	
B) Target					**0.058**					**0.000**
PD-1	0.78	(0.75, 0.81)	28	33.3%, (0.046)		0.82	(0.72, 0.93)	28	89.1%, (0.000)	
PD-L1	0.87	(0.81, 0.93)	11	46.5%, (0.044)		0.97	(0.83, 1.14)	11	84.1%, (0.000)	
PD-L1 + CTLA-4	0.79	(0.69, 0.91)	3	0.0%, (0.495)		0.93	(0.82, 1.06)	3	0.0%, (0.743)	
PD-1 + CTLA-4	0.85	(0.75, 0.96)	2	33.1%, (0.222)		1.47	(1.30, 1.67)	2	78.3%, (0.032)	
CTLA-4	0.95	(0.59, 1.51)	1	NA		-	-	-	-	
C) PD-L1 cutoff										
CPS = 1					**0.000**					**0.475**
CPS ≥ 1	0.76	(0.72, 0.81)	13	40.4%, (0.065)		0.91	(0.71, 1.16)	10	91.1%, (0.000)	
CPS < 1	1.03	(0.88, 1.19)	9	0.0%, (0.819)		1.08	(0.72, 1.61)	6	75.5%, (0.001)	
CPS = 5					**0.002**					**0.758**
CPS ≥ 5	0.70	(0.65, 0.76)	9	0.0%, (0.488)		0.76	(0.59, 0.97)	7	87.2%, (0.000)	
CPS < 5	0.87	(0.78, 0.97)	6	29.9%, (0.211)		0.82	(0.55, 1.21)	3	79.1%, (0.008)	
CPS = 10					**0.014**					**0.701**
CPS ≥ 10	0.69	(0.63, 0.76)	12	0.0%, (0.512)		0.70	(0.58, 0.84)	8	60.8%, (0.013)	
CPS < 10	0.82	(0.74, 0.91)	8	9.7%, (0.355)		0.75	(0.57, 0.97)	5	72.1%, (0.006)	
TPS = 1%					**0.000**					**0.341**
TPS ≥ 1%	0.66	(0.60, 0.73)	13	17.2%, (0.270)		0.73	(0.59, 0.91)	9	63.6%, (0.005)	
TPS < 1%	0.86	(0.80, 0.92)	12	32.2%, (0.133)		0.87	(0.66, 1.13)	8	86.9%, (0.000)	
TPS = 5%					**0.037**					**0.068**
TPS ≥ 5%	0.64	(0.55, 0.73)	6	0.0%, (0.902)		0.52	(0.43, 0.63)	3	0.0%, (0.844)	
TPS < 5%	0.77	(0.69, 0.85)	6	0.0%, (0.590)		0.65	(0.56, 0.75)	3	43.6%, (0.170)	
TPS = 10%					**0.036**					**0.121**
TPS ≥ 10%	0.63	(0.54, 0.73)	7	0.0%, (0.772)		0.54	(0.43, 0.66)	4	0.0%, (0.657)	
TPS < 10%	0.76	(0.69, 0.84)	7	0.0%, (0.922)		0.69	(0.54, 0.88)	4	68.6%, (0.023)	

#### Subgroup analysis based on target type

It has been reported that targeting PD-1, PD-L1 or CTLA-4 may provide different outcomes in gastric cancer [65] and hepatocellular carcinoma [66]; thus, hypothesizing whether the application of different ICIs was associated with different efficacies in patients with GI cancer. Subgroup analysis based on PD-1, PD-L1, and CTLA4 inhibitors showed non-significantly different OS but significant different PFS between these groups (p _interaction_ = 0.058 and p _interaction_ = 0.000, respectively). In this regard, PD-1 inhibitors outperformed PD-L1 inhibitors according to OS (HR = 0.78, 95% CI 0.75 to 0.81, I^2^ = 33.3% vs. HR = 0.87, 95% CI 0.81 to 0.94, I^2^ = 46.5%, respectively). In terms of PFS, only PD-1 inhibitors showed promising results (HR = 0.80, 95% CI 0.77 to 0.83, I^2^ = 89.1%); however, PD-L1 inhibitors did not change the PFS outcome (HR = 0.96, 95% CI 0.90 to 1.03, I^2^ = 84.1%). As there are limited clinical trials with CTLA4 inhibitors, a conclusion cannot be drawn so far. Table 2B represents the results of OS and PFS subgroup analysis based on target type.

#### Subgroup analysis based on PD-L1 expression

The response to ICIs varies from patient to patient, therefore, several predictive biomarkers are developed to determine sensitivity and resistance to immune checkpoint inhibitors. In this regard, PD-L1 expression on either tumor or immune cells is the most frequently studied biomarker [67].

Subgroup analysis based on the PD-L1 expression demonstrated that patients with CPS ≥ 1, ≥ 5, and ≥ 10 have better OS compared with < 1, < 5, and < 10 with p_interaction_ = 0.000, 0.002, and 0.014 respectively. The same goes for TPS, and TPS ≥ 1%, ≥ 5%, and ≥ 10% had longer OS than < 1%, < 5%, and < 10% with p_interaction_ = 0.000, 0.037, and 0.036 respectively. Moreover, those with CPS ≥ 10 and TPS ≥ 10% have the best OS compared to the control agents (HR = 0.69, 95% CI 0.63 to 0.76, I^2^ = 0.00% and HR = 0.63, 95% CI 0.54 to 0.73, I^2^ = 0.00% respectively). In terms of PFS, no difference was detected between the upper and lower limits of each threshold Table 2C shows the results of PD-L1 expression subgroup analysis.

### The safety of ICIs in GI cancers

**Fig. 7:**
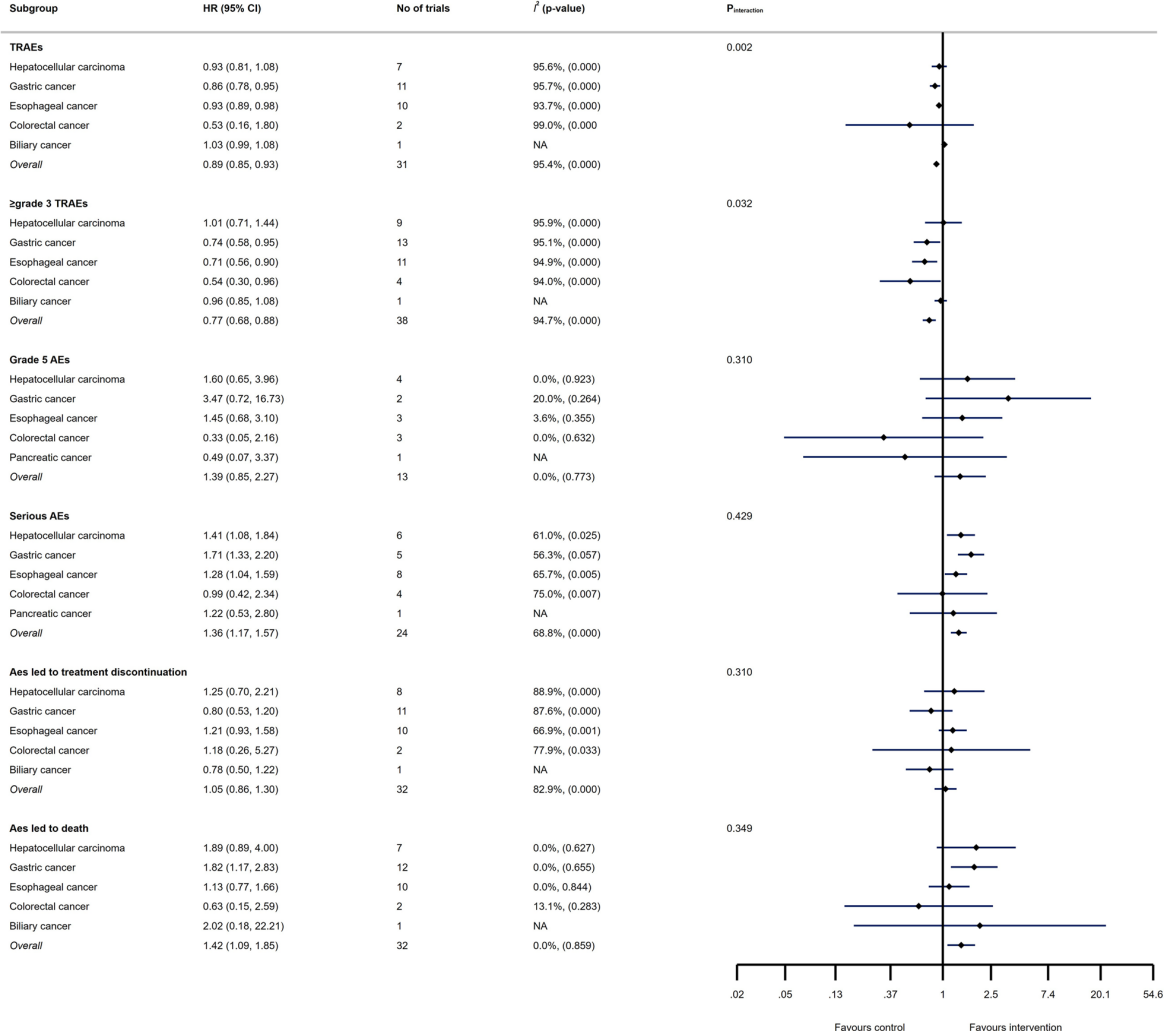
Detailed on TRAEs, ≥ grade 3 TRAEs, grade 5 TRAEs, serious AEs, AEs led to treatment discontinuation, and AEs led to death in different GI cancers following ICI therapy

Overall RR of TRAEs and ≥ grade 3 TRAEs were 0.89 (95% CI 0.85 to 0.93, I^2^ = 95.4%) and 0.77 (95% CI 0.68 to 0.88, I^2^ = 94.7%), respectively; indicating that ICI therapy may possess fewer TRAEs and ≥ grade 3 TRAEs than conventional therapies. However, serious AEs and AEs led to death were more common in patients treated with ICIs compared with conventional therapies (RR = 1.36, 95% CI 1.17 to 1.57, I^2^ = 68.8% and RR = 1.42, 95% CI 1.09 to 1.85, I^2^ = 0.0%, respectively). The results concerning the safety of ICIs in GI cancers are depicted in Fig. 7.

## Discussion

Although ICIs successfully used to treat various solid tumors such as metastatic melanoma [68], renal cell carcinoma [69], lung cancer [70], and squamous cell carcinoma of the head and neck [71], there are controversial results about ICIs’ efficacies in patients with GI tumors. As far as we are aware, this is the first umbrella review in the context of GI malignancies, reporting that application of ICIs in this group of cancers could partially improve response rates compared to conventional therapies (Figs. 2–5); however, different types of GI tumors did not experience the same efficacy. The results of the pooled analysis showed that HCC and EC patients are most likely to benefit from ICI therapy, while it is less useful in GC and CRC patients. Also, the results for PaC and BTC are at preliminary stage and need to be investigated in future.

Regarding the great potential of ICIs in HCC, patients in the advanced stages of disease and even those with MVI could benefit from this strategy. Of note, HCC patients with chronic inflammation caused by hepatitis B or C infection are more susceptible to ICI therapy; indeed, the expression of immune checkpoint molecules which is increased through the inflammatory condition may explain this phenomenon [72, 73]. Besides HCC, EC patients have promising responses to ICIs mainly targeting PD-1. Accordingly, the study conducted by Chalmers et al. reported that EC patients have a high mutation load which caused the emergence of immunogenic neoantigens, making these patients good candidates for ICI treatment [74]. On the other hand, while encouraging results of some studies led to the approval of anti-PD-1 drugs in GC patients after second lines of therapy failure [17, 75], there are controversial results about ICIs efficacies in this malignancy [24]. This issue perhaps occurs due to the high heterogeneity of the tumor and different expression of immune checkpoints among its various subtypes; while MSI-high and Epstein-Barr virus (EBV) positive subtypes associated with high levels of these molecules, aberrant p53 subtype didn’t show a significant correlation [76]. Concerning CRC, studies revealed that ICI therapy is only effective in a limited number of CRC patients who are MSI-high, resulting in FDA approval of several ICIs either as a monotherapy or in combination in this group [77–80]; however, it is worth noting that the result of our study shows survival outcomes are not significantly different between microsatellite stable and unstable groups of CRC patients. Generally, and regardless of microsatellite status, our results further highlight that application of ICIs is unlikely helpful in patients with CRC [58, 81]. Since the studies in the context of BTC and PaC are limited, it is difficult to make a definitive judgment about the efficacy of ICIs in these patients; however, it seems that BTC demonstrates more beneficial effects of ICI strategy rather than PaC. The immunosuppressive environment in the latter may be one of the main barriers to successful ICI therapy in this malignancy [82]. All in all and apart from the type of GI, it seems that application of ICIs in combined-modal strategies is a better approach for GI cancer treatment compared to ICI monotherapy. In this vein, most of the FDA-approved ICIs in GI cancers are in combination with other agents, and nowadays most of the ongoing clinical trials are focused on the efficacy of ICIs in these strategies. To provide a better overview, we summarized FDA approved ICIs and ongoing clinical trials among different GI cancers in Tables 3 and 4, respectively.

**Table 3:** The list of FDA approval of ICIs in different GI cancers

NCT	Trial name	Trial design	Standard arm	Line	Treatment			OS (Months)	PFS (Months)	PDL1 Status
					Name of drug	Type of drug	Mode			
HCC										
NCT03434379	IMbrave-150	Open label randomized phase III	Sorafenib	First	Atezolizumab + Bevacizumab	Anti-PD-L1 + Anti-VEGF	Combination	Not est. vs 13.2	6.8 vs 4.3	No
NCT02702414	Keynote-244	Open label, non-randomized phase II	None	Second	Pembrolizumab	Anti-PD1	Monotherapy	13.2	4.9	No
NCT01658878	Checkmate-040	Open label, non-comparative, dose escalation and expansion phase I/II	None	Second	Nivolumab	Anti-PD1	Monotherapy	83% at 6; 74% at 9	37% at 6; 28% at 9	No
NCT01658878	Checkmate-040	Open label, non-comparative, dose escalation and expansion Phase I/II	None	Second	Nivolumab + Ipilimumab	Anti-PD1 + Anti- CTLA4	Combination	22.8		No
GC										
NCT02872116	Checkmate-649	Open label randomized phase III	Capecitabine and Oxaliplatin	First	Nivolumab + capecitabine + oxaliplatin	Anti-PD1 + chemotherapy	Combination	13.1 vs 11.1	7·7 vs 6.05	No
NCT02335411	Keynote-059	Open label single arm phase II	None	Third	Pembrolizumab	Anti-PD1	Monotherapy	5.6	2	CPS > 1
NCT02267343	Attraction-2	Double-blind randomized phase III	Placebo	Third	Nivolumab	Anti-PD1	Monotherapy	5·26 vs 4·14	1·61 vs 1.45	No
NCT01928394	Checkmate-032	Open label phase I/II		Third	Nivolumab + Ipilimumab	Anti-PD1 + Anti- CTLA4	Combination	6.9	1.4	No
EC										
NCT03189719	Keynote-590	Double-blind randomized phase III	5-fluorouracil and Cisplatin	First	Pembrolizumab + Cisplatin + Fluorouracil	Anti-PD1 + chemotherapy	Combination	13.9 vs 8.8	7.5 vs 5.8	CPS ≥ 10
NCT03143153	Checkmate-648	Open label randomized phase III	5-fluorouracil and Cisplatin	First	Nivolumab + Ipilimumab	Anti-PD1 + Anti- CTLA4	Combination	13.7 vs. 9.1	4 vs 4.4	TPS ≥ 1%
NCT03143153	Checkmate-648	Open label randomized phase III	5-fluorouracil and Cisplatin	First	Nivolumab + Cisplatin + Fluorouracil	Anti-PD1 + chemotherapy	Combination	15.4 vs. 9.1	6.9 vs 4.4	TPS ≥ 1%
NCT02564263	Keynote-181	Open label randomized phase III	Paclitaxel, Docetaxel, and Irinotecan	Second	Pembrolizumab	Anti-PD1	Monotherapy	9.3 vs 6.7	2.6 vs 3	CPS ≥ 10
NCT02559687	Keynote-180	Open label single arm phase II	None	Second	Pembrolizumab	Anti-PD1	Monotherapy	5.8	2	CPS ≥ 10
NCT02569242	Attraction-3	Open label randomized phase III	Paclitaxel or Docetaxel	Second	Nivolumab	Anti-PD1	Monotherapy	10.9 vs 8.4	1.7 vs 3.4	No
CRC										
NCT02563002	Keynote-177	Randomized phase III	mFOLFOX6/ FOLFIRI + / − Bevacizumab or Cetuximab	First *	Pembrolizumab	Anti-PD1	Monotherapy	Not achieved vs 36.7	16.5 vs 8.2	No
NCT02060188	Checkmate-142	Open label single arm Phase II	None	First *	Nivolumab	Anti-PD1	Monotherapy	73% at 12	50.4% at 12	No
NCT02060188	Checkmate-142	Open label single arm Phase II	None	First *	Nivolumab + Ipilimumab	Anti-PD1 + Anti- CTLA4	Combination	85% at 12	71% at 12	No
NCT02460198	Keynote-164	Open label single arm Phase II	None	Second *	Pembrolizumab	Anti-PD1	Monotherapy	31.4	2.3	No

^*^ For dMMR/MSI-high

**Table 4:** Selected ongoing randomized clinical trials investigating the efficacy of ICIs in GI cancer cases

NCT number	Population under study		Therapies under comparison	Monotherapy/combination		Sample size (n)
HCC						
NCT05277675	Neoadjuvant therapy in the treatment of recurrent HCC		Tislelizumab/Sintilimab + Lenvatinib/Bevacizumab + radiofrequency ablation vs radiofrequency ablation	Combination		160
NCT04183088	First-line therapy for advanced HCC		Tislelizumab + regorafenib vs regorafenib	Combination		125
NCT04658147	-		Nivolumab + Relatlimab vs Nivolumab	Monotherapy vs Combination		20
NCT04233840	-		Ropeginterferon alfa-2b + Nivolumab vs Nivolumab vs Ropeginterferon alfa-2b	Monotherapy vs Combination		72
NCT03383458	HCC who has undergone complete resection or have achieved a complete response after local ablation, and who are at high risk of recurrence		Nivolumab vs placebo	Monotherapy		545
NCT04050462	-		Nivolumab vs Nivolumab + BMS-986253 vs Nivolumab + Cabiralizumab	Monotherapy vs Combination		23
NCT04567615	-		Nivolumab and Relatlimab vs Nivolumab	Monotherapy vs Combination		250
NCT05039736	-		Cabozantinib vs Nivolumab	Monotherapy		30
NCT04039607	Advanced HCC		Nivolumab + Ipilimumab vs Sorafenib + lenvatinib	Combination		732
NCT02576509	First- line treatment in patients with advanced HCC		Nivolumab vs Sorafenib	Monotherapy		743
NCT04340193	Intermediate-stage HCC		Nivolumab + Ipilimumab + Trans-arterial ChemoEmbolizatio vs Nivolumab + Trans-arterial ChemoEmbolizatio vs Trans-arterial ChemoEmbolizatio	Combination		26
NCT04268888	Intermediate-stage HCC		Transarterial Chemoembolisation and/or Transarterial Embolisation vs Transarterial Chemoembolisation and/or Transarterial Embolisation + Nivolumab	Combination		552
NCT05337137	untreated advanced/metastatic HCC		Relatlimab + Nivolumab + Bevacizumab vs Nivolumab + Bevacizumab + Placebo	Combination		162
NCT04777851	Intermediate-stage HCC		Regorafenib + Nivolumab vs Transarterial Chemoembolization	Combination		496
GC						
NCT04294784	Patients with advanced or recurrent GC and GEJ in second-line treatment		albumin-bound paclitaxel and SHR-1210 (PD-1 inhibitor) vs albumin-bound paclitaxel	Combination		80
NCT04997837	D2/R0 resected pn3 GC or GEJ		Nivolumab/Toripalimab + Oxaliplatin + Capecitabine + Tegafur-gimeracil-oteracil potassium + 5-FU + RT vs Oxaliplatin + Capecitabine + Tegafur-gimeracil-oteracil potassium + 5-FU + RT	Combination		433
NCT04782791			Nivolumab + oxaliplatin + Gastrectomy vs Nivolumab + Gastrectomy	Combination		30
NCT03443856	Stage Ib-iva GC and GEJ and high risk of recurrence following neoadjuvant chemotherapy and resection		Nivolumab + Ipilimumab vs chemotherapy	Combination		197
NCT02935634	Advanced GC		Nivolumab + Ipilimumab vs Nivolumab + Relatlimab vs Nivolumab + BMS-986205 vs Nivolumab + Rucaparib vs Ipilimumab + Rucaparib vs Nivolumab + Ipilimumab + Rucaparib	Combination		190
NCT02746796	Unresectable advanced or recurrent GC and GEJ as the first-line therapy		Nivolumab + Oxaliplatin + Tegafur- Gimeracil-Oteracil potassium vs Nivolumab + Oxaliplatin + Capecitabine vs Nivolumab + Oxaliplatin + Tegafur- Gimeracil-Oteracil potassium + Capecitabine vs Oxaliplatin + Tegafur- Gimeracil-Oteracil potassium + Capecitabine + Placebo	Combination		680
NCT03006705	In stage III GC and GEJ after D2 or more extensive lymph node dissection		Nivolumab + Oxaliplatin + Tegafur- Gimeracil-Oteracil potassium + Capecitabine vs Oxaliplatin + Tegafur- Gimeracil-Oteracil potassium + Capecitabine + Placebo	Combination		800
NCT05144854	Chemotherapy-naïve participants with HER2-negative unresectable advanced or recurrent GC and GEJ		Nivolumab + Ipilimumab + Oxaliplatin + Capecitabine + S-1 vs Oxaliplatin + Capecitabine + S-1	Combination		600
NCT03647969	Patients with Her2 negative, previously untreated metastatic esophagogastric adenocarcinoma		Nivolumab + Ipilimumab + mFOLFOX vs mFOLFOX vs Nivolumab + FLOT	Combination		262
NCT04062656	GC and GEJ		Nivolumab + Oxaliplatin + Docetaxel + 5-FU + Folic acid vs Nivolumab + Oxaliplatin + Docetaxel + 5-FU + Folic acid + relatlimab	Combination		21
NCT02872116	GC and GEJ		Nivolumab + Ipilimumab vs Oxaliplatin + Capecitabine vs Oxaliplatin + Leucovorin + Fluorouracil vs Nivolumab + Oxaliplatin + Capecitabine vs Oxaliplatin + Leucovorin + Fluorouracil + Nivolumab	Combination		2031
NCT04879368	Gastro-esophageal cancer		Regorafenib + Nivolumab vs Docetaxel + Paclitaxel + Irinotecan + Trifluridine/Tipracil	Combination		450
NCT05568095	locally advanced unresectable or metastatic GC, and GEJ and EC		Zimberelimab + Domvanalimab + Oxaliplatin + Leucovorin + Fluorouracil + Capecitabine vs Nivolumab Oxaliplatin + Leucovorin + Fluorouracil + Capecitabine	Combination		970
EC						
NCT05182944	-		Camrelizumab + Albumin Paclitaxel + Cisplatin + Camrelizumab vs Camrelizumab vs best supportive care	Monotherapy vs Combination		130
NCT05007145	Resectable locally advanced thoracic EC		PD-1 inhibitor + Albumin-Bound Paclitaxel + Cisplatin vs Albumin-Bound Paclitaxel + Cisplatin + Radiation	Combination		92
NCT04785820	Advanced or metastatic		RO7121661 vs RO7247669 vs Nivolumab	Monotherapy		210
NCT05213312	Esophageal squamous cell carcinoma		Nivolumab + Cisplatin + Paclitaxel + 5Fluorouracil vs Cisplatin + Paclitaxel + 5Fluorouracil	Combination		90
NCT03604991	Esophageal adenocarcinoma, Gastroesophageal junction adenocarcinoma		Carboplatin + Paclitaxel + RT vs Carboplatin + Nivolumab + Paclitaxel + RT vs Nivolumab vs Ipilimumab + Nivolumab	Monotherapy vs Combination		514
CRC						
NCT03926338	dMMR/MSI-H phenotype		Toripalimab + Celecoxib vs Toripalimab	Monotherapy vs Combination		100
NCT05141721	Metastatic CRC		GRT-C901/GRT-R902 + Ipilimumab + Fluoropyrimidine + leucovorin + Bevacizumab + Atezolizumab vs Fluoropyrimidine + leucovorin + Bevacizumab	Combination		665
NCT04907539	RNF43 or RSPO aberrated, MSS		RXC004 + Denosumab vs RXC004 + Denosumab + Nivolumab	Combination		50
NCT05308446	Metastatic or unresectable BRAF-mutant		Cetuximab + Encorafenib vs Cetuximab + Encorafenib + Nivolumab	Combination		84
NCT03388190	MSS/ pMMR phenotype		Oxaliplatin vs oxaliplatin + Nivolumab	Combination		80
NCT03414983		Metastatic CRC	Oxaliplatin + Leucovorin + Fluorouracil + Bevacizumab vs Oxaliplatin + Leucovorin + Fluorouracil + Bevacizumab + Nivolumab	Combination	195	
NCT04008030		MSI-H/dMMR phenotype	Nivolumab vs Nivolumab + Ipilimumab vs Oxaliplatin + Leucovorin + Fluorouracil + Irinotecan + Bevacizumab + Cetuximab	Monotherapy vs Combination	831	
NCT03803553		Metastatic CRC	5-Fluorouracil + Irinotecan + Leucovorin vs Nivolumab vs Encorafenib + Binimetinib + Cetuximab vs active surveillance	Monotherapy	500	
NCT03377361		-	Nivolumab + trametinib vs Nivolumab + Ipilimumab + trametinib vs Nivolumab + Ipilimumab + trametinib vs Nivolumab + Ipilimumab + trametinib vs Regorafenib vs Nivolumab + Ipilimumab + trametinib	Combination	232	
NCT05328908		Failed 1–4 prior lines of therapy	Nivolumab + Relatlimab vs Regorafenib or TAS-102	Combination	700	
PaC						
NCT04612530		-	Nivolumab vs Irreversible Electroporation (IRE) + Nivolumab vs IRE + Nivolumab + Toll-Like Receptor 9	Monotherapy vs Combination	18	
NCT04953962		Stage IV	CBP501 (25) + Cisplatin + Nivolumab vs CBP501 (25) + Cisplatin vs Cisplatin + Nivolumab	Combination	36	
NCT03563248		Localized PaC	FOLFIRINOX + SBRT + surgery vs FOLFIRINOX + SBRT + surgery + Losartan vs FOLFIRINOX + SBRT + surgery + Losartan + Nivolumab vs FOLFIRINOX + SBRT + surgery + Nivolumab	Combination	168	
NCT03336216		Advanced PaC	Nab-paclitaxel + Onivyde + Fluorouracil + Gemcitabine + Leucovorin + irinotecan hydrochloride vs Cabiralizumab + Nivolumab vs Cabiralizumab + Nivolumab + Nab-paclitaxel + Gemcitabine vs Cabiralizumab + Nivolumab + Fluorouracil + Oxaliplatin + Leucovorin	Combination	202	
NCT04229004		Metastatic PaC	Gemcitabine + Nab-paclitaxel vs Oxaliplatin + Leucovorin + Irinotecan + 5-Fluorouracil vs amrevlumab + gemcitabine + nab-paclitaxel vs Canakinumab + Spartalizumab + nab-paclitaxel + gemcitabine vs SM-88	Combination	825	
NCT02451982		Surgically resectable pancreatic ductal adenocarcinoma	Cyclophosphamide + GVAX vs Cyclophosphamide + GVAX + Nivolumab vs Cyclophosphamide + GVAX + Nivolumab + Urelumab vs Nivolumab + BMS-986253	Combination	76	
Different cancers						
NCT03184870		mCRC and mPaC	BMS-813160 + 5-fluorouracil + Leucovorin + Irinotecan vs BMS-813160 + Nab-paclitaxel + Gemcitabine vs BMS-813160 and Nivolumab	Combination	332	
NCT03373188		Stage I-III PaC, stage IV CRC	Surgery vs VX15/2503 + surgery vs VX15/2503 + Ipilimumab + surgery vs VX15/2503 + Nivolumab + surgery	Combination	10	
NCT02866383		Metastatic PaC and BTC	Nivolumab + Radiotherapy vs Nivolumab + Ipilimumab + Radiotherapy	Combination	160	
NCT02743494		Various advanced cancers	Nivolumab vs Placebo	Monotherapy	794	
NCT03752398		Solid tumors	XmAb^®^23104 vs XmAb^®^23104 + Ipilimumab	Combination	234	

*HCC* hepatocellular carcinoma, *GC* gastric cancer, *EC*: esophageal carcinoma, *GEJ* gastroesophgeal junction carcinoma, *CRC* colorectal cancer, *PaC* pancreatic cancer, *dMMR* miss match repair deficiency, *pMMR* proficient miss match repair, *MSS* microsatellite stable, *MSI-H* microsatellite instable-high, *SBRT* stereotactic body radiation therapy

Not only the type of GI cancer but also the type of drug is a determinant factor in ICI therapy. The results of the subgroup analysis showed that Pembrolizumab and Nivolumab are the most investigated drugs in GI cases; however, based on our analysis, Camrelizumab and Sintilimab outperformed the formers according to survival outcomes. All the abovementioned drugs target PD-1, implying that this molecule may probably be the more attractive ICI target rather than PD-L1 and CTLA-4 in GI cancer cases. Achieving the worst results with anti-PD-L1 in OS outcome, Avelumab may provide more data to probably conclude that PD-1 inhibitors are more efficient agents as compared to inhibitors of PD-L1. In agreement, Ribas et al. found that anti-PD-1 agents are superior inhibitors since they can inhibit PD-L2 binding to PD-1, as well [83].

Of great interest, the correlation between PD-L1 expression and the extent of the response to ICIs has been examined in a wide range of human malignancies; however, in many cases, there are conflicting results. While several previous studies indicated that the sensitivity extent of metastatic melanoma [84], non-small-cell lung cancer [11], and urothelial carcinoma [16] to ICIs correlates with the PD-L1 status, another study failed to report a differential sensitivity pattern with respect to PD-L1 expression level in renal carcinoma [13]. Based on our subgroup analysis, high expression of PD-L1 (either CPS or TPS) in GI cancers is associated with better response to ICIs. While, generally speaking, PD-L1 expression in GI cancers is a good biomarker for predicting ICI response, it is reasonable to hypothesize that different GI tumors ^__^given to their heterogeneous characteristics^__^ may respond differently based on PD-L1 status. In this regard, the promising results of KEYNOTE-180 and 181 trials which assessed the expression of PD-L1 using CPS led to the FDA approval of Pembrolizumab monotherapy in EC patients with CPS ≥ 10 after failure of first line of therapy [85, 86]. Our recent meta-analysis also revealed that PD-L1 CPS = 10 and TPS = 1% expression thresholds are predictive for lower rate of mortality when PD-1/PD-L1 inhibitors are administrated in patients suffering from EC [87]. Concerning GC, Kawazoe and Böger studies indicated that anti-PD-1/PD-L1 therapy is more effective in either MSI-high or EBV-positive advanced GC patients who are PD-L1 positive [88, 89]. Inline, the results of our recent meta-analysis reported that CPS scoring method has superior quality to TPS in predicting the response to ICIs; of note, GC patients expressing PD-L1 as CPS ≥ 1, CPS ≥ 5, and CPS ≥ 10 had longer OS than their counterpart subgroups (unpublished data). In contrast to EC and GC, PD-L1 expression did not seem to be a suitable biomarker for HCC and CRC patients as several trials revealed that ICI responses were observed regardless of PD-L1 expression in these patients [61, 78, 80]. Finally, the role of PD-L1 expression in PaC and BTC patients still remains unclear and further investigations are required to elucidate whether PD-L1 expression may influence the extent of PaC and BTC response to ICIs.

Another factor which affects GI tumors’ response to ICIs is microsatellite and mismatch repair (MMR) status. MSI-high and MMR deficiency (dMMR) in tumor cells may lead to high mutation levels and the arising of immunogenic neoantigens, facilitating their recognition by immune cells, and probably, a plausible justification for their better response to ICIs [90]. According to the importance of MSI-high status in some types of GI cancers, Pembrolizumab and Nivolumab achieved approval for dMMR/MSI-high CRC, EC, and GC patients [77–80]; however, in our analysis, a significant difference in survival outcomes between stable and unstable microsatellite status was only observed in GC patients. Of note, the results of a trial on BTC patients indicated that favorable responses to Nivolumab were unexpectedly observed in non-MSI-high patients [91]; further highlighting the necessity for additional studies to elaborate ICI responses with respect to microsatellite status in different GI tumors.

Albeit ICI is a promising treatment option at least in some GI cancers, our results demonstrate that serious AEs and AEs leading to death are more common in patients treated with ICIs compared with conventional therapies. In this regard, a meta-analysis showed that fatal AEs differ widely based on ICIs; while pneumonitis, hepatitis, and neurotoxic effects were the most frequent causes of death in patients receiving anti-PD-1 or anti-PD-L1, fatalities in patients receiving anti-CTLA-4 were mainly attributed to colitis. In combination therapy, the majority of ICI-related deaths were caused by myocarditis or colitis [92]. Therefore, early detection and management of these AEs are of paramount importance for practitioners.

We made our best efforts to present a complete and practical study, however, there are several limitations to be considered when interpreting the results or applying them in clinical practice. Firstly, some limitations were present due to the shortcomings of the included meta-analyses, such as heterogeneity of baseline characteristics, type of inhibitors, cycles of receiving the drug, different regimes in the control groups, and variable duration of follow-up which might influence some of the outcomes. Secondly, despite searching all of the databases mentioned above and searching extensively for related literature, there is still a possibility that some articles were missed. Thirdly, we were able to conduct subgroup analysis only on a few variables highlighting the need for trials with more subgroup analysis in the future. Fourthly, a definitive conclusion could not be reached in some analyses due to high confidence intervals.

**Fig. 8:**
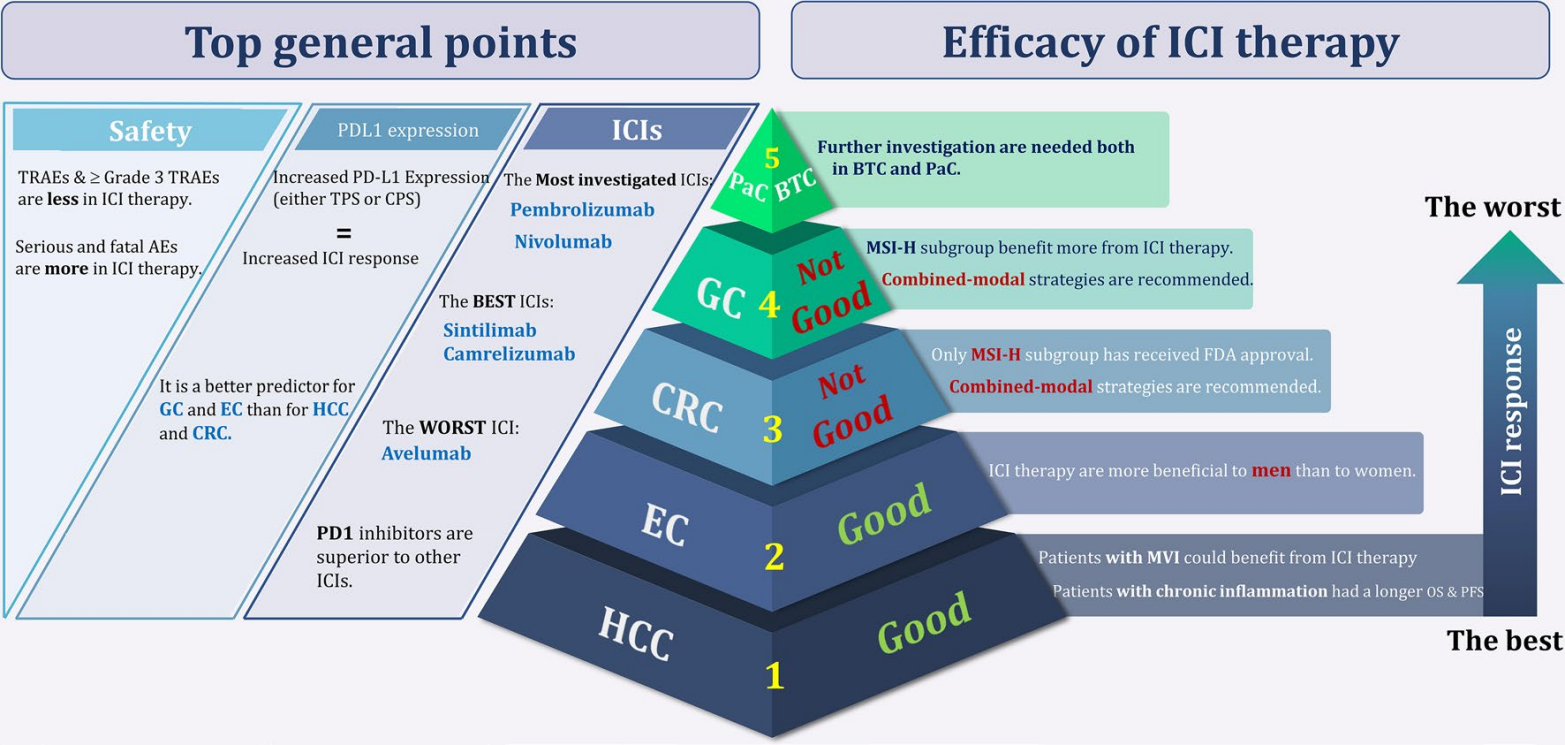
An overview of the ICI therapy in GI cancers

In conclusion, this article consolidates knowledge on one of the most promising treatment options for GI malignancies by providing a comprehensive review of the most recent evidence. According to our analysis, HCC and EC patients are likely better candidates for ICI therapy, especially PD-1 inhibitors, rather than GC and CRC patients. We also assess the predictive value of PD-L1 expression as well as microsatellite status in response to ICIs. In the near future, not only predictive biomarkers could be used to select which GI patients are more likely to benefit from ICIs, but they also can be utilized to support de-escalation of treatment in order to avoid unnecessary toxicity. PD-L1 expression and dMMR/MSI-H status are examples of these biomarkers, but _ as investigated in this study_ they have shown specific and narrow clinical applications. Priority in future research should be the identification of new clinically applicable prognostic and diagnostic biomarkers. Resistance to ICIs is another issue that must be addressed; for this purpose, combination therapy is currently being investigated to either alter the tumor microenvironment or target immune evasion mechanisms. Investigating the role of ICIs in adjuvant, neoadjuvant, and maintenance therapies should also be considered in future studies. Figure 8 provides an overview of the ICI therapy in GI cancers. It is likely that ICIs will become the standard of care in early lines of therapy for various GI malignancies as new data from ongoing trials emerge.

### Supplementary Information

**Additional file 1: Table S1. **Search strategy. **Table S2.** Definition of efficacy and safety outcomes. **Table S3. **Characteristics of included studies. **Table S4. **Methodological quality assessment by AMSTAR2. **Figure S1.** Forest plots of CR analysis in different types of GI cancers. **Figure S2.** Forest plots of PD analysis in different types of GI cancers. **Figure S3.** Forest plots of PR analysis in different types of GI cancers. **Figure S4.** Forest plots of SD analysis in different types of GI cancers.

## Data Availability

The data underlying this article are available in the article and in its online Additional file material.
