# Supplementary material for: Immune checkpoint inhibitors in gastrointestinal malignancies: an Umbrella review
Source: Cancer Cell Int. 2024 Jan 5;24:10. doi: 10.1186/s12935-023-03183-3 (PMC10771001; doi:10.1186/s12935-023-03183-3)
Supplement: Supplementary file 1 — Additional file 1: Table S1. Search strategy. Table S2. Definition of efficacy and safety outcomes. Table S3. Characteristics of included studies. Table S4. Methodological quality assessment by AMSTAR2. Figure S1. Forest plots of CR analysis in different types of GI cancers. Figure S2. Forest plots of PD analysis in different types of GI cancers. Figure S3. Forest plots of PR analysis in different types of GI cancers. Figure S4. Forest plots of SD analysis in different types of GI cancers. [file 12935_2023_3183_MOESM1_ESM.docx]

**Immune checkpoint inhibitors in gastrointestinal malignancies: An Umbrella review**

**Table S1.** Search strategy

| Database  (Search date) | Step | Search strategy | Number of results |
| --- | --- | --- | --- |
| PubMed  (9.1.2022) | #1 | “Gastrointestinal Malignanc*”[tiab] OR “Gastrointestinal Tumor*”[tiab] OR “Gastrointestinal Cancer*”[tiab] OR “Upper Gastrointestinal Malignanc*”[tiab] OR “Gastrointestinal Neoplasm*”[tiab] OR “Cancer of Gastrointestinal Tract”[tiab] OR “Gastrointestinal Tract Cancer*”[tiab] OR “Gastrointestinal Stromal Tumor*”[tiab] OR “Human Gastrointestinal Cancer*”[tiab] OR “GISTs”[tiab] OR “Esophago-Gastric tumor*”[tiab] OR “Esophago-Gastric Malignanc*”[tiab] OR “Esophago-Gastric cancer*”[tiab] OR “Esophagogastric tumor*”[tiab] OR “Esophagogastric Malignanc*”[tiab] OR “Esophagogastric cancer*”[tiab] OR “Esophagogastric neoplasm*”[tiab] OR “gastroesophageal tumor*”[tiab] OR “gastroesophageal Malignanc*”[tiab] OR “gastroesophageal cancer*”[tiab] OR “gastroesophageal neoplasm*”[tiab] OR “Gastrointestinal Tract”[tiab] OR “Advanced Gastrointestinal Cancer”[tiab] OR “gastric”[tiab] OR “esophageal”[tiab] OR “Oesophageal”[tiab] OR “Liver”[tiab] OR “colorect*”[tiab] OR “rectum”[tiab] OR “sigmoid”[tiab] OR “Anus”[tiab] OR “rectum”[tiab] OR “intestine”[tiab] OR “colon*”[tiab] OR “duodenum”[tiab] OR “duodenal”[tiab] OR “Jejunal”[tiab] OR “ileal”[tiab] OR “rectal”[tiab] OR “anal”[tiab] OR “jejunum”[tiab] OR “pancreas”[tiab] OR “Pancreatic”[tiab] OR “esophagogastric”[tiab] OR “esophago-gastric”[tiab] OR “cholangiocarcinoma”[tiab] OR “Hepatocellular”[tiab] OR “biliary”[tiab] OR “Hepatic”[tiab] OR “gastroesophageal”[tiab] OR “bile duct”[tiab] OR “Intestinal Neoplasm*”[tiab] OR “Intestine Neoplasm*”[tiab] OR “Cancer of Intestin*”[tiab] OR “Cancer of the Intestin*”[tiab] OR “Intestinal Cancer*”[tiab] OR “Secondary Gastrointestinal Cancer*”[tiab] OR “Cancer of stomach”[tiab] OR “Stomach neoplas*”[tiab] OR “Stomach cancer*”[tiab] OR “stomach tumor*”[tiab] OR “Cancer of the stomach”[tiab] OR “Gastroesophageal junction adenocarcinoma”[tiab] OR “Gastroesophageal junction tumor*”[tiab] OR “Gastroesophageal junction cancer*”[tiab] OR “Esophagogastric junction adenocarcinoma”[tiab] OR “Esophagogastric junction tumor*”[tiab] OR “Esophagogastric junction cancer*”[tiab] OR “Esophagogastric junction neoplasm*”[tiab] OR “Esophagogastric adenocarcinoma*”[tiab] OR “Esophageal cancer*”[tiab] OR “oesophageal cancer*”[tiab] OR “Esophageal neoplasm*”[tiab] OR “oesophageal neoplasm*”[tiab] OR “Esophageal tumor*”[tiab] OR “oesophageal tumor*”[tiab] OR “Esophageal adenoma*”[tiab] OR “Esophageal adenocarcinoma*”[tiab] OROR "oesophageal adenocarcinoma*"[tiab] OR "gastric lymphoma*"[tiab] OR "stomach carcinoma"[tiab] OR "gastric carcinoma"[tiab] OR "gastric adenocarcinoma"[tiab] OR "stomach adenocarcinoma"[tiab] OR "stomach lymphoma*"[tiab] OR "adenocarcinoma of the stomach"[tiab] OR "adenocarcinoma of stomach"[tiab] OR "adenoma of the stomach"[tiab] OR "adenocarcinoma of the gastroesophageal junction"[tiab] OR "adenocarcinoma of the esophagus"[tiab] OR "adenocarcinoma of esophagus"[tiab] OR "Gastroesophageal tumor*"[tiab] OR "Gastroesophageal cancer*"[tiab] OR "Gastroesophageal neoplasm*"[tiab] OR "Gastroesophageal adenocarcinoma*"[tiab] OR "Gastroesophageal carcinoma*"[tiab] OR "Gastro-esophageal cancer*"[tiab] OR "Gastro-esophageal adenocarcinoma*"[tiab] OR "cancer of stomach"[tiab] OR "neoplasm of stomach"[tiab] OR "tumor of stomach"[tiab] OR "cancer of esophagus"[tiab] OR "neoplasm of esophagus"[tiab] OR "tumor of esophagus"[tiab] OR "gastroesophageal junction"[tiab] OR "Esophageal Neoplasm*"[tiab] OR "Esophagus Neoplasm*"[tiab] OR "Cancer of Esophagus"[tiab] OR "Cancer of the Esophagus"[tiab] OR "Esophageal Squamous Cell Carcinoma*"[tiab] OR "Oesophageal Squamous Cell Carcinoma"[tiab] OR "Esophagus Cancer*"[tiab] OR "Stomach Neoplasm*"[tiab] OR "Colorectal Neoplasm*"[tiab] OR "Colorectal Cancer*"[tiab] OR "Colorectal Tumor*"[tiab] OR "Colorectal Carcinoma*"[tiab] OR "Gastric Cancer*"[tiab] OR "Hepatocellular Carcinoma*"[tiab] OR "Esophageal Cancer*"[tiab] OR "Oesophageal Cancer*"[tiab] OR "Gastroesophageal Cancer*"[tiab] OR "Distal Gastric Cancer*"[tiab] OR "Gastroesophageal Junction Malignanc*"[tiab] OR "Gastro-Oesophageal Junction Cancer*"[tiab] OR "Gastroesophageal Carcinoma*"[tiab] OR "Cancer Of The Gastroesophageal Junction"[tiab] OR "Esophageal Malignanc*"[tiab] OR "Oesophageal Malignanc*"[tiab] OR "Esophageal Neoplasia*"[tiab] OR "Esophageal Neoplasm*"[tiab] OR "Cancer Of The Esophagus"[tiab] OR "Cancer Of The Stomach"[tiab] OR "Cancer Of The Colon"[tiab] OR "Cancer Of Colon"[tiab] OR "Upper Gastrointestinal Cancer*"[tiab] OR "Liver Cancer*"[tiab] OR "Hepatocellular Carcinoma*"[tiab] OR "Hepatic Cancer*"[tiab] OR "Hepatic neoplasm*"[tiab] OR "Primary Hepatic Cancer*"[tiab] OR "Hepatic Malignanc*"[tiab] OR "Malignant Liver Tumor*"[tiab] OR "Liver Malignanc*"[tiab] OR "Metastatic Liver Tumor*"[tiab] OR "Primary And Secondary Liver Malignanc*"[tiab] OR "Hepatoblastoma"[tiab] OR "Hepatocellular Cancer*"[tiab] OR "Cancer of Liver"[tiab] OR "Percutaneous Liver Tumor*"[tiab] OR "Malignant Liver Tumor*"[tiab] OR "Metastatic Liver Tumor*"[tiab] OR " Liver Neoplasm*"[tiab] OR "Primary Liver Neoplasm*"[tiab] OR "Malignant Liver Neoplasm*"[tiab] OR "Liver Neoplasia*"[tiab] OR "Hepatic Neoplasia*"[tiab] OR "Duodenal Cancer*"[tiab] OR "Cancer Of The Duodenum"[tiab] OR "Small Intestine Cancer*"[tiab] OR "Duodenal Tumor*"[tiab] OR "Duodenal Malignanc*"[tiab] OR "Stromal Tumor*"[tiab] OR "Duodenal Neoplasm*"[tiab] OR "Duodenal Epithelial Neoplasm*"[tiab] OR "Small Bowel Cancer*"[tiab] OR "Cancer Of The Jejunum"[tiab] OR "Villous Tumors"[tiab] OR "Jejunal Carcinoma*"[tiab] OR "Jejunum Cancer"[tiab] OR "Jejunal Neoplasm*"[tiab] OR "Jejunal cancer*"[tiab] OR "Jejunal Malignant Lymphoma"[tiab] OR "Primary Jejunal Adenocarcinoma*"[tiab] OR "Ileum Cancer*"[tiab] OR "Tumor Of The Ileum"[tiab] OR "Carcinoid Tumor Of The Ileum"[tiab] OR "Stromal Tumor*"[tiab] OR "Malignant Glomus Tumor*"[tiab] OR "Proximal Colon Cancer*"[tiab] OR "Neoplasm Of the Small Intestine"[tiab] OR "Ileal Neuroendocrine Neoplasm*"[tiab] OR "Ileal Lymphoma"[tiab] OR "Pancreatic Neoplasm*"[tiab] OR "Pancreatic Cancer*"[tiab] OR "Advanced Pancreatic Cancer*"[tiab] OR "Pancreatic Tumor*"[tiab] OR "Pancreatic Malignanc*"[tiab] OR "Hepatobiliary Pancreatic Malignanc*"[tiab] OR "Pancreatic Ductal Adenocarcinoma*"[tiab] OR "Pancreatic Neoplasm*"[tiab] OR "Pancreas cancer*"[tiab] OR "Pancreas neoplasm*"[tiab] OR "Pancreas carcinoma*"[tiab] OR "Pancreas neoplasia*"[tiab] OR "Anal Cancer*"[tiab] OR "Anal neoplasm*"[tiab] OR "Anus neoplasm*"[tiab] OR "Cancer of the Anus"[tiab] OR "Cancer of Anus"[tiab] OR "Colon Cancer*"[tiab] OR "Colorectal Cancer*"[tiab] OR "Rectal Cancer*"[tiab] OR "Rectal Tumor*"[tiab] OR "Rectal Neoplasm*"[tiab] OR "Rectal Neoplasia*"[tiab] OR "Rectum cancer*"[tiab] OR "Cancer of the Rectum"[tiab] OR "Cancer of Rectum"[tiab] OR "Anus Cancer*"[tiab] OR "Carcinoma Of The Colon"[tiab] OR "Tumor Of The Colon"[tiab] OR "Anal Malignanc*"[tiab] OR "Anal Carcinoma*"[tiab] OR "Malignant Colonic Obstruction"[tiab] OR "Neoplasm Of The Anus"[tiab] OR "Colon cancer*"[tiab] OR "Colon Adenocarcinoma"[tiab] OR "Intrahepatic cholangiocarcinoma*"[tiab] OR "Biliary Cancer*"[tiab] OR "Gallbladder Cancer*"[tiab] OR "Gallbladder Neoplasm*"[tiab] OR "Biliary Tract Cancer*"[tiab] OR "Biliary Tract Carcinoma*"[tiab] OR "Cancer of the Gallbladder"[tiab] OR "Biliary Tract Malignanc*"[tiab] OR "Biliary Malignanc*"[tiab] OR "Bile Duct Cancer*"[tiab] OR "Gallbladder Cancer*"[tiab] OR "Gallbladder And Bile Duct Neoplasm*"[tiab] OR "Biliary Tract Neoplasm*"[tiab] OR "Biliary Neoplasm*"[tiab] OR "Gallbladder Neoplasm*"[tiab] OR "Neoplasm Of The Biliary Tract"[tiab] OR "Neoplasm of Gallbladder"[tiab] OR "Neoplasm Of The Gallbladder"[tiab] OR "Tumor Of The Gallbladder"[tiab] OR "Biliary Tract tumor*"[tiab] OR "Biliary Intraepithelial Neoplasia*"[tiab] OR "Gallbladder Neoplasia*"[tiab] OR "Biliary Neoplasia*"[tiab] OR "Gallbladder Carcinoma*"[tiab] OR "Biliary Tract Intraepithelial Neoplasia*"[tiab] OR "Small bowel cancer*"[tiab] OR "Small Intestine Cancer*"[tiab] OR "Cancer Of The Small Intestine"[tiab] OR "Small Intestine Cancer*"[tiab] OR "Small Intestinal Tumor*"[tiab] OR "Adenocarcinoma Of The Small Intestine"[tiab] OR "Small Intestinal Neoplasm*"[tiab] OR "Lymphoma Of The Small Intestine"[tiab] OR "Small Intestinal Neoplasm*"[tiab] OR "Small Intestinal Neoplasm*"[tiab] OR "Neoplasm Of The Small Intestine"[tiab] OR "Small Intestinal Neoplasia*"[tiab] OR "Colorectal Cancer"[tiab] OR "Colorectal Tumor*"[tiab] OR "Colorectal Malignanc*"[tiab] OR "Colorectal Neoplasm*"[tiab] OR "Colorectal Neoplasia*"[tiab] OR "Advanced Colorectal Neoplasia*"[tiab] OR "Colorectal Neoplasia"[tiab] OR "Transverse Colon Cancer*"[tiab] OR "Transverse Colon Cancer*"[tiab] OR "Transverse Colon Carcinoma*"[tiab] OR "Transverse Colon Neoplasm*"[tiab] OR "Descending Colon Cancer*"[tiab] OR "Ascending Colon Cancer*"[tiab] OR "Descending Colon Tumor*"[tiab] OR "Ascending Colon Tumor*"[tiab] OR "Left Sided Colonic Tumor*"[tiab] OR "Ascending Colon Cancer*"[tiab] OR "Sigmoid Colon Cancer*"[tiab] OR "Sigmoid Colon Tumor*"[tiab] OR "Adenocarcinoma of Sigmoid Colon"[tiab] OR "Rectosigmoid Adenomas"[tiab] OR "Cecum Cancer*"[tiab] OR "Cancer of Cecum"[tiab] OR "Cecal Tumor*"[tiab] OR "Cecum Tumor*"[tiab] OR "Cecal Malignanc*"[tiab] OR "Carcinoma Of The Cecum"[tiab] OR "Cecal Carcinoma*"[tiab] OR "Cecal Neoplasm*"[tiab] OR "Colon And Rectum Cancer*"[tiab] OR "Rectum Cancer*"[tiab] OR "Cancer Of The Colon And Rectum"[tiab] OR "Rectal Tumor*"[tiab] OR "Rectal Malignanc*"[tiab] OR "Primary Rectal Malignanc*"[tiab] OR "Rectal Malignant Tumor*"[tiab] OR "Rectal Neoplasm*"[tiab] OR "Rectal Neoplasia*"[tiab] OR "Appendiceal Neoplasm*"[tiab] OR "Familial Polyposis Syndrome*"[tiab] OR "Polypos*"[tiab] OR "colonic neoplasm*"[tiab] OR "colonic cancer*"[tiab] OR "Sigmoid Neoplasm*"[tiab] OR "Sigmoid Colon Neoplasm*"[tiab] OR "Sigmoidal Cancer*"[tiab] OR "Cancer of Sigmoid"[tiab] OR "Cancer of the Sigmoid"[tiab] OR "Rectal Tumor*"[tiab] OR "Bile Duct Neoplasm*"[tiab] OR "Gastrointestinal Neoplasms"[MeSH] OR "Esophageal Neoplasms"[MeSH] OR "Esophageal Squamous Cell Carcinoma"[MeSH] OR "Intestinal Neoplasms"[MeSH] OR "Cecal Neoplasms"[MeSH] OR "Appendiceal Neoplasms"[MeSH] OR "Colorectal Neoplasms"[MeSH] OR "Adenomatous Polyposis Coli"[MeSH] OR "Colonic Neoplasms"[MeSH] OR "Sigmoid Neoplasms"[MeSH] OR "Colorectal Neoplasms, Hereditary Nonpolyposis"[MeSH] OR "Rectal Neoplasms"[MeSH] OR "Anus Neoplasms"[MeSH] OR "Duodenal Neoplasms"[MeSH] OR "Ileal Neoplasms"[MeSH] OR "Jejunal Neoplasms"[MeSH] OR "Stomach Neoplasms"[MeSH] OR "Liver Neoplasms"[MeSH] OR "Carcinoma, Hepatocellular"[MeSH] OR "Adenoma, Liver Cell"[MeSH] OR "Liver Neoplasms, Experimental"[MeSH] OR "Pancreatic Neoplasms"[MeSH] OR "Carcinoma, Pancreatic Ductal"[MeSH] OR "Pancreatic Intraductal Neoplasms"[MeSH] OR "Gallbladder Neoplasms"[MeSH] OR "Bile Duct Neoplasms"[MeSH] | 846,731 |
|  | #2 | “immune Checkpoint Inhibitors"[MeSH] OR "Immune Checkpoint Inhibitor*"[tiab] OR "Immune Checkpoint Blocker*"[tiab] OR "Immune Checkpoint Blockade*"[tiab] OR "Immune Checkpoint Inhibition*"[tiab] OR "PD-L1 Inhibitor*"[tiab] OR "PD L1 Inhibitor*"[tiab] OR "PDL1 Inhibitor*"[tiab] OR "PD-L1 blocker*"[tiab] OR "PD L1 blocker*"[tiab] OR "Programmed Death-Ligand 1 Inhibitor*"[tiab] OR "Programmed Death Ligand 1 Inhibitor*"[tiab] OR "anti Programmed Death-Ligand 1"[tiab] OR "anti-Programmed Death-Ligand 1"[tiab] OR "anti Programmed Death Ligand 1"[tiab] OR "anti-Programmed Death Ligand 1"[tiab] OR "CTLA-4 Inhibitor*"[tiab] OR "CTLA 4 Inhibitor*"[tiab] OR "CTLA4 Inhibitor*"[tiab] OR "CTLA-4 blocker*"[tiab] OR "CTLA 4 blocker*"[tiab] OR "CTLA4 blocker*"[tiab] OR "Cytotoxic T-Lymphocyte Associated Protein 4 Inhibitor*"[tiab] OR "Cytotoxic T Lymphocyte-Associated Protein 4 Inhibitor*"[tiab] OR "Cytotoxic T-Lymphocyte Associated antigen 4 Inhibitor*"[tiab] OR "Cytotoxic T Lymphocyte-Associated antigen 4 Inhibitor*"[tiab] OR "Cytotoxic T-Lymphocyte-Associated Protein 4 Inhibitor*"[tiab] OR "Cytotoxic T Lymphocyte Associated Protein 4 Inhibitor*"[tiab] OR "Cytotoxic T-Lymphocyte-Associated antigen 4 Inhibitor*"[tiab] OR "Cytotoxic T Lymphocyte Associated antigen 4 Inhibitor*"[tiab] OR "anti-cytotoxic T lymphocyte-associated antigen 4"[tiab] OR "anti cytotoxic T lymphocyte-associated antigen 4"[tiab] OR "anti-cytotoxic T lymphocyte-associated protein 4"[tiab] OR "anti cytotoxic T lymphocyte-associated protein 4"[tiab] OR "anti-cytotoxic T-lymphocyte-associated antigen 4"[tiab] OR "anti-cytotoxic T-lymphocyte-associated antigen 4"[tiab] OR "PD-1 Inhibitor*"[tiab] OR "PD 1 Inhibitor*"[tiab] OR "PD1 Inhibitor*"[tiab] OR "PD 1 blocker*"[tiab] OR "PD-1 blocker*"[tiab] OR "Programmed Cell Death Protein 1 Inhibitor*"[tiab] OR "anti Programmed Cell Death Protein 1"[tiab] OR "anti-Programmed Cell Death Protein 1"[tiab] OR "anti-PD1"[tiab] OR "anti PD1"[tiab] OR "anti-PD-1"[tiab] OR "anti PD-1"[tiab] OR "anti-PD-L1"[tiab] OR "anti PDL1"[tiab] OR "anti PD-L1"[tiab] OR "anti-PDL1"[tiab] OR "anti-PD L1"[tiab] OR "anti PD L1"[tiab] OR "anti-CTLA4"[tiab] OR "anti-CTLA 4"[tiab] OR "anti-CTLA-4"[tiab] OR "anti CTLA4"[tiab] OR "anti CTLA 4"[tiab] OR "anti CTLA-4"[tiab] OR "PD-1-PD-L1 Blockade*"[tiab] OR "PD 1 PD L1 Blockade*"[tiab] OR "Pembrolizumab"[tiab] OR "MK-3475"[tiab] OR "lambrolizumab"[tiab] OR "Keytruda"[tiab] OR "SCH-900475"[tiab] OR "Nivolumab"[tiab] OR "Opdivo"[tiab] OR "ONO-4538"[tiab] OR "ONO 4538"[tiab] OR "ONO4538"[tiab] OR "MDX-1106"[tiab] OR "MDX 1106"[tiab] OR "MDX1106"[tiab] OR "BMS-936558"[tiab] OR "BMS 936558"[tiab] OR "BMS936558"[tiab] OR "Ipilimumab"[tiab] OR "Yervoy"[tiab] OR "MDX 010"[tiab] OR "MDX010"[tiab] OR "MDX-010"[tiab] OR "MDX-CTLA-4"[tiab] OR "MDX CTLA 4"[tiab] OR "Durvalumab"[tiab] OR "MEDI4736"[tiab] OR "MEDI-4736"[tiab] OR "Imfinzi"[tiab] OR "Dostarlimab"[tiab] OR "TSR-042"[tiab] OR "Cemiplimab"[tiab] OR "REGN2810"[tiab] OR "Avelumab"[tiab] OR "MSB0010682"[tiab] OR "bavencio"[tiab] OR "MSB0010718C"[tiab] OR "MSB-0010718C"[tiab] OR "Atezolizumab"[tiab] OR "MPDL3280A"[tiab] OR "MPDL-3280A"[tiab] OR "Tecentriq"[tiab] OR "RG7446"[tiab] OR "RG-7446"[tiab] OR "pidilizumab"[tiab] OR "CT-011"[tiab] OR "CT 011"[tiab] OR "tremelimumab"[tiab] OR "ticilimumab"[tiab] OR "CP 675"[tiab] OR "CP-675"[tiab] OR "CP-675,206"[tiab] OR "CP-675206"[tiab] OR "CP675206"[tiab] OR "CP 675206"[tiab] OR "dostarlimab"[Supplementary Concept] OR "cemiplimab"[Supplementary Concept] OR "Ipilimumab"[Mesh] OR "tremelimumab"[Supplementary Concept] OR "pembrolizumab"[Supplementary Concept] OR "atezolizumab"[Supplementary Concept] OR "Nivolumab"[Mesh] OR "durvalumab"[Supplementary Concept] OR "avelumab" [Supplementary Concept] OR "pidilizumab" [Supplementary Concept] OR "sintilimab"[tiab] OR "camrelizumab"[tiab] OR "toripalimab"[tiab] OR "HX008"[tiab] OR "tremelimumab"[tiab] OR "PD-1"[tiab] OR "PD-L1"[tiab] OR "CTLA-4"[tiab] OR "PD-1/PD-L1"[tiab] OR "PD 1"[tiab] OR "PD L1"[tiab] OR "CTLA 4"[tiab] | 61,216 |
|  | #3 | “meta-analysis”[pt] OR “meta-analysis”[tiab] OR “meta analysis”[tiab] OR "meta-analyses"[tiab] OR "meta analyses"[tiab] OR "meta-analyze"[tiab] OR "meta analyze"[tiab] OR "metaanalysis"[tiab] OR “metaanalyze”[tiab] OR “Network Meta-Analysis”[mh] OR “Systematic Review”[pt] OR “Systematic Review”[tiab] OR “pooled analysis”[tiab] | 405,646 |
|  | #4 | #1 AND #2 AND #3 | 249 |
| Scopus  (9.1.2022) | #1 | TITLE-ABS-KEY(“Gastrointestinal Malignanc*” OR “Gastrointestinal Tumor*” OR “Gastrointestinal Cancer*” OR “Upper Gastrointestinal Malignanc*” OR “Gastrointestinal Neoplasm*” OR “Cancer of Gastrointestinal Tract” OR “Gastrointestinal Tract Cancer*” OR “Gastrointestinal Stromal Tumor*” OR “Human Gastrointestinal Cancer*” OR “GISTs” OR “Esophago-Gastric tumor*” OR “Esophago-Gastric Malignanc*” OR “Esophago-Gastric cancer*” OR “Esophagogastric tumor*” OR “Esophagogastric Malignanc*” OR “Esophagogastric cancer*” OR “Esophagogastric neoplasm*” OR “gastroesophageal tumor*” OR “gastroesophageal Malignanc*” OR “gastroesophageal cancer*” OR “gastroesophageal neoplasm*” OR “Gastrointestinal Tract” OR “Advanced Gastrointestinal Cancer” OR “gastric” OR “esophageal” OR “Oesophageal” OR “Liver” OR “colorect*” OR “rectum” OR “sigmoid” OR “Anus” OR “rectum” OR “intestine” OR “colon*” OR “duodenum” OR “duodenal” OR “Jejunal” OR “ileal” OR “rectal” OR “anal” OR “jejunum” OR “pancreas” OR “Pancreatic” OR “esophagogastric” OR “esophago-gastric” OR “cholangiocarcinoma” OR “Hepatocellular” OR “biliary” OR “Hepatic” OR “gastroesophageal” OR “bile duct” OR “Intestinal Neoplasm*” OR “Intestine Neoplasm*” OR “Cancer of Intestin*” OR “Cancer of the Intestin*” OR “Intestinal Cancer*” OR “Secondary Gastrointestinal Cancer*” OR “Cancer of stomach” OR “Stomach neoplas*” OR “Stomach cancer*” OR “stomach tumor*” OR “Cancer of the stomach” OR “Gastroesophageal junction adenocarcinoma” OR “Gastroesophageal junction tumor*” OR “Gastroesophageal junction cancer*” OR “Esophagogastric junction adenocarcinoma” OR “Esophagogastric junction tumor*” OR “Esophagogastric junction cancer*” OR “Esophagogastric junction neoplasm*” OR “Esophagogastric adenocarcinoma*” OR “Esophageal cancer*” OR “oesophageal cancer*” OR “Esophageal neoplasm*” OR “oesophageal neoplasm*” OR “Esophageal tumor*” OR “oesophageal tumor*” OR “Esophageal adenoma*” OR “Esophageal adenocarcinoma*” OR "oesophageal adenocarcinoma*" OR "gastric lymphoma*" OR "stomach carcinoma" OR "gastric carcinoma" OR "gastric adenocarcinoma" OR "stomach adenocarcinoma" OR "stomach lymphoma*" OR "adenocarcinoma of the stomach" OR "adenocarcinoma of stomach" OR "adenoma of the stomach" OR "adenocarcinoma of the gastroesophageal junction" OR "adenocarcinoma of the esophagus" OR "adenocarcinoma of esophagus" OR "Gastroesophageal tumor*" OR "Gastroesophageal cancer*" OR "Gastroesophageal neoplasm*" OR "Gastroesophageal adenocarcinoma*" OR "Gastroesophageal carcinoma*" OR "Gastro-esophageal cancer*" OR "Gastro-esophageal adenocarcinoma*" OR "cancer of stomach" OR "neoplasm of stomach" OR "tumor of stomach" OR "cancer of esophagus" OR "neoplasm of esophagus" OR "tumor of esophagus" OR "gastroesophageal junction" OR "Esophageal Neoplasm*" OR "Esophagus Neoplasm*" OR "Cancer of Esophagus" OR "Cancer of the Esophagus" OR "Esophageal Squamous Cell Carcinoma*" OR "Oesophageal Squamous Cell Carcinoma" OR "Esophagus Cancer*" OR "Stomach Neoplasm*" OR "Colorectal Neoplasm*" OR "Colorectal Cancer*" OR "Colorectal Tumor*" OR "Colorectal Carcinoma*" OR "Gastric Cancer*" OR "Hepatocellular Carcinoma*" OR "Esophageal Cancer*" OR "Oesophageal Cancer*" OR "Gastroesophageal Cancer*" OR "Distal Gastric Cancer*" OR "Gastroesophageal Junction Malignanc*" OR "Gastro-Oesophageal Junction Cancer*" OR "Gastroesophageal Carcinoma*" OR "Cancer Of The Gastroesophageal Junction" OR "Esophageal Malignanc*" OR "Oesophageal Malignanc*" OR "Esophageal Neoplasia*" OR "Esophageal Neoplasm*" OR "Cancer Of The Esophagus" OR "Cancer Of The Stomach" OR "Cancer Of The Colon" OR "Cancer Of Colon" OR "Upper Gastrointestinal Cancer*" OR "Liver Cancer*" OR "Hepatocellular Carcinoma*" OR "Hepatic Cancer*" OR "Hepatic neoplasm*" OR "Primary Hepatic Cancer*" OR "Hepatic Malignanc*" OR "Malignant Liver Tumor*" OR "Liver Malignanc*" OR "Metastatic Liver Tumor*" OR "Primary And Secondary Liver Malignanc*" OR "Hepatoblastoma" OR "Hepatocellular Cancer*" OR "Cancer of Liver" OR "Percutaneous Liver Tumor*" OR "Malignant Liver Tumor*" OR "Metastatic Liver Tumor*" OR " Liver Neoplasm*" OR "Primary Liver Neoplasm*" OR "Malignant Liver Neoplasm*" OR "Liver Neoplasia*" OR "Hepatic Neoplasia*" OR "Duodenal Cancer*" OR "Cancer Of The Duodenum" OR "Small Intestine Cancer*" OR "Duodenal Tumor*" OR "Duodenal Malignanc*" OR "Stromal Tumor*" OR "Duodenal Neoplasm*" OR "Duodenal Epithelial Neoplasm*" OR "Small Bowel Cancer*" OR "Cancer Of The Jejunum" OR "Villous Tumors" OR "Jejunal Carcinoma*" OR "Jejunum Cancer" OR "Jejunal Neoplasm*" OR "Jejunal cancer*" OR "Jejunal Malignant Lymphoma" OR "Primary Jejunal Adenocarcinoma*" OR "Ileum Cancer*" OR "Tumor Of The Ileum" OR "Carcinoid Tumor Of The Ileum" OR "Stromal Tumor*" OR "Malignant Glomus Tumor*" OR "Proximal Colon Cancer*" OR "Neoplasm Of the Small Intestine" OR "Ileal Neuroendocrine Neoplasm*" OR "Ileal Lymphoma" OR "Pancreatic Neoplasm*" OR "Pancreatic Cancer*" OR "Advanced Pancreatic Cancer*" OR "Pancreatic Tumor*" OR "Pancreatic Malignanc*" OR "Hepatobiliary Pancreatic Malignanc*" OR "Pancreatic Ductal Adenocarcinoma*" OR "Pancreatic Neoplasm*" OR "Pancreas cancer*" OR "Pancreas neoplasm*" OR "Pancreas carcinoma*" OR "Pancreas neoplasia*" OR "Anal Cancer*" OR "Anal neoplasm*" OR "Anus neoplasm*" OR "Cancer of the Anus" OR "Cancer of Anus" OR "Colon Cancer*" OR "Colorectal Cancer*" OR "Rectal Cancer*" OR "Rectal Tumor*" OR "Rectal Neoplasm*" OR "Rectal Neoplasia*" OR "Rectum cancer*" OR "Cancer of the Rectum" OR "Cancer of Rectum" OR "Anus Cancer*" OR "Carcinoma Of The Colon" OR "Tumor Of The Colon" OR "Anal Malignanc*" OR "Anal Carcinoma*" OR "Malignant Colonic Obstruction" OR "Neoplasm Of The Anus" OR "Colon cancer*" OR "Colon Adenocarcinoma" OR "Intrahepatic cholangiocarcinoma*" OR "Biliary Cancer*" OR "Gallbladder Cancer*" OR "Gallbladder Neoplasm*" OR "Biliary Tract Cancer*" OR "Biliary Tract Carcinoma*" OR "Cancer of the Gallbladder" OR "Biliary Tract Malignanc*" OR "Biliary Malignanc*" OR "Bile Duct Cancer*" OR "Gallbladder Cancer*" OR "Gallbladder And Bile Duct Neoplasm*" OR "Biliary Tract Neoplasm*" OR "Biliary Neoplasm*" OR "Gallbladder Neoplasm*" OR "Neoplasm Of The Biliary Tract" OR "Neoplasm of Gallbladder" OR "Neoplasm Of The Gallbladder" OR "Tumor Of The Gallbladder" OR "Biliary Tract tumor*" OR "Biliary Intraepithelial Neoplasia*" OR "Gallbladder Neoplasia*" OR "Biliary Neoplasia*" OR "Gallbladder Carcinoma*" OR "Biliary Tract Intraepithelial Neoplasia*" OR "Small bowel cancer*" OR "Small Intestine Cancer*" OR "Cancer Of The Small Intestine" OR "Small Intestine Cancer*" OR "Small Intestinal Tumor*" OR "Adenocarcinoma Of The Small Intestine" OR "Small Intestinal Neoplasm*" OR "Lymphoma Of The Small Intestine" OR "Small Intestinal Neoplasm*" OR "Small Intestinal Neoplasm*" OR "Neoplasm Of The Small Intestine" OR "Small Intestinal Neoplasia*" OR "Colorectal Cancer" OR "Colorectal Tumor*" OR "Colorectal Malignanc*" OR "Colorectal Neoplasm*" OR "Colorectal Neoplasia*" OR "Advanced Colorectal Neoplasia*" OR "Colorectal Neoplasia" OR "Transverse Colon Cancer*" OR "Transverse Colon Cancer*" OR "Transverse Colon Carcinoma*" OR "Transverse Colon Neoplasm*" OR "Descending Colon Cancer*" OR "Ascending Colon Cancer*" OR "Descending Colon Tumor*" OR "Ascending Colon Tumor*" OR "Left Sided Colonic Tumor*" OR "Ascending Colon Cancer*" OR "Sigmoid Colon Cancer*" OR "Sigmoid Colon Tumor*" OR "Adenocarcinoma of Sigmoid Colon" OR "Rectosigmoid Adenomas" OR "Cecum Cancer*" OR "Cancer of Cecum" OR "Cecal Tumor*" OR "Cecum Tumor*" OR "Cecal Malignanc*" OR "Carcinoma Of The Cecum" OR "Cecal Carcinoma*" OR "Cecal Neoplasm*" OR "Colon And Rectum Cancer*" OR "Rectum Cancer*" OR "Cancer Of The Colon And Rectum" OR "Rectal Tumor*" OR "Rectal Malignanc*" OR "Primary Rectal Malignanc*" OR "Rectal Malignant Tumor*" OR "Rectal Neoplasm*" OR "Rectal Neoplasia*" OR "Appendiceal Neoplasm*" OR "Familial Polyposis Syndrome*" OR "Polypos*" OR "colonic neoplasm*" OR "colonic cancer*" OR "Sigmoid Neoplasm*" OR "Sigmoid Colon Neoplasm*" OR "Sigmoidal Cancer*" OR "Cancer of Sigmoid" OR "Cancer of the Sigmoid" OR "Rectal Tumor*" OR "Bile Duct Neoplasm*" OR "Gastrointestinal Neoplasms" OR "Esophageal Neoplasms" OR "Esophageal Squamous Cell Carcinoma" OR "Intestinal Neoplasms" OR "Cecal Neoplasms" OR "Appendiceal Neoplasms" OR "Colorectal Neoplasms" OR "Adenomatous Polyposis Coli" OR "Colonic Neoplasms" OR "Sigmoid Neoplasms" OR "Colorectal Neoplasms, Hereditary Nonpolyposis" OR "Rectal Neoplasms" OR "Anus Neoplasms" OR "Duodenal Neoplasms" OR "Ileal Neoplasms" OR "Jejunal Neoplasms" OR "Stomach Neoplasms" OR "Liver Neoplasms" OR "Carcinoma, Hepatocellular" OR "Adenoma, Liver Cell" OR "Liver Neoplasms, Experimental" OR "Pancreatic Neoplasms" OR "Carcinoma, Pancreatic Ductal" OR "Pancreatic Intraductal Neoplasms" OR "Gallbladder Neoplasms" OR "Bile Duct Neoplasms") | 4,572,280 |
|  | #2 | TITLE-ABS-KEY(“immune Checkpoint Inhibitors" OR "Immune Checkpoint Inhibitor*" OR "Immune Checkpoint Blocker*" OR "Immune Checkpoint Blockade*" OR "Immune Checkpoint Inhibition*" OR "PD-L1 Inhibitor*" OR "PD L1 Inhibitor*" OR "PDL1 Inhibitor*" OR "PD-L1 blocker*" OR "PD L1 blocker*" OR "Programmed Death-Ligand 1 Inhibitor*" OR "Programmed Death Ligand 1 Inhibitor*" OR "anti Programmed Death-Ligand 1" OR "anti-Programmed Death-Ligand 1" OR "anti Programmed Death Ligand 1" OR "anti-Programmed Death Ligand 1" OR "CTLA-4 Inhibitor*" OR "CTLA 4 Inhibitor*" OR "CTLA4 Inhibitor*" OR "CTLA-4 blocker*" OR "CTLA 4 blocker*" OR "CTLA4 blocker*" OR "Cytotoxic T-Lymphocyte Associated Protein 4 Inhibitor*" OR "Cytotoxic T Lymphocyte-Associated Protein 4 Inhibitor*" OR "Cytotoxic T-Lymphocyte Associated antigen 4 Inhibitor*" OR "Cytotoxic T Lymphocyte-Associated antigen 4 Inhibitor*" OR "Cytotoxic T-Lymphocyte-Associated Protein 4 Inhibitor*" OR "Cytotoxic T Lymphocyte Associated Protein 4 Inhibitor*" OR "Cytotoxic T-Lymphocyte-Associated antigen 4 Inhibitor*" OR "Cytotoxic T Lymphocyte Associated antigen 4 Inhibitor*" OR "anti-cytotoxic T lymphocyte-associated antigen 4" OR "anti cytotoxic T lymphocyte-associated antigen 4" OR "anti-cytotoxic T lymphocyte-associated protein 4" OR "anti cytotoxic T lymphocyte-associated protein 4" OR "anti-cytotoxic T-lymphocyte-associated antigen 4" OR "anti-cytotoxic T-lymphocyte-associated antigen 4" OR "PD-1 Inhibitor*" OR "PD 1 Inhibitor*" OR "PD1 Inhibitor*" OR "PD 1 blocker*" OR "PD-1 blocker*" OR "Programmed Cell Death Protein 1 Inhibitor*" OR "anti Programmed Cell Death Protein 1" OR "anti-Programmed Cell Death Protein 1" OR "anti-PD1" OR "anti PD1" OR "anti-PD-1" OR "anti PD-1" OR "anti-PD-L1" OR "anti PDL1" OR "anti PD-L1" OR "anti-PDL1" OR "anti-PD L1" OR "anti PD L1" OR "anti-CTLA4" OR "anti-CTLA 4" OR "anti-CTLA-4" OR "anti CTLA4" OR "anti CTLA 4" OR "anti CTLA-4" OR "PD-1-PD-L1 Blockade*" OR "PD 1 PD L1 Blockade*" OR "Pembrolizumab" OR "MK-3475" OR "lambrolizumab" OR "Keytruda" OR "SCH-900475" OR "Nivolumab" OR "Opdivo" OR "ONO-4538" OR "ONO 4538" OR "ONO4538" OR "MDX-1106" OR "MDX 1106" OR "MDX1106" OR "BMS-936558" OR "BMS 936558" OR "BMS936558" OR "Ipilimumab" OR "Yervoy" OR "MDX 010" OR "MDX010" OR "MDX-010" OR "MDX-CTLA-4" OR "MDX CTLA 4" OR "Durvalumab" OR "MEDI4736" OR "MEDI-4736" OR "Imfinzi" OR "Dostarlimab" OR "TSR-042" OR "Cemiplimab" OR "REGN2810" OR "Avelumab" OR "MSB0010682" OR "bavencio" OR "MSB0010718C" OR "MSB-0010718C" OR "Atezolizumab" OR "MPDL3280A" OR "MPDL-3280A" OR "Tecentriq" OR "RG7446" OR "RG-7446" OR "pidilizumab" OR "CT-011" OR "CT 011" OR "tremelimumab" OR "ticilimumab" OR "CP 675" OR "CP-675" OR "CP-675,206" OR "CP-675206" OR "CP675206" OR "CP 675206" OR "dostarlimab" OR "cemiplimab" OR "Ipilimumab" OR "tremelimumab" OR "pembrolizumab" OR "atezolizumab" OR "Nivolumab" OR "durvalumab" OR "avelumab" OR "pidilizumab" OR "sintilimab" OR "camrelizumab" OR "toripalimab" OR "HX008" OR "tremelimumab" OR "PD-1" OR "PD-L1" OR "CTLA-4" OR "PD-1/PD-L1" OR "PD 1" OR "PD L1" OR "CTLA 4") | 81,178 |
|  | #3 | TITLE-ABS-KEY(“meta-analysis” OR “meta analysis” OR "meta-analyses" OR "meta analyses" OR "meta-analyze" OR "metaanalysis" OR “metaanalyze” OR “Network Meta-Analysis” OR “Systematic Review” OR “pooled analysis”) | 595,954 |
|  | #4 | #1 AND #2 AND #3 | 1,087 |
| Web of Science  (9.1.2022) | #1 | TS=(“Gastrointestinal Malignanc*” OR “Gastrointestinal Tumor*” OR “Gastrointestinal Cancer*” OR “Upper Gastrointestinal Malignanc*” OR “Gastrointestinal Neoplasm*” OR “Cancer of Gastrointestinal Tract” OR “Gastrointestinal Tract Cancer*” OR “Gastrointestinal Stromal Tumor*” OR “Human Gastrointestinal Cancer*” OR “GISTs” OR “Esophago-Gastric tumor*” OR “Esophago-Gastric Malignanc*” OR “Esophago-Gastric cancer*” OR “Esophagogastric tumor*” OR “Esophagogastric Malignanc*” OR “Esophagogastric cancer*” OR “Esophagogastric neoplasm*” OR “gastroesophageal tumor*” OR “gastroesophageal Malignanc*” OR “gastroesophageal cancer*” OR “gastroesophageal neoplasm*” OR “Gastrointestinal Tract” OR “Advanced Gastrointestinal Cancer” OR “gastric” OR “esophageal” OR “Oesophageal” OR “Liver” OR “colorect*” OR “rectum” OR “sigmoid” OR “Anus” OR “rectum” OR “intestine” OR “colon*” OR “duodenum” OR “duodenal” OR “Jejunal” OR “ileal” OR “rectal” OR “anal” OR “jejunum” OR “pancreas” OR “Pancreatic” OR “esophagogastric” OR “esophago-gastric” OR “cholangiocarcinoma” OR “Hepatocellular” OR “biliary” OR “Hepatic” OR “gastroesophageal” OR “bile duct” OR “Intestinal Neoplasm*” OR “Intestine Neoplasm*” OR “Cancer of Intestin*” OR “Cancer of the Intestin*” OR “Intestinal Cancer*” OR “Secondary Gastrointestinal Cancer*” OR “Cancer of stomach” OR “Stomach neoplas*” OR “Stomach cancer*” OR “stomach tumor*” OR “Cancer of the stomach” OR “Gastroesophageal junction adenocarcinoma” OR “Gastroesophageal junction tumor*” OR “Gastroesophageal junction cancer*” OR “Esophagogastric junction adenocarcinoma” OR “Esophagogastric junction tumor*” OR “Esophagogastric junction cancer*” OR “Esophagogastric junction neoplasm*” OR “Esophagogastric adenocarcinoma*” OR “Esophageal cancer*” OR “oesophageal cancer*” OR “Esophageal neoplasm*” OR “oesophageal neoplasm*” OR “Esophageal tumor*” OR “oesophageal tumor*” OR “Esophageal adenoma*” OR “Esophageal adenocarcinoma*” OR "oesophageal adenocarcinoma*" OR "gastric lymphoma*" OR "stomach carcinoma" OR "gastric carcinoma" OR "gastric adenocarcinoma" OR "stomach adenocarcinoma" OR "stomach lymphoma*" OR "adenocarcinoma of the stomach" OR "adenocarcinoma of stomach" OR "adenoma of the stomach" OR "adenocarcinoma of the gastroesophageal junction" OR "adenocarcinoma of the esophagus" OR "adenocarcinoma of esophagus" OR "Gastroesophageal tumor*" OR "Gastroesophageal cancer*" OR "Gastroesophageal neoplasm*" OR "Gastroesophageal adenocarcinoma*" OR "Gastroesophageal carcinoma*" OR "Gastro-esophageal cancer*" OR "Gastro-esophageal adenocarcinoma*" OR "cancer of stomach" OR "neoplasm of stomach" OR "tumor of stomach" OR "cancer of esophagus" OR "neoplasm of esophagus" OR "tumor of esophagus" OR "gastroesophageal junction" OR "Esophageal Neoplasm*" OR "Esophagus Neoplasm*" OR "Cancer of Esophagus" OR "Cancer of the Esophagus" OR "Esophageal Squamous Cell Carcinoma*" OR "Oesophageal Squamous Cell Carcinoma" OR "Esophagus Cancer*" OR "Stomach Neoplasm*" OR "Colorectal Neoplasm*" OR "Colorectal Cancer*" OR "Colorectal Tumor*" OR "Colorectal Carcinoma*" OR "Gastric Cancer*" OR "Hepatocellular Carcinoma*" OR "Esophageal Cancer*" OR "Oesophageal Cancer*" OR "Gastroesophageal Cancer*" OR "Distal Gastric Cancer*" OR "Gastroesophageal Junction Malignanc*" OR "Gastro-Oesophageal Junction Cancer*" OR "Gastroesophageal Carcinoma*" OR "Cancer Of The Gastroesophageal Junction" OR "Esophageal Malignanc*" OR "Oesophageal Malignanc*" OR "Esophageal Neoplasia*" OR "Esophageal Neoplasm*" OR "Cancer Of The Esophagus" OR "Cancer Of The Stomach" OR "Cancer Of The Colon" OR "Cancer Of Colon" OR "Upper Gastrointestinal Cancer*" OR "Liver Cancer*" OR "Hepatocellular Carcinoma*" OR "Hepatic Cancer*" OR "Hepatic neoplasm*" OR "Primary Hepatic Cancer*" OR "Hepatic Malignanc*" OR "Malignant Liver Tumor*" OR "Liver Malignanc*" OR "Metastatic Liver Tumor*" OR "Primary And Secondary Liver Malignanc*" OR "Hepatoblastoma" OR "Hepatocellular Cancer*" OR "Cancer of Liver" OR "Percutaneous Liver Tumor*" OR "Malignant Liver Tumor*" OR "Metastatic Liver Tumor*" OR " Liver Neoplasm*" OR "Primary Liver Neoplasm*" OR "Malignant Liver Neoplasm*" OR "Liver Neoplasia*" OR "Hepatic Neoplasia*" OR "Duodenal Cancer*" OR "Cancer Of The Duodenum" OR "Small Intestine Cancer*" OR "Duodenal Tumor*" OR "Duodenal Malignanc*" OR "Stromal Tumor*" OR "Duodenal Neoplasm*" OR "Duodenal Epithelial Neoplasm*" OR "Small Bowel Cancer*" OR "Cancer Of The Jejunum" OR "Villous Tumors" OR "Jejunal Carcinoma*" OR "Jejunum Cancer" OR "Jejunal Neoplasm*" OR "Jejunal cancer*" OR "Jejunal Malignant Lymphoma" OR "Primary Jejunal Adenocarcinoma*" OR "Ileum Cancer*" OR "Tumor Of The Ileum" OR "Carcinoid Tumor Of The Ileum" OR "Stromal Tumor*" OR "Malignant Glomus Tumor*" OR "Proximal Colon Cancer*" OR "Neoplasm Of the Small Intestine" OR "Ileal Neuroendocrine Neoplasm*" OR "Ileal Lymphoma" OR "Pancreatic Neoplasm*" OR "Pancreatic Cancer*" OR "Advanced Pancreatic Cancer*" OR "Pancreatic Tumor*" OR "Pancreatic Malignanc*" OR "Hepatobiliary Pancreatic Malignanc*" OR "Pancreatic Ductal Adenocarcinoma*" OR "Pancreatic Neoplasm*" OR "Pancreas cancer*" OR "Pancreas neoplasm*" OR "Pancreas carcinoma*" OR "Pancreas neoplasia*" OR "Anal Cancer*" OR "Anal neoplasm*" OR "Anus neoplasm*" OR "Cancer of the Anus" OR "Cancer of Anus" OR "Colon Cancer*" OR "Colorectal Cancer*" OR "Rectal Cancer*" OR "Rectal Tumor*" OR "Rectal Neoplasm*" OR "Rectal Neoplasia*" OR "Rectum cancer*" OR "Cancer of the Rectum" OR "Cancer of Rectum" OR "Anus Cancer*" OR "Carcinoma Of The Colon" OR "Tumor Of The Colon" OR "Anal Malignanc*" OR "Anal Carcinoma*" OR "Malignant Colonic Obstruction" OR "Neoplasm Of The Anus" OR "Colon cancer*" OR "Colon Adenocarcinoma" OR "Intrahepatic cholangiocarcinoma*" OR "Biliary Cancer*" OR "Gallbladder Cancer*" OR "Gallbladder Neoplasm*" OR "Biliary Tract Cancer*" OR "Biliary Tract Carcinoma*" OR "Cancer of the Gallbladder" OR "Biliary Tract Malignanc*" OR "Biliary Malignanc*" OR "Bile Duct Cancer*" OR "Gallbladder Cancer*" OR "Gallbladder And Bile Duct Neoplasm*" OR "Biliary Tract Neoplasm*" OR "Biliary Neoplasm*" OR "Gallbladder Neoplasm*" OR "Neoplasm Of The Biliary Tract" OR "Neoplasm of Gallbladder" OR "Neoplasm Of The Gallbladder" OR "Tumor Of The Gallbladder" OR "Biliary Tract tumor*" OR "Biliary Intraepithelial Neoplasia*" OR "Gallbladder Neoplasia*" OR "Biliary Neoplasia*" OR "Gallbladder Carcinoma*" OR "Biliary Tract Intraepithelial Neoplasia*" OR "Small bowel cancer*" OR "Small Intestine Cancer*" OR "Cancer Of The Small Intestine" OR "Small Intestine Cancer*" OR "Small Intestinal Tumor*" OR "Adenocarcinoma Of The Small Intestine" OR "Small Intestinal Neoplasm*" OR "Lymphoma Of The Small Intestine" OR "Small Intestinal Neoplasm*" OR "Small Intestinal Neoplasm*" OR "Neoplasm Of The Small Intestine" OR "Small Intestinal Neoplasia*" OR "Colorectal Cancer" OR "Colorectal Tumor*" OR "Colorectal Malignanc*" OR "Colorectal Neoplasm*" OR "Colorectal Neoplasia*" OR "Advanced Colorectal Neoplasia*" OR "Colorectal Neoplasia" OR "Transverse Colon Cancer*" OR "Transverse Colon Cancer*" OR "Transverse Colon Carcinoma*" OR "Transverse Colon Neoplasm*" OR "Descending Colon Cancer*" OR "Ascending Colon Cancer*" OR "Descending Colon Tumor*" OR "Ascending Colon Tumor*" OR "Left Sided Colonic Tumor*" OR "Ascending Colon Cancer*" OR "Sigmoid Colon Cancer*" OR "Sigmoid Colon Tumor*" OR "Adenocarcinoma of Sigmoid Colon" OR "Rectosigmoid Adenomas" OR "Cecum Cancer*" OR "Cancer of Cecum" OR "Cecal Tumor*" OR "Cecum Tumor*" OR "Cecal Malignanc*" OR "Carcinoma Of The Cecum" OR "Cecal Carcinoma*" OR "Cecal Neoplasm*" OR "Colon And Rectum Cancer*" OR "Rectum Cancer*" OR "Cancer Of The Colon And Rectum" OR "Rectal Tumor*" OR "Rectal Malignanc*" OR "Primary Rectal Malignanc*" OR "Rectal Malignant Tumor*" OR "Rectal Neoplasm*" OR "Rectal Neoplasia*" OR "Appendiceal Neoplasm*" OR "Familial Polyposis Syndrome*" OR "Polypos*" OR "colonic neoplasm*" OR "colonic cancer*" OR "Sigmoid Neoplasm*" OR "Sigmoid Colon Neoplasm*" OR "Sigmoidal Cancer*" OR "Cancer of Sigmoid" OR "Cancer of the Sigmoid" OR "Rectal Tumor*" OR "Bile Duct Neoplasm*" OR "Gastrointestinal Neoplasms" OR "Esophageal Neoplasms" OR "Esophageal Squamous Cell Carcinoma" OR "Intestinal Neoplasms" OR "Cecal Neoplasms" OR "Appendiceal Neoplasms" OR "Colorectal Neoplasms" OR "Adenomatous Polyposis Coli" OR "Colonic Neoplasms" OR "Sigmoid Neoplasms" OR "Colorectal Neoplasms, Hereditary Nonpolyposis" OR "Rectal Neoplasms" OR "Anus Neoplasms" OR "Duodenal Neoplasms" OR "Ileal Neoplasms" OR "Jejunal Neoplasms" OR "Stomach Neoplasms" OR "Liver Neoplasms" OR "Carcinoma, Hepatocellular" OR "Adenoma, Liver Cell" OR "Liver Neoplasms, Experimental" OR "Pancreatic Neoplasms" OR "Carcinoma, Pancreatic Ductal" OR "Pancreatic Intraductal Neoplasms" OR "Gallbladder Neoplasms" OR "Bile Duct Neoplasms") | 3,193,924 |
|  | #2 | TS=(“immune Checkpoint Inhibitors" OR "Immune Checkpoint Inhibitor*" OR "Immune Checkpoint Blocker*" OR "Immune Checkpoint Blockade*" OR "Immune Checkpoint Inhibition*" OR "PD-L1 Inhibitor*" OR "PD L1 Inhibitor*" OR "PDL1 Inhibitor*" OR "PD-L1 blocker*" OR "PD L1 blocker*" OR "Programmed Death-Ligand 1 Inhibitor*" OR "Programmed Death Ligand 1 Inhibitor*" OR "anti Programmed Death-Ligand 1" OR "anti-Programmed Death-Ligand 1" OR "anti Programmed Death Ligand 1" OR "anti-Programmed Death Ligand 1" OR "CTLA-4 Inhibitor*" OR "CTLA 4 Inhibitor*" OR "CTLA4 Inhibitor*" OR "CTLA-4 blocker*" OR "CTLA 4 blocker*" OR "CTLA4 blocker*" OR "Cytotoxic T-Lymphocyte Associated Protein 4 Inhibitor*" OR "Cytotoxic T Lymphocyte-Associated Protein 4 Inhibitor*" OR "Cytotoxic T-Lymphocyte Associated antigen 4 Inhibitor*" OR "Cytotoxic T Lymphocyte-Associated antigen 4 Inhibitor*" OR "Cytotoxic T-Lymphocyte-Associated Protein 4 Inhibitor*" OR "Cytotoxic T Lymphocyte Associated Protein 4 Inhibitor*" OR "Cytotoxic T-Lymphocyte-Associated antigen 4 Inhibitor*" OR "Cytotoxic T Lymphocyte Associated antigen 4 Inhibitor*" OR "anti-cytotoxic T lymphocyte-associated antigen 4" OR "anti cytotoxic T lymphocyte-associated antigen 4" OR "anti-cytotoxic T lymphocyte-associated protein 4" OR "anti cytotoxic T lymphocyte-associated protein 4" OR "anti-cytotoxic T-lymphocyte-associated antigen 4" OR "anti-cytotoxic T-lymphocyte-associated antigen 4" OR "PD-1 Inhibitor*" OR "PD 1 Inhibitor*" OR "PD1 Inhibitor*" OR "PD 1 blocker*" OR "PD-1 blocker*" OR "Programmed Cell Death Protein 1 Inhibitor*" OR "anti Programmed Cell Death Protein 1" OR "anti-Programmed Cell Death Protein 1" OR "anti-PD1" OR "anti PD1" OR "anti-PD-1" OR "anti PD-1" OR "anti-PD-L1" OR "anti PDL1" OR "anti PD-L1" OR "anti-PDL1" OR "anti-PD L1" OR "anti PD L1" OR "anti-CTLA4" OR "anti-CTLA 4" OR "anti-CTLA-4" OR "anti CTLA4" OR "anti CTLA 4" OR "anti CTLA-4" OR "PD-1-PD-L1 Blockade*" OR "PD 1 PD L1 Blockade*" OR "Pembrolizumab" OR "MK-3475" OR "lambrolizumab" OR "Keytruda" OR "SCH-900475" OR "Nivolumab" OR "Opdivo" OR "ONO-4538" OR "ONO 4538" OR "ONO4538" OR "MDX-1106" OR "MDX 1106" OR "MDX1106" OR "BMS-936558" OR "BMS 936558" OR "BMS936558" OR "Ipilimumab" OR "Yervoy" OR "MDX 010" OR "MDX010" OR "MDX-010" OR "MDX-CTLA-4" OR "MDX CTLA 4" OR "Durvalumab" OR "MEDI4736" OR "MEDI-4736" OR "Imfinzi" OR "Dostarlimab" OR "TSR-042" OR "Cemiplimab" OR "REGN2810" OR "Avelumab" OR "MSB0010682" OR "bavencio" OR "MSB0010718C" OR "MSB-0010718C" OR "Atezolizumab" OR "MPDL3280A" OR "MPDL-3280A" OR "Tecentriq" OR "RG7446" OR "RG-7446" OR "pidilizumab" OR "CT-011" OR "CT 011" OR "tremelimumab" OR "ticilimumab" OR "CP 675" OR "CP-675" OR "CP-675,206" OR "CP-675206" OR "CP675206" OR "CP 675206" OR "dostarlimab" OR "cemiplimab" OR "Ipilimumab" OR "tremelimumab" OR "pembrolizumab" OR "atezolizumab" OR "Nivolumab" OR "durvalumab" OR "avelumab" OR "pidilizumab" OR "sintilimab" OR "camrelizumab" OR "toripalimab" OR "HX008" OR "tremelimumab" OR "PD-1" OR "PD-L1" OR "CTLA-4" OR "PD-1/PD-L1" OR "PD 1" OR "PD L1" OR "CTLA 4") | 90,599 |
|  | #3 | TS=(“meta-analysis” OR “meta analysis” OR "meta-analyses" OR "meta analyses" OR "meta-analyze" OR "metaanalysis" OR “metaanalyze” OR “Network Meta-Analysis” OR “Systematic Review” OR “pooled analysis”) | 631,748 |
|  | #4 | #1 AND #2 AND #3 | 511 |
| EMBASE  (9.1.2022) | #1 | (“Gastrointestinal Malignanc*” OR “Gastrointestinal Tumor*” OR “Gastrointestinal Cancer*” OR “Upper Gastrointestinal Malignanc*” OR “Gastrointestinal Neoplasm*” OR “Cancer of Gastrointestinal Tract” OR “Gastrointestinal Tract Cancer*” OR “Gastrointestinal Stromal Tumor*” OR “Human Gastrointestinal Cancer*” OR “GISTs” OR “Esophago-Gastric tumor*” OR “Esophago-Gastric Malignanc*” OR “Esophago-Gastric cancer*” OR “Esophagogastric tumor*” OR “Esophagogastric Malignanc*” OR “Esophagogastric cancer*” OR “Esophagogastric neoplasm*” OR “gastroesophageal tumor*” OR “gastroesophageal Malignanc*” OR “gastroesophageal cancer*” OR “gastroesophageal neoplasm*” OR “Gastrointestinal Tract” OR “Advanced Gastrointestinal Cancer” OR “gastric” OR “esophageal” OR “Oesophageal” OR “Liver” OR “colorect*” OR “rectum” OR “sigmoid” OR “Anus” OR “rectum” OR “intestine” OR “colon*” OR “duodenum” OR “duodenal” OR “Jejunal” OR “ileal” OR “rectal” OR “anal” OR “jejunum” OR “pancreas” OR “Pancreatic” OR “esophagogastric” OR “esophago-gastric” OR “cholangiocarcinoma” OR “Hepatocellular” OR “biliary” OR “Hepatic” OR “gastroesophageal” OR “bile duct” OR “Intestinal Neoplasm*” OR “Intestine Neoplasm*” OR “Cancer of Intestin*” OR “Cancer of the Intestin*” OR “Intestinal Cancer*” OR “Secondary Gastrointestinal Cancer*” OR “Cancer of stomach” OR “Stomach neoplas*” OR “Stomach cancer*” OR “stomach tumor*” OR “Cancer of the stomach” OR “Gastroesophageal junction adenocarcinoma” OR “Gastroesophageal junction tumor*” OR “Gastroesophageal junction cancer*” OR “Esophagogastric junction adenocarcinoma” OR “Esophagogastric junction tumor*” OR “Esophagogastric junction cancer*” OR “Esophagogastric junction neoplasm*” OR “Esophagogastric adenocarcinoma*” OR “Esophageal cancer*” OR “oesophageal cancer*” OR “Esophageal neoplasm*” OR “oesophageal neoplasm*” OR “Esophageal tumor*” OR “oesophageal tumor*” OR “Esophageal adenoma*” OR “Esophageal adenocarcinoma*” OR "oesophageal adenocarcinoma*" OR "gastric lymphoma*" OR "stomach carcinoma" OR "gastric carcinoma" OR "gastric adenocarcinoma" OR "stomach adenocarcinoma" OR "stomach lymphoma*" OR "adenocarcinoma of the stomach" OR "adenocarcinoma of stomach" OR "adenoma of the stomach" OR "adenocarcinoma of the gastroesophageal junction" OR "adenocarcinoma of the esophagus" OR "adenocarcinoma of esophagus" OR "Gastroesophageal tumor*" OR "Gastroesophageal cancer*" OR "Gastroesophageal neoplasm*" OR "Gastroesophageal adenocarcinoma*" OR "Gastroesophageal carcinoma*" OR "Gastro-esophageal cancer*" OR "Gastro-esophageal adenocarcinoma*" OR "cancer of stomach" OR "neoplasm of stomach" OR "tumor of stomach" OR "cancer of esophagus" OR "neoplasm of esophagus" OR "tumor of esophagus" OR "gastroesophageal junction" OR "Esophageal Neoplasm*" OR "Esophagus Neoplasm*" OR "Cancer of Esophagus" OR "Cancer of the Esophagus" OR "Esophageal Squamous Cell Carcinoma*" OR "Oesophageal Squamous Cell Carcinoma" OR "Esophagus Cancer*" OR "Stomach Neoplasm*" OR "Colorectal Neoplasm*" OR "Colorectal Cancer*" OR "Colorectal Tumor*" OR "Colorectal Carcinoma*" OR "Gastric Cancer*" OR "Hepatocellular Carcinoma*" OR "Esophageal Cancer*" OR "Oesophageal Cancer*" OR "Gastroesophageal Cancer*" OR "Distal Gastric Cancer*" OR "Gastroesophageal Junction Malignanc*" OR "Gastro-Oesophageal Junction Cancer*" OR "Gastroesophageal Carcinoma*" OR "Cancer Of The Gastroesophageal Junction" OR "Esophageal Malignanc*" OR "Oesophageal Malignanc*" OR "Esophageal Neoplasia*" OR "Esophageal Neoplasm*" OR "Cancer Of The Esophagus" OR "Cancer Of The Stomach" OR "Cancer Of The Colon" OR "Cancer Of Colon" OR "Upper Gastrointestinal Cancer*" OR "Liver Cancer*" OR "Hepatocellular Carcinoma*" OR "Hepatic Cancer*" OR "Hepatic neoplasm*" OR "Primary Hepatic Cancer*" OR "Hepatic Malignanc*" OR "Malignant Liver Tumor*" OR "Liver Malignanc*" OR "Metastatic Liver Tumor*" OR "Primary And Secondary Liver Malignanc*" OR "Hepatoblastoma" OR "Hepatocellular Cancer*" OR "Cancer of Liver" OR "Percutaneous Liver Tumor*" OR "Malignant Liver Tumor*" OR "Metastatic Liver Tumor*" OR " Liver Neoplasm*" OR "Primary Liver Neoplasm*" OR "Malignant Liver Neoplasm*" OR "Liver Neoplasia*" OR "Hepatic Neoplasia*" OR "Duodenal Cancer*" OR "Cancer Of The Duodenum" OR "Small Intestine Cancer*" OR "Duodenal Tumor*" OR "Duodenal Malignanc*" OR "Stromal Tumor*" OR "Duodenal Neoplasm*" OR "Duodenal Epithelial Neoplasm*" OR "Small Bowel Cancer*" OR "Cancer Of The Jejunum" OR "Villous Tumors" OR "Jejunal Carcinoma*" OR "Jejunum Cancer" OR "Jejunal Neoplasm*" OR "Jejunal cancer*" OR "Jejunal Malignant Lymphoma" OR "Primary Jejunal Adenocarcinoma*" OR "Ileum Cancer*" OR "Tumor Of The Ileum" OR "Carcinoid Tumor Of The Ileum" OR "Stromal Tumor*" OR "Malignant Glomus Tumor*" OR "Proximal Colon Cancer*" OR "Neoplasm Of the Small Intestine" OR "Ileal Neuroendocrine Neoplasm*" OR "Ileal Lymphoma" OR "Pancreatic Neoplasm*" OR "Pancreatic Cancer*" OR "Advanced Pancreatic Cancer*" OR "Pancreatic Tumor*" OR "Pancreatic Malignanc*" OR "Hepatobiliary Pancreatic Malignanc*" OR "Pancreatic Ductal Adenocarcinoma*" OR "Pancreatic Neoplasm*" OR "Pancreas cancer*" OR "Pancreas neoplasm*" OR "Pancreas carcinoma*" OR "Pancreas neoplasia*" OR "Anal Cancer*" OR "Anal neoplasm*" OR "Anus neoplasm*" OR "Cancer of the Anus" OR "Cancer of Anus" OR "Colon Cancer*" OR "Colorectal Cancer*" OR "Rectal Cancer*" OR "Rectal Tumor*" OR "Rectal Neoplasm*" OR "Rectal Neoplasia*" OR "Rectum cancer*" OR "Cancer of the Rectum" OR "Cancer of Rectum" OR "Anus Cancer*" OR "Carcinoma Of The Colon" OR "Tumor Of The Colon" OR "Anal Malignanc*" OR "Anal Carcinoma*" OR "Malignant Colonic Obstruction" OR "Neoplasm Of The Anus" OR "Colon cancer*" OR "Colon Adenocarcinoma" OR "Intrahepatic cholangiocarcinoma*" OR "Biliary Cancer*" OR "Gallbladder Cancer*" OR "Gallbladder Neoplasm*" OR "Biliary Tract Cancer*" OR "Biliary Tract Carcinoma*" OR "Cancer of the Gallbladder" OR "Biliary Tract Malignanc*" OR "Biliary Malignanc*" OR "Bile Duct Cancer*" OR "Gallbladder Cancer*" OR "Gallbladder And Bile Duct Neoplasm*" OR "Biliary Tract Neoplasm*" OR "Biliary Neoplasm*" OR "Gallbladder Neoplasm*" OR "Neoplasm Of The Biliary Tract" OR "Neoplasm of Gallbladder" OR "Neoplasm Of The Gallbladder" OR "Tumor Of The Gallbladder" OR "Biliary Tract tumor*" OR "Biliary Intraepithelial Neoplasia*" OR "Gallbladder Neoplasia*" OR "Biliary Neoplasia*" OR "Gallbladder Carcinoma*" OR "Biliary Tract Intraepithelial Neoplasia*" OR "Small bowel cancer*" OR "Small Intestine Cancer*" OR "Cancer Of The Small Intestine" OR "Small Intestine Cancer*" OR "Small Intestinal Tumor*" OR "Adenocarcinoma Of The Small Intestine" OR "Small Intestinal Neoplasm*" OR "Lymphoma Of The Small Intestine" OR "Small Intestinal Neoplasm*" OR "Small Intestinal Neoplasm*" OR "Neoplasm Of The Small Intestine" OR "Small Intestinal Neoplasia*" OR "Colorectal Cancer" OR "Colorectal Tumor*" OR "Colorectal Malignanc*" OR "Colorectal Neoplasm*" OR "Colorectal Neoplasia*" OR "Advanced Colorectal Neoplasia*" OR "Colorectal Neoplasia" OR "Transverse Colon Cancer*" OR "Transverse Colon Cancer*" OR "Transverse Colon Carcinoma*" OR "Transverse Colon Neoplasm*" OR "Descending Colon Cancer*" OR "Ascending Colon Cancer*" OR "Descending Colon Tumor*" OR "Ascending Colon Tumor*" OR "Left Sided Colonic Tumor*" OR "Ascending Colon Cancer*" OR "Sigmoid Colon Cancer*" OR "Sigmoid Colon Tumor*" OR "Adenocarcinoma of Sigmoid Colon" OR "Rectosigmoid Adenomas" OR "Cecum Cancer*" OR "Cancer of Cecum" OR "Cecal Tumor*" OR "Cecum Tumor*" OR "Cecal Malignanc*" OR "Carcinoma Of The Cecum" OR "Cecal Carcinoma*" OR "Cecal Neoplasm*" OR "Colon And Rectum Cancer*" OR "Rectum Cancer*" OR "Cancer Of The Colon And Rectum" OR "Rectal Tumor*" OR "Rectal Malignanc*" OR "Primary Rectal Malignanc*" OR "Rectal Malignant Tumor*" OR "Rectal Neoplasm*" OR "Rectal Neoplasia*" OR "Appendiceal Neoplasm*" OR "Familial Polyposis Syndrome*" OR "Polypos*" OR "colonic neoplasm*" OR "colonic cancer*" OR "Sigmoid Neoplasm*" OR "Sigmoid Colon Neoplasm*" OR "Sigmoidal Cancer*" OR "Cancer of Sigmoid" OR "Cancer of the Sigmoid" OR "Rectal Tumor*" OR "Bile Duct Neoplasm*" OR "Gastrointestinal Neoplasms" OR "Esophageal Neoplasms" OR "Esophageal Squamous Cell Carcinoma" OR "Intestinal Neoplasms" OR "Cecal Neoplasms" OR "Appendiceal Neoplasms" OR "Colorectal Neoplasms" OR "Adenomatous Polyposis Coli" OR "Colonic Neoplasms" OR "Sigmoid Neoplasms" OR "Colorectal Neoplasms, Hereditary Nonpolyposis" OR "Rectal Neoplasms" OR "Anus Neoplasms" OR "Duodenal Neoplasms" OR "Ileal Neoplasms" OR "Jejunal Neoplasms" OR "Stomach Neoplasms" OR "Liver Neoplasms" OR "Carcinoma, Hepatocellular" OR "Adenoma, Liver Cell" OR "Liver Neoplasms, Experimental" OR "Pancreatic Neoplasms" OR "Carcinoma, Pancreatic Ductal" OR "Pancreatic Intraductal Neoplasms" OR "Gallbladder Neoplasms" OR "Bile Duct Neoplasms"):ab,ti | 3,726,812 |
|  | #2 | (“immune Checkpoint Inhibitors" OR "Immune Checkpoint Inhibitor*" OR "Immune Checkpoint Blocker*" OR "Immune Checkpoint Blockade*" OR "Immune Checkpoint Inhibition*" OR "PD-L1 Inhibitor*" OR "PD L1 Inhibitor*" OR "PDL1 Inhibitor*" OR "PD-L1 blocker*" OR "PD L1 blocker*" OR "Programmed Death-Ligand 1 Inhibitor*" OR "Programmed Death Ligand 1 Inhibitor*" OR "anti Programmed Death-Ligand 1" OR "anti-Programmed Death-Ligand 1" OR "anti Programmed Death Ligand 1" OR "anti-Programmed Death Ligand 1" OR "CTLA-4 Inhibitor*" OR "CTLA 4 Inhibitor*" OR "CTLA4 Inhibitor*" OR "CTLA-4 blocker*" OR "CTLA 4 blocker*" OR "CTLA4 blocker*" OR "Cytotoxic T-Lymphocyte Associated Protein 4 Inhibitor*" OR "Cytotoxic T Lymphocyte-Associated Protein 4 Inhibitor*" OR "Cytotoxic T-Lymphocyte Associated antigen 4 Inhibitor*" OR "Cytotoxic T Lymphocyte-Associated antigen 4 Inhibitor*" OR "Cytotoxic T-Lymphocyte-Associated Protein 4 Inhibitor*" OR "Cytotoxic T Lymphocyte Associated Protein 4 Inhibitor*" OR "Cytotoxic T-Lymphocyte-Associated antigen 4 Inhibitor*" OR "Cytotoxic T Lymphocyte Associated antigen 4 Inhibitor*" OR "anti-cytotoxic T lymphocyte-associated antigen 4" OR "anti cytotoxic T lymphocyte-associated antigen 4" OR "anti-cytotoxic T lymphocyte-associated protein 4" OR "anti cytotoxic T lymphocyte-associated protein 4" OR "anti-cytotoxic T-lymphocyte-associated antigen 4" OR "anti-cytotoxic T-lymphocyte-associated antigen 4" OR "PD-1 Inhibitor*" OR "PD 1 Inhibitor*" OR "PD1 Inhibitor*" OR "PD 1 blocker*" OR "PD-1 blocker*" OR "Programmed Cell Death Protein 1 Inhibitor*" OR "anti Programmed Cell Death Protein 1" OR "anti-Programmed Cell Death Protein 1" OR "anti-PD1" OR "anti PD1" OR "anti-PD-1" OR "anti PD-1" OR "anti-PD-L1" OR "anti PDL1" OR "anti PD-L1" OR "anti-PDL1" OR "anti-PD L1" OR "anti PD L1" OR "anti-CTLA4" OR "anti-CTLA 4" OR "anti-CTLA-4" OR "anti CTLA4" OR "anti CTLA 4" OR "anti CTLA-4" OR "PD-1-PD-L1 Blockade*" OR "PD 1 PD L1 Blockade*" OR "Pembrolizumab" OR "MK-3475" OR "lambrolizumab" OR "Keytruda" OR "SCH-900475" OR "Nivolumab" OR "Opdivo" OR "ONO-4538" OR "ONO 4538" OR "ONO4538" OR "MDX-1106" OR "MDX 1106" OR "MDX1106" OR "BMS-936558" OR "BMS 936558" OR "BMS936558" OR "Ipilimumab" OR "Yervoy" OR "MDX 010" OR "MDX010" OR "MDX-010" OR "MDX-CTLA-4" OR "MDX CTLA 4" OR "Durvalumab" OR "MEDI4736" OR "MEDI-4736" OR "Imfinzi" OR "Dostarlimab" OR "TSR-042" OR "Cemiplimab" OR "REGN2810" OR "Avelumab" OR "MSB0010682" OR "bavencio" OR "MSB0010718C" OR "MSB-0010718C" OR "Atezolizumab" OR "MPDL3280A" OR "MPDL-3280A" OR "Tecentriq" OR "RG7446" OR "RG-7446" OR "pidilizumab" OR "CT-011" OR "CT 011" OR "tremelimumab" OR "ticilimumab" OR "CP 675" OR "CP-675" OR "CP-675,206" OR "CP-675206" OR "CP675206" OR "CP 675206" OR "dostarlimab" OR "cemiplimab" OR "Ipilimumab" OR "tremelimumab" OR "pembrolizumab" OR "atezolizumab" OR "Nivolumab" OR "durvalumab" OR "avelumab" OR "pidilizumab" OR "sintilimab" OR "camrelizumab" OR "toripalimab" OR "HX008" OR "tremelimumab" OR "PD-1" OR "PD-L1" OR "CTLA-4" OR "PD-1/PD-L1" OR "PD 1" OR "PD L1" OR "CTLA 4"):ab,ti | 108,913 |
|  | #3 | (“meta-analysis” OR “meta analysis” OR "meta-analyses" OR "meta analyses" OR "meta-analyze" OR "metaanalysis" OR “metaanalyze” OR “Network Meta-Analysis” OR “Systematic Review” OR “pooled analysis”):ab,ti | 463,903 |
|  | #4 | #1 AND #2 AND #3 | 495 |
| Cochrane library  (9.1.2022) | #1 | (“Gastrointestinal Malignanc*”:ti,ab,kw OR “Gastrointestinal Tumor*”:ti,ab,kw OR “Gastrointestinal Cancer*”:ti,ab,kw OR “Upper Gastrointestinal Malignanc*”:ti,ab,kw OR “Gastrointestinal Neoplasm*”:ti,ab,kw OR “Cancer of Gastrointestinal Tract”:ti,ab,kw OR “Gastrointestinal Tract Cancer*”:ti,ab,kw OR “Gastrointestinal Stromal Tumor*”:ti,ab,kw OR “Human Gastrointestinal Cancer*”:ti,ab,kw OR “GISTs”:ti,ab,kw OR “Esophago-Gastric tumor*”:ti,ab,kw OR “Esophago-Gastric Malignanc*”:ti,ab,kw OR “Esophago-Gastric cancer*”:ti,ab,kw OR “Esophagogastric tumor*”:ti,ab,kw OR “Esophagogastric Malignanc*”:ti,ab,kw OR “Esophagogastric cancer*”:ti,ab,kw OR “Esophagogastric neoplasm*”:ti,ab,kw OR “gastroesophageal tumor*”:ti,ab,kw OR “gastroesophageal Malignanc*”:ti,ab,kw OR “gastroesophageal cancer*”:ti,ab,kw OR “gastroesophageal neoplasm*”:ti,ab,kw OR “Gastrointestinal Tract”:ti,ab,kw OR “Advanced Gastrointestinal Cancer”:ti,ab,kw OR “gastric”:ti,ab,kw OR “esophageal”:ti,ab,kw OR “Oesophageal”:ti,ab,kw OR “Liver”:ti,ab,kw OR “colorect*”:ti,ab,kw OR “rectum”:ti,ab,kw OR “sigmoid”:ti,ab,kw OR “Anus”:ti,ab,kw OR “rectum”:ti,ab,kw OR “intestine”:ti,ab,kw OR “colon*”:ti,ab,kw OR “duodenum”:ti,ab,kw OR “duodenal”:ti,ab,kw OR “Jejunal”:ti,ab,kw OR “ileal”:ti,ab,kw OR “rectal”:ti,ab,kw OR “anal”:ti,ab,kw OR “jejunum”:ti,ab,kw OR “pancreas”:ti,ab,kw OR “Pancreatic”:ti,ab,kw OR “esophagogastric”:ti,ab,kw OR “esophago-gastric”:ti,ab,kw OR “cholangiocarcinoma”:ti,ab,kw OR “Hepatocellular”:ti,ab,kw OR “biliary”:ti,ab,kw OR “Hepatic”:ti,ab,kw OR “gastroesophageal”:ti,ab,kw OR “bile duct”:ti,ab,kw OR “Intestinal Neoplasm*”:ti,ab,kw OR “Intestine Neoplasm*”:ti,ab,kw OR “Cancer of Intestin*”:ti,ab,kw OR “Cancer of the Intestin*”:ti,ab,kw OR “Intestinal Cancer*”:ti,ab,kw OR “Secondary Gastrointestinal Cancer*”:ti,ab,kw OR “Cancer of stomach”:ti,ab,kw OR “Stomach neoplas*”:ti,ab,kw OR “Stomach cancer*”:ti,ab,kw OR “stomach tumor*”:ti,ab,kw OR “Cancer of the stomach”:ti,ab,kw OR “Gastroesophageal junction adenocarcinoma”:ti,ab,kw OR “Gastroesophageal junction tumor*”:ti,ab,kw OR “Gastroesophageal junction cancer*”:ti,ab,kw OR “Esophagogastric junction adenocarcinoma”:ti,ab,kw OR “Esophagogastric junction tumor*”:ti,ab,kw OR “Esophagogastric junction cancer*”:ti,ab,kw OR “Esophagogastric junction neoplasm*”:ti,ab,kw OR “Esophagogastric adenocarcinoma*”:ti,ab,kw OR “Esophageal cancer*”:ti,ab,kw OR “oesophageal cancer*”:ti,ab,kw OR “Esophageal neoplasm*”:ti,ab,kw OR “oesophageal neoplasm*”:ti,ab,kw OR “Esophageal tumor*”:ti,ab,kw OR “oesophageal tumor*”:ti,ab,kw OR “Esophageal adenoma*”:ti,ab,kw OR “Esophageal adenocarcinoma*”:ti,ab,kw OROR "oesophageal adenocarcinoma*":ti,ab,kw OR "gastric lymphoma*":ti,ab,kw OR "stomach carcinoma":ti,ab,kw OR "gastric carcinoma":ti,ab,kw OR "gastric adenocarcinoma":ti,ab,kw OR "stomach adenocarcinoma":ti,ab,kw OR "stomach lymphoma*":ti,ab,kw OR "adenocarcinoma of the stomach":ti,ab,kw OR "adenocarcinoma of stomach":ti,ab,kw OR "adenoma of the stomach":ti,ab,kw OR "adenocarcinoma of the gastroesophageal junction":ti,ab,kw OR "adenocarcinoma of the esophagus":ti,ab,kw OR "adenocarcinoma of esophagus":ti,ab,kw OR "Gastroesophageal tumor*":ti,ab,kw OR "Gastroesophageal cancer*":ti,ab,kw OR "Gastroesophageal neoplasm*":ti,ab,kw OR "Gastroesophageal adenocarcinoma*":ti,ab,kw OR "Gastroesophageal carcinoma*":ti,ab,kw OR "Gastro-esophageal cancer*":ti,ab,kw OR "Gastro-esophageal adenocarcinoma*":ti,ab,kw OR "cancer of stomach":ti,ab,kw OR "neoplasm of stomach":ti,ab,kw OR "tumor of stomach":ti,ab,kw OR "cancer of esophagus":ti,ab,kw OR "neoplasm of esophagus":ti,ab,kw OR "tumor of esophagus":ti,ab,kw OR "gastroesophageal junction":ti,ab,kw OR "Esophageal Neoplasm*":ti,ab,kw OR "Esophagus Neoplasm*":ti,ab,kw OR "Cancer of Esophagus":ti,ab,kw OR "Cancer of the Esophagus":ti,ab,kw OR "Esophageal Squamous Cell Carcinoma*":ti,ab,kw OR "Oesophageal Squamous Cell Carcinoma":ti,ab,kw OR "Esophagus Cancer*":ti,ab,kw OR "Stomach Neoplasm*":ti,ab,kw OR "Colorectal Neoplasm*":ti,ab,kw OR "Colorectal Cancer*":ti,ab,kw OR "Colorectal Tumor*":ti,ab,kw OR "Colorectal Carcinoma*":ti,ab,kw OR "Gastric Cancer*":ti,ab,kw OR "Hepatocellular Carcinoma*":ti,ab,kw OR "Esophageal Cancer*":ti,ab,kw OR "Oesophageal Cancer*":ti,ab,kw OR "Gastroesophageal Cancer*":ti,ab,kw OR "Distal Gastric Cancer*":ti,ab,kw OR "Gastroesophageal Junction Malignanc*":ti,ab,kw OR "Gastro-Oesophageal Junction Cancer*":ti,ab,kw OR "Gastroesophageal Carcinoma*":ti,ab,kw OR "Cancer Of The Gastroesophageal Junction":ti,ab,kw OR "Esophageal Malignanc*":ti,ab,kw OR "Oesophageal Malignanc*":ti,ab,kw OR "Esophageal Neoplasia*":ti,ab,kw OR "Esophageal Neoplasm*":ti,ab,kw OR "Cancer Of The Esophagus":ti,ab,kw OR "Cancer Of The Stomach":ti,ab,kw OR "Cancer Of The Colon":ti,ab,kw OR "Cancer Of Colon":ti,ab,kw OR "Upper Gastrointestinal Cancer*":ti,ab,kw OR "Liver Cancer*":ti,ab,kw OR "Hepatocellular Carcinoma*":ti,ab,kw OR "Hepatic Cancer*":ti,ab,kw OR "Hepatic neoplasm*":ti,ab,kw OR "Primary Hepatic Cancer*":ti,ab,kw OR "Hepatic Malignanc*":ti,ab,kw OR "Malignant Liver Tumor*":ti,ab,kw OR "Liver Malignanc*":ti,ab,kw OR "Metastatic Liver Tumor*":ti,ab,kw OR "Primary And Secondary Liver Malignanc*":ti,ab,kw OR "Hepatoblastoma":ti,ab,kw OR "Hepatocellular Cancer*":ti,ab,kw OR "Cancer of Liver":ti,ab,kw OR "Percutaneous Liver Tumor*":ti,ab,kw OR "Malignant Liver Tumor*":ti,ab,kw OR "Metastatic Liver Tumor*":ti,ab,kw OR " Liver Neoplasm*":ti,ab,kw OR "Primary Liver Neoplasm*":ti,ab,kw OR "Malignant Liver Neoplasm*":ti,ab,kw OR "Liver Neoplasia*":ti,ab,kw OR "Hepatic Neoplasia*":ti,ab,kw OR "Duodenal Cancer*":ti,ab,kw OR "Cancer Of The Duodenum":ti,ab,kw OR "Small Intestine Cancer*":ti,ab,kw OR "Duodenal Tumor*":ti,ab,kw OR "Duodenal Malignanc*":ti,ab,kw OR "Stromal Tumor*":ti,ab,kw OR "Duodenal Neoplasm*":ti,ab,kw OR "Duodenal Epithelial Neoplasm*":ti,ab,kw OR "Small Bowel Cancer*":ti,ab,kw OR "Cancer Of The Jejunum":ti,ab,kw OR "Villous Tumors":ti,ab,kw OR "Jejunal Carcinoma*":ti,ab,kw OR "Jejunum Cancer":ti,ab,kw OR "Jejunal Neoplasm*":ti,ab,kw OR "Jejunal cancer*":ti,ab,kw OR "Jejunal Malignant Lymphoma":ti,ab,kw OR "Primary Jejunal Adenocarcinoma*":ti,ab,kw OR "Ileum Cancer*":ti,ab,kw OR "Tumor Of The Ileum":ti,ab,kw OR "Carcinoid Tumor Of The Ileum":ti,ab,kw OR "Stromal Tumor*":ti,ab,kw OR "Malignant Glomus Tumor*":ti,ab,kw OR "Proximal Colon Cancer*":ti,ab,kw OR "Neoplasm Of the Small Intestine":ti,ab,kw OR "Ileal Neuroendocrine Neoplasm*":ti,ab,kw OR "Ileal Lymphoma":ti,ab,kw OR "Pancreatic Neoplasm*":ti,ab,kw OR "Pancreatic Cancer*":ti,ab,kw OR "Advanced Pancreatic Cancer*":ti,ab,kw OR "Pancreatic Tumor*":ti,ab,kw OR "Pancreatic Malignanc*":ti,ab,kw OR "Hepatobiliary Pancreatic Malignanc*":ti,ab,kw OR "Pancreatic Ductal Adenocarcinoma*":ti,ab,kw OR "Pancreatic Neoplasm*":ti,ab,kw OR "Pancreas cancer*":ti,ab,kw OR "Pancreas neoplasm*":ti,ab,kw OR "Pancreas carcinoma*":ti,ab,kw OR "Pancreas neoplasia*":ti,ab,kw OR "Anal Cancer*":ti,ab,kw OR "Anal neoplasm*":ti,ab,kw OR "Anus neoplasm*":ti,ab,kw OR "Cancer of the Anus":ti,ab,kw OR "Cancer of Anus":ti,ab,kw OR "Colon Cancer*":ti,ab,kw OR "Colorectal Cancer*":ti,ab,kw OR "Rectal Cancer*":ti,ab,kw OR "Rectal Tumor*":ti,ab,kw OR "Rectal Neoplasm*":ti,ab,kw OR "Rectal Neoplasia*":ti,ab,kw OR "Rectum cancer*":ti,ab,kw OR "Cancer of the Rectum":ti,ab,kw OR "Cancer of Rectum":ti,ab,kw OR "Anus Cancer*":ti,ab,kw OR "Carcinoma Of The Colon":ti,ab,kw OR "Tumor Of The Colon":ti,ab,kw OR "Anal Malignanc*":ti,ab,kw OR "Anal Carcinoma*":ti,ab,kw OR "Malignant Colonic Obstruction":ti,ab,kw OR "Neoplasm Of The Anus":ti,ab,kw OR "Colon cancer*":ti,ab,kw OR "Colon Adenocarcinoma":ti,ab,kw OR "Intrahepatic cholangiocarcinoma*":ti,ab,kw OR "Biliary Cancer*":ti,ab,kw OR "Gallbladder Cancer*":ti,ab,kw OR "Gallbladder Neoplasm*":ti,ab,kw OR "Biliary Tract Cancer*":ti,ab,kw OR "Biliary Tract Carcinoma*":ti,ab,kw OR "Cancer of the Gallbladder":ti,ab,kw OR "Biliary Tract Malignanc*":ti,ab,kw OR "Biliary Malignanc*":ti,ab,kw OR "Bile Duct Cancer*":ti,ab,kw OR "Gallbladder Cancer*":ti,ab,kw OR "Gallbladder And Bile Duct Neoplasm*":ti,ab,kw OR "Biliary Tract Neoplasm*":ti,ab,kw OR "Biliary Neoplasm*":ti,ab,kw OR "Gallbladder Neoplasm*":ti,ab,kw OR "Neoplasm Of The Biliary Tract":ti,ab,kw OR "Neoplasm of Gallbladder":ti,ab,kw OR "Neoplasm Of The Gallbladder":ti,ab,kw OR "Tumor Of The Gallbladder":ti,ab,kw OR "Biliary Tract tumor*":ti,ab,kw OR "Biliary Intraepithelial Neoplasia*":ti,ab,kw OR "Gallbladder Neoplasia*":ti,ab,kw OR "Biliary Neoplasia*":ti,ab,kw OR "Gallbladder Carcinoma*":ti,ab,kw OR "Biliary Tract Intraepithelial Neoplasia*":ti,ab,kw OR "Small bowel cancer*":ti,ab,kw OR "Small Intestine Cancer*":ti,ab,kw OR "Cancer Of The Small Intestine":ti,ab,kw OR "Small Intestine Cancer*":ti,ab,kw OR "Small Intestinal Tumor*":ti,ab,kw OR "Adenocarcinoma Of The Small Intestine":ti,ab,kw OR "Small Intestinal Neoplasm*":ti,ab,kw OR "Lymphoma Of The Small Intestine":ti,ab,kw OR "Small Intestinal Neoplasm*":ti,ab,kw OR "Small Intestinal Neoplasm*":ti,ab,kw OR "Neoplasm Of The Small Intestine":ti,ab,kw OR "Small Intestinal Neoplasia*":ti,ab,kw OR "Colorectal Cancer":ti,ab,kw OR "Colorectal Tumor*":ti,ab,kw OR "Colorectal Malignanc*":ti,ab,kw OR "Colorectal Neoplasm*":ti,ab,kw OR "Colorectal Neoplasia*":ti,ab,kw OR "Advanced Colorectal Neoplasia*":ti,ab,kw OR "Colorectal Neoplasia":ti,ab,kw OR "Transverse Colon Cancer*":ti,ab,kw OR "Transverse Colon Cancer*":ti,ab,kw OR "Transverse Colon Carcinoma*":ti,ab,kw OR "Transverse Colon Neoplasm*":ti,ab,kw OR "Descending Colon Cancer*":ti,ab,kw OR "Ascending Colon Cancer*":ti,ab,kw OR "Descending Colon Tumor*":ti,ab,kw OR "Ascending Colon Tumor*":ti,ab,kw OR "Left Sided Colonic Tumor*":ti,ab,kw OR "Ascending Colon Cancer*":ti,ab,kw OR "Sigmoid Colon Cancer*":ti,ab,kw OR "Sigmoid Colon Tumor*":ti,ab,kw OR "Adenocarcinoma of Sigmoid Colon":ti,ab,kw OR "Rectosigmoid Adenomas":ti,ab,kw OR "Cecum Cancer*":ti,ab,kw OR "Cancer of Cecum":ti,ab,kw OR "Cecal Tumor*":ti,ab,kw OR "Cecum Tumor*":ti,ab,kw OR "Cecal Malignanc*":ti,ab,kw OR "Carcinoma Of The Cecum":ti,ab,kw OR "Cecal Carcinoma*":ti,ab,kw OR "Cecal Neoplasm*":ti,ab,kw OR "Colon And Rectum Cancer*":ti,ab,kw OR "Rectum Cancer*":ti,ab,kw OR "Cancer Of The Colon And Rectum":ti,ab,kw OR "Rectal Tumor*":ti,ab,kw OR "Rectal Malignanc*":ti,ab,kw OR "Primary Rectal Malignanc*":ti,ab,kw OR "Rectal Malignant Tumor*":ti,ab,kw OR "Rectal Neoplasm*":ti,ab,kw OR "Rectal Neoplasia*":ti,ab,kw OR "Appendiceal Neoplasm*":ti,ab,kw OR "Familial Polyposis Syndrome*":ti,ab,kw OR "Polypos*":ti,ab,kw OR "colonic neoplasm*":ti,ab,kw OR "colonic cancer*":ti,ab,kw OR "Sigmoid Neoplasm*":ti,ab,kw OR "Sigmoid Colon Neoplasm*":ti,ab,kw OR "Sigmoidal Cancer*":ti,ab,kw OR "Cancer of Sigmoid":ti,ab,kw OR "Cancer of the Sigmoid":ti,ab,kw OR "Rectal Tumor*":ti,ab,kw) | 173,278 |
|  | #2 | ("Immune Checkpoint Inhibitor*":ti,ab,kw OR "Immune Checkpoint Blocker*":ti,ab,kw OR "Immune Checkpoint Blockade*":ti,ab,kw OR "Immune Checkpoint Inhibition*":ti,ab,kw OR "PD-L1 Inhibitor*":ti,ab,kw OR "PD L1 Inhibitor*":ti,ab,kw OR "PDL1 Inhibitor*":ti,ab,kw OR "PD-L1 blocker*":ti,ab,kw OR "PD L1 blocker*":ti,ab,kw OR "Programmed Death-Ligand 1 Inhibitor*":ti,ab,kw OR "Programmed Death Ligand 1 Inhibitor*":ti,ab,kw OR "anti Programmed Death-Ligand 1":ti,ab,kw OR "anti-Programmed Death-Ligand 1":ti,ab,kw OR "anti Programmed Death Ligand 1":ti,ab,kw OR "anti-Programmed Death Ligand 1":ti,ab,kw OR "CTLA-4 Inhibitor*":ti,ab,kw OR "CTLA 4 Inhibitor*":ti,ab,kw OR "CTLA4 Inhibitor*":ti,ab,kw OR "CTLA-4 blocker*":ti,ab,kw OR "CTLA 4 blocker*":ti,ab,kw OR "CTLA4 blocker*":ti,ab,kw OR "Cytotoxic T-Lymphocyte Associated Protein 4 Inhibitor*":ti,ab,kw OR "Cytotoxic T Lymphocyte-Associated Protein 4 Inhibitor*":ti,ab,kw OR "Cytotoxic T-Lymphocyte Associated antigen 4 Inhibitor*":ti,ab,kw OR "Cytotoxic T Lymphocyte-Associated antigen 4 Inhibitor*":ti,ab,kw OR "Cytotoxic T-Lymphocyte-Associated Protein 4 Inhibitor*":ti,ab,kw OR "Cytotoxic T Lymphocyte Associated Protein 4 Inhibitor*":ti,ab,kw OR "Cytotoxic T-Lymphocyte-Associated antigen 4 Inhibitor*":ti,ab,kw OR "Cytotoxic T Lymphocyte Associated antigen 4 Inhibitor*":ti,ab,kw OR "anti-cytotoxic T lymphocyte-associated antigen 4":ti,ab,kw OR "anti cytotoxic T lymphocyte-associated antigen 4":ti,ab,kw OR "anti-cytotoxic T lymphocyte-associated protein 4":ti,ab,kw OR "anti cytotoxic T lymphocyte-associated protein 4":ti,ab,kw OR "anti-cytotoxic T-lymphocyte-associated antigen 4":ti,ab,kw OR "anti-cytotoxic T-lymphocyte-associated antigen 4":ti,ab,kw OR "PD-1 Inhibitor*":ti,ab,kw OR "PD 1 Inhibitor*":ti,ab,kw OR "PD1 Inhibitor*":ti,ab,kw OR "PD 1 blocker*":ti,ab,kw OR "PD-1 blocker*":ti,ab,kw OR "Programmed Cell Death Protein 1 Inhibitor*":ti,ab,kw OR "anti Programmed Cell Death Protein 1":ti,ab,kw OR "anti-Programmed Cell Death Protein 1":ti,ab,kw OR "anti-PD1":ti,ab,kw OR "anti PD1":ti,ab,kw OR "anti-PD-1":ti,ab,kw OR "anti PD-1":ti,ab,kw OR "anti-PD-L1":ti,ab,kw OR "anti PDL1":ti,ab,kw OR "anti PD-L1":ti,ab,kw OR "anti-PDL1":ti,ab,kw OR "anti-PD L1":ti,ab,kw OR "anti PD L1":ti,ab,kw OR "anti-CTLA4":ti,ab,kw OR "anti-CTLA 4":ti,ab,kw OR "anti-CTLA-4":ti,ab,kw OR "anti CTLA4":ti,ab,kw OR "anti CTLA 4":ti,ab,kw OR "anti CTLA-4":ti,ab,kw OR "PD-1-PD-L1 Blockade*":ti,ab,kw OR "PD 1 PD L1 Blockade*":ti,ab,kw OR "Pembrolizumab":ti,ab,kw OR "MK-3475":ti,ab,kw OR "lambrolizumab":ti,ab,kw OR "Keytruda":ti,ab,kw OR "SCH-900475":ti,ab,kw OR "Nivolumab":ti,ab,kw OR "Opdivo":ti,ab,kw OR "ONO-4538":ti,ab,kw OR "ONO 4538":ti,ab,kw OR "ONO4538":ti,ab,kw OR "MDX-1106":ti,ab,kw OR "MDX 1106":ti,ab,kw OR "MDX1106":ti,ab,kw OR "BMS-936558":ti,ab,kw OR "BMS 936558":ti,ab,kw OR "BMS936558":ti,ab,kw OR "Ipilimumab":ti,ab,kw OR "Yervoy":ti,ab,kw OR "MDX 010":ti,ab,kw OR "MDX010":ti,ab,kw OR "MDX-010":ti,ab,kw OR "MDX-CTLA-4":ti,ab,kw OR "MDX CTLA 4":ti,ab,kw OR "Durvalumab":ti,ab,kw OR "MEDI4736":ti,ab,kw OR "MEDI-4736":ti,ab,kw OR "Imfinzi":ti,ab,kw OR "Dostarlimab":ti,ab,kw OR "TSR-042":ti,ab,kw OR "Cemiplimab":ti,ab,kw OR "REGN2810":ti,ab,kw OR "Avelumab":ti,ab,kw OR "MSB0010682":ti,ab,kw OR "bavencio":ti,ab,kw OR "MSB0010718C":ti,ab,kw OR "MSB-0010718C":ti,ab,kw OR "Atezolizumab":ti,ab,kw OR "MPDL3280A":ti,ab,kw OR "MPDL-3280A":ti,ab,kw OR "Tecentriq":ti,ab,kw OR "RG7446":ti,ab,kw OR "RG-7446":ti,ab,kw OR "pidilizumab":ti,ab,kw OR "CT-011":ti,ab,kw OR "CT 011":ti,ab,kw OR "tremelimumab":ti,ab,kw OR "ticilimumab":ti,ab,kw OR "CP 675":ti,ab,kw OR "CP-675":ti,ab,kw OR "CP-675,206":ti,ab,kw OR "CP-675206":ti,ab,kw OR "CP675206":ti,ab,kw OR "CP 675206":ti,ab,kw OR "dostarlimab":ti,ab,kw OR "cemiplimab":ti,ab,kw OR "Ipilimumab":ti,ab,kw OR "tremelimumab":ti,ab,kw OR "pembrolizumab":ti,ab,kw OR "atezolizumab":ti,ab,kw OR "Nivolumab":ti,ab,kw OR "durvalumab":ti,ab,kw OR "avelumab":ti,ab,kw OR "pidilizumab":ti,ab,kw OR "sintilimab":ti,ab,kw OR "camrelizumab":ti,ab,kw OR "toripalimab":ti,ab,kw OR "HX008":ti,ab,kw OR "tremelimumab":ti,ab,kw OR "PD-1":ti,ab,kw OR "PD-L1":ti,ab,kw OR "CTLA-4":ti,ab,kw OR "PD-1/PD-L1":ti,ab,kw OR "PD 1":ti,ab,kw OR "PD L1":ti,ab,kw OR "CTLA 4":ti,ab,kw) | 8,886 |
|  | #3 | (“meta-analysis”:ti,ab,kw OR “meta analysis”:ti,ab,kw OR "meta-analyses":ti,ab,kw OR "meta analyses":ti,ab,kw OR "meta-analyze":ti,ab,kw OR "meta analyze":ti,ab,kw OR "metaanalysis":ti,ab,kw OR “metaanalyze”:ti,ab,kw OR “Network Meta-Analysis”:ti,ab,kw OR “Systematic Review”:ti,ab,kw OR “pooled analysis”:ti,ab,kw) | 28,770 |
|  | #4 | #1 AND #2 AND #3 | 2 |

**Table S2.** Definition of efficacy and safety outcomes

| Measure | Definition |
| --- | --- |
| PFS | The length of time during and after the treatment of a disease, such as cancer, that a patient lives with the disease but it does not get worse. |
| OS | The length of time from either the date of diagnosis or the start of treatment for a disease, such as cancer, that patients diagnosed with the disease are still alive. |
| ORR | The percentage of people in a study or treatment group who have a partial response or complete response to the treatment within a certain period of time. |
| DCR | The percentage of patients with advanced or metastatic cancer who have achieved complete response, partial response to the treatment within a certain period of time. |
| CR | The disappearance of all signs of cancer in response to treatment. |
| PR | A decrease in the size of a tumor, or in the extent of cancer in the body, in response to treatment. |
| SD | Cancer that is neither decreasing nor increasing in extent or severity. |
| PD | Cancer that is growing, spreading, or getting worse. |
| TRAE | Treatment-related AEs refers to the causality assessment adverse events by the investigator. |
| Serious AE | Serious AEs include adverse events that result in death, require either inpatient hospitalization or the prolongation of hospitalization, are life-threatening, result in a persistent or significant disability/incapacity or result in a congenital anomaly/birth defect. |

**Abbreviations:** OS: Overall survival; PFS: Progression-free survival; ORR: Objective response rate; DCR: Disease control rate; CR: Complete response; PR: Partial response; SD: Stable disease; SD: Stable disease; PD: Progressive disease; AEs: Adverse events.

**Table S3.** Characteristics of included studies.

| Author | Cancer type | Title of included RCTs | NCT identifier of RCTs | Efficacy outcomes (effect size) | Subgroup analysis of efficacy outcomes | Safety outcomes (effect size) |
| --- | --- | --- | --- | --- | --- | --- |
| Zeng, 2022 (1) | Hepatocellular carcinoma | KEYNOTE-240 | NCT02702401 | OS (HR) PFS (HR)  DCR (RR)  ORR (RR) | Sex  Age Region Barcelona Clinic Liver Cancer status ECOG PS Extrahepatic spread Viral status | AEs ≥grade 3 AEs |
|  | Hepatocellular carcinoma | CheckMate 459 | NCT02576509 |  |  |  |
|  | Hepatocellular carcinoma | IMbrave150 | NCT03434379 |  |  |  |
| Chen, 2021 (2) | Gastric cancer | JAVELIN Gastric 300 | NCT02625623 | OS (HR) PFS (HR)  ORR (OR) | PD-L1 expression | Any AEs (OR) ≥grade 3 AEs |
|  | Gastric cancer | KEYNOTE-061 | NCT02370498 |  |  |  |
|  | Gastric cancer | ATTRACTION-2 | NCT02267343 |  |  |  |
|  | Esophageal cancer | KEYNOTE-181 | NCT02564263 |  |  |  |
| Formica, 2021 (3) | Gastric cancer | ATTRACTION-2 | NCT02267343 | OS (HR) | Region Tumor site Age Sex ECOG PS Histology PDL1 expression | NA |
|  | Gastric cancer | KEYNOTE-061 | NCT02370498 |  |  |  |
|  | Gastric cancer | KEYNOTE-062 | NCT02494583 |  |  |  |
|  | Gastric cancer | JAVELIN Gastric 300 | NCT02625623 |  |  |  |
| Kamposioras, 2021 (4) | Gastric cancer | KEYNOTE-062 | NCT02494583 | OS (HR) PFS (HR) ORR (OR) | PD-L1 expression | NA |
|  | Gastric cancer | ATTRACTION-4 | NCT02746796 |  |  |  |
|  | Gastric cancer | CheckMate 649 | NCT02872116 |  |  |  |
|  | Esophageal cancer | KEYNOTE-590 | NCT03189719 |  |  |  |
|  | Esophageal cancer | CheckMate 648 | NCT03143153 |  |  |  |
|  | Esophageal cancer | ESCORT-1st | NCT03691090 |  |  |  |
|  | Gastric cancer | JAVELIN Gastric 100 | NCT02625610 |  |  |  |
|  | Gastric cancer | Bang et al. | NCT01585987 |  |  |  |
|  | Gastric cancer | PLATFORM | NCT02678182 |  |  |  |
|  | Esophageal cancer | ESCORT | NCT03099382 |  |  |  |
|  | Esophageal cancer | ATTRACTION-3 | NCT02569242 |  |  |  |
|  | Esophageal cancer | KEYNOTE-181 | NCT02564263 |  |  |  |
|  | Gastric cancer | KEYNOTE-061 | NCT02370498 |  |  |  |
|  | Gastric cancer | KEYNOTE-063 trial | NCT02370498 |  |  |  |
|  | Esophageal cancer | RATIONALE 302 | NCT03430843 |  |  |  |
| Maoxi, 2021 (5) | Gastric cancer | JAVELIN Gastric 300 | NCT02625623 | OS (HR) PFS (HR) ORR (RR) | Histology Tumor site Line of therapy PD-L1 expression ECOG PS Sex  Age Region | Any TRAEs (OR) ≥grade 3 TRAEs |
|  | Gastric cancer | KEYNOTE-061 | NCT02370498 |  |  |  |
|  | Esophageal cancer | KEYNOTE-181 | NCT02564263 |  |  |  |
|  | Esophageal cancer | ATTRACTION-3 | NCT02569242 |  |  |  |
|  | Esophageal cancer | ESCORT | NCT03099382 |  |  |  |
|  | Esophageal cancer | ORIENT-2 | NCT03116152 |  |  |  |
| Oh, 2021 (6) | Gastric cancer | ATTRACTION-2 | NCT02267343 | OS (HR) PFS (HR) | Target Drug type  Line of therapy Tumor site | Serious AEs (OR) |
|  | Gastric cancer | KEYNOTE-061 | NCT02370498 |  |  |  |
|  | Gastric cancer | JAVELIN Gastric 300 | NCT02625623 |  |  |  |
|  | Esophageal cancer | ATTRACTION-3 | NCT02569242 |  |  |  |
|  | Esophageal cancer | KEYNOTE-590 | NCT03189719 |  |  |  |
|  | Esophageal cancer | KEYNOTE-181 | NCT02564263 |  |  |  |
|  | Esophageal cancer | ESCORT | NCT03099382 |  |  |  |
|  | Gastric cancer | KEYNOTE-062 | NCT02494583 |  |  |  |
| Pietrantonio, 2021 (7) | Gastric cancer | KEYNOTE-062 | NCT02494583 | OS (HR) PFS (HR) ORR (RR) | Microsatellite instability status | NA |
|  | Gastric cancer | CheckMate 649 | NCT02872116 |  |  |  |
|  | Gastric cancer | JAVELIN Gastric 100 | NCT02625610 |  |  |  |
|  | Gastric cancer | KEYNOTE-061 | NCT02370498 |  |  |  |
| Xie, 2021 (8) | Gastric cancer | KEYNOTE-062 | NCT02494583 | OS (HR) PFS (HR) | PD-L1 expression | NA |
|  | Gastric cancer | KEYNOTE-061 | NCT02370498 |  |  |  |
|  | Gastric cancer | ATTRACTION-2 | NCT02267343 |  |  |  |
|  | Gastric cancer | CheckMate 649 | NCT02872116 |  |  |  |
|  | Gastric cancer | JAVELIN Gastric 300 | NCT02625623 |  |  |  |
|  | Gastric cancer | JAVELIN Gastric 100 | [NCT02625610](http://clinicaltrials.gov/show/NCT02625610) |  |  |  |
| Gu, 2021 (9) | Esophageal cancer | KEYNOTE-181 | NCT02564263 | OS (HR) PFS (HR) ORR (RR) DCR (RR) | PD-L1 expression | TRAEs (RR) |
|  | Esophageal cancer | ESCORT | NCT03099382 |  |  |  |
|  | Esophageal cancer | ATTRACTION-3 | NCT02569242 |  |  |  |
| Leone, 2022 (10) | Esophageal cancer | KEYNOTE-590 | NCT03189719 | OS (HR) PFS (HR) ORR (RR) | PD-L1 expression | NA |
|  | Esophageal cancer | KEYNOTE-181 | NCT02564263 |  |  |  |
|  | Esophageal cancer | CheckMate 648 | NCT03143153 |  |  |  |
|  | Esophageal cancer | ORIENT-15 | NCT03748134 |  |  |  |
|  | Esophageal cancer | JUPITER-06 | NCT03829969 |  |  |  |
|  | Esophageal cancer | ESCORT | NCT03099382 |  |  |  |
|  | Esophageal cancer | RATIONALE 302 | NCT03430843 |  |  |  |
|  | Esophageal cancer | ESCORT-1st | NCT03691090 |  |  |  |
|  | Esophageal cancer | ORIENT-2 | NCT03116152 |  |  |  |
|  | Esophageal cancer | ATTRACTION-3 | NCT02569242 |  |  |  |
| Lu, 2022 (11) | Esophageal cancer | CheckMate 648 | NCT03143153 | PFS (HR) OS (HR) ORR (OR) | Histology PD-L1 expression Age Sex ECOG PS | TRAEs (OR) ≥grade 3 TRAEs |
|  | Esophageal cancer | KEYNOTE-590 | NCT03189719 |  |  |  |
|  | Esophageal cancer | ESCORT-1st | NCT03691090 |  |  |  |
|  | Esophageal cancer | ORIENT-15 | NCT03748134 |  |  |  |
|  | Esophageal cancer | JUPITER-06 | NCT03829969 |  |  |  |
|  | Gastric cancer | CheckMate 649 | NCT02872116 |  |  |  |
| Zhu, 2021 (12) | Esophageal cancer | KEYNOTE-181 | NCT02564263 | OS (HR) PFS (HR) ORR (RR) DCR (RR) | PDL1 expression | TRAEs (RR) ≥grade 3 TRAEs |
|  | Esophageal cancer | ATTRACTION-3 | NCT02569242 |  |  |  |
|  | Esophageal cancer | ESCORT | NCT03099382 |  |  |  |
|  | Esophageal cancer | ORIENT-2 | NCT03116152 |  |  |  |
|  | Esophageal cancer | RATIONALE 302 | NCT03430843 |  |  |  |
| Rotundo, 2022 (13) | Colorectal cancer | BACCI | NCT02873195 | PFS (HR) OS (HR) ORR | NA | AEs ≥grade 3 AEs |
|  | Colorectal cancer | KEYNOTE-177 | NCT02563002 |  |  |  |
|  | Colorectal cancer | CO.26 | NCT02870920 |  |  |  |
|  | Colorectal cancer | Cohort 2 of MODUL | NCT02291289 |  |  |  |
|  | Colorectal cancer | IMblaze370 | NCT02788279 |  |  |  |
| Zeng, 2022 (14) | Colorectal cancer | IMblaze370 | NCT02788279 | OS (HR) PFS (HR) ORR (OR) DCR (OR) | NA | TRAEs ≥grade 3 TRAEs |
|  | Colorectal cancer | CO.26 | NCT02870920 |  |  |  |
|  | Colorectal cancer | KEYNOTE-177 | NCT02563002 |  |  |  |

**Abbreviations**: OS: Overall survival; PFS: Progression-free survival; ORR: Objective response rate; DCR: Disease control rate; ECOG PS: Eastern cooperative oncology group performance status; PD-L1: Programmed-death ligand 1; HR: Hazard ratio; RR: Risk ratio; OR: Odds ratio; AEs: Adverse events; TRAEs: Treatment-related adverse events; NA: Not available.

**Table S4.** Methodological quality assessment by AMSTAR2

|  | Systematic reviews | Item 1 | Item 2 | Item 3 | Item 4 | Item 5 | Item  6 | Item  7 | Item  8 | Item  9 | Item  10 | Item 11 | Item 12 | Item  13 | Item  14 | Item  15 | Item  16 | In Total of Yes  (%) | Overall quality |
| --- | --- | --- | --- | --- | --- | --- | --- | --- | --- | --- | --- | --- | --- | --- | --- | --- | --- | --- | --- |
| 1 | Zeng, 2022 (1) | Y | Y | N | N | Y | Y | N | PY | Y | N | Y | N | N | Y | Y | Y | 9 | Critically low |
| 2 | Chen, 2021 (2) | Y | N | Y | PY | N | Y | N | Y | N | N | Y | N | N | Y | Y | Y | 8 | Critically low |
| 3 | Formica, 2021 (3) | Y | N | Y | PY | Y | Y | N | PY | N | N | Y | N | N | N | N | Y | 6 | Critically low |
| 4 | Kamposioras, 2021 (4) | Y | N | Y | PY | Y | N | N | PY | N | N | Y | N | N | Y | N | Y | 6 | Critically low |
| 5 | Maoxi, 2021 (5) | Y | PY | Y | PY | Y | Y | N | Y | Y | N | Y | N | N | Y | Y | Y | 10 | Critically low |
| 6 | Oh, 2021 (6) | Y | PY | Y | PY | Y | Y | N | Y | Y | N | Y | N | N | N | Y | Y | 9 | Critically low |
| 7 | Pietrantonio, 2021 (7) | Y | N | Y | PY | Y | N | N | PY | N | N | Y | N | N | N | Y | Y | 6 | Critically low |
| 8 | Xie, 2021 (8) | Y | PY | Y | PY | Y | N | N | PY | Y | N | Y | N | N | Y | N | Y | 6 | Critically low |
| 9 | Gu, 2021 (9) | Y | PY | Y | PY | Y | Y | N | PY | Y | N | Y | N | N | Y | Y | Y | 9 | Critically low |
| 10 | Leone, 2022 (10) | Y | PY | Y | PY | N | Y | N | PY | Y | N | Y | Y | N | N | Y | Y | 8 | Critically low |
| 11 | Lu, 2022 (11) | Y | PY | Y | PY | Y | Y | N | Y | Y | N | Y | Y | N | N | N | Y | 9 | Critically low |
| 12 | Zhu, 2021 (12) | Y | N | Y | PY | N | Y | N | Y | Y | N | Y | Y | N | Y | Y | Y | 10 | Critically low |
| 13 | Rotundo, 2022 (13) | Y | N | N | PY | Y | N | N | PY | N | N | Y | N | N | Y | Y | Y | 6 | Critically low |
| 14 | Zeng, 2022 (14) | Y | Y | N | PY | N | Y | N | PY | Y | N | Y | Y | N | N | N | Y | 7 | Critically low |
|  | In Total of “Y”  (%) | 14 | 1 | 11 | 0 | 10 | 10 | 0 | 5 | 9 | 0 | 14 | 4 | 0 | 8 | 9 | 14 |  |  |

**Abbreviations**: N: No; Y: Yes; PY: Partial Yes

Item 1: Did the research questions and inclusion criteria for the review include the components of PICO?

Item 2: Did the report of the review contain an explicit statement that the review methods were established prior to the conduct of the review and did the report justify any significant deviations from the protocol?

Item 3: Did the review authors explain their selection of the study designs for inclusion in the review?

Item 4: Did the review authors use a comprehensive literature search strategy?

Item 5: Did the review authors perform study selection in duplicate?

Item 6: Did the review authors perform data extraction in duplicate?

Item 7: Did the review authors provide a list of excluded studies and justify the exclusions?

Item 8: Did the review authors describe the included studies in adequate detail?

Item 9: Did the review authors use a satisfactory technique for assessing the risk of bias?

Item 10: Did the review authors report on the sources of funding?

Item 11: Did the review authors use appropriate methods for statistical combination of results?

Item 12: Did the review authors assess the potential impact of RoB in individual studies on the results?

Item 13: Did the review authors account for RoB in individual studies when interpreting/ discussing the results of the review?

Item 14: Did the review authors provide a satisfactory explanation for, and discussion of, any heterogeneity?

Item 15: Did the review authors carry out an adequate investigation of publication bias?

Item 16: Did the review authors report any potential sources of conflict of interest?


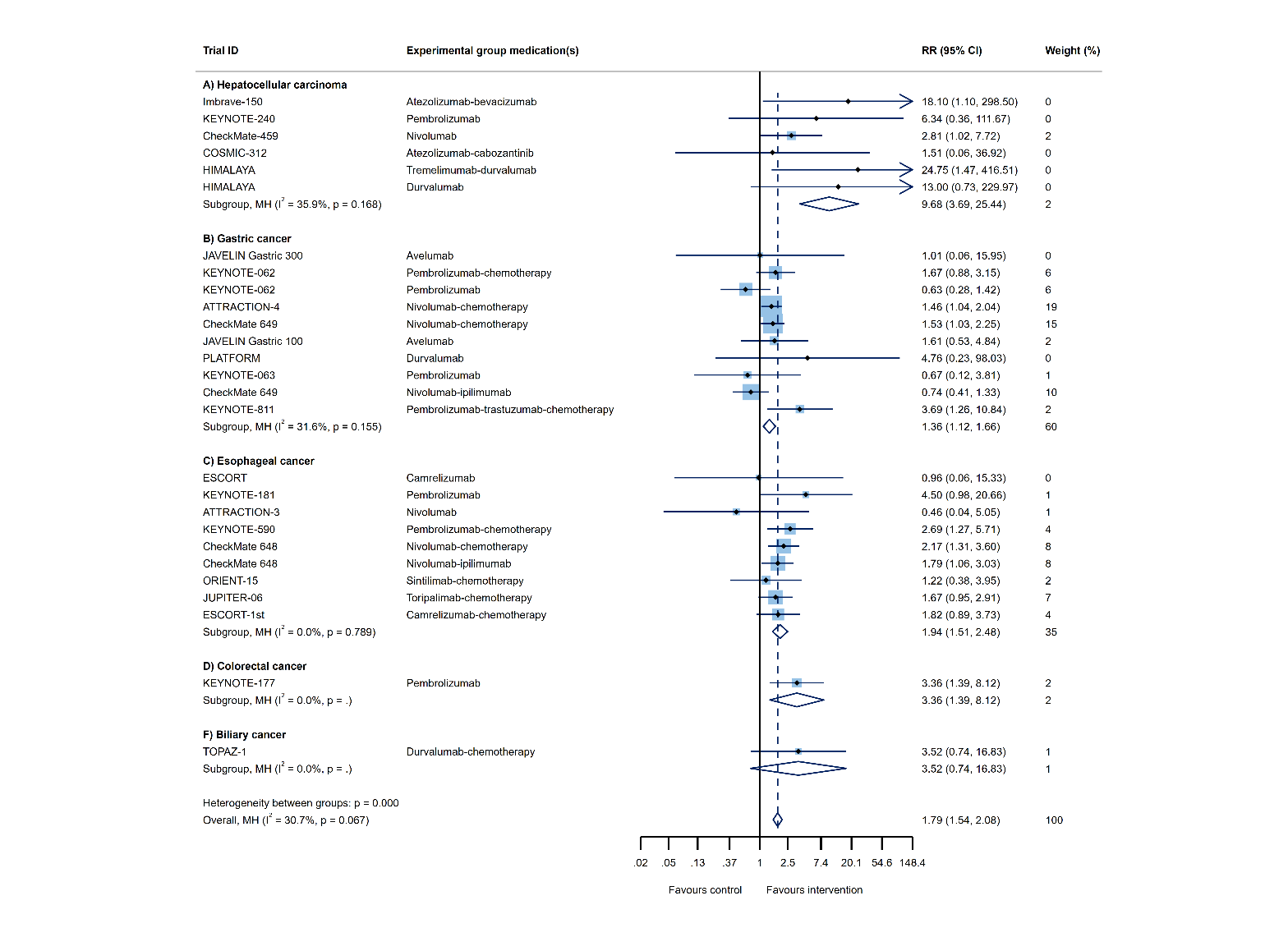


**Figure S1.** Forest plots of CR analysis in different types of GI cancers.


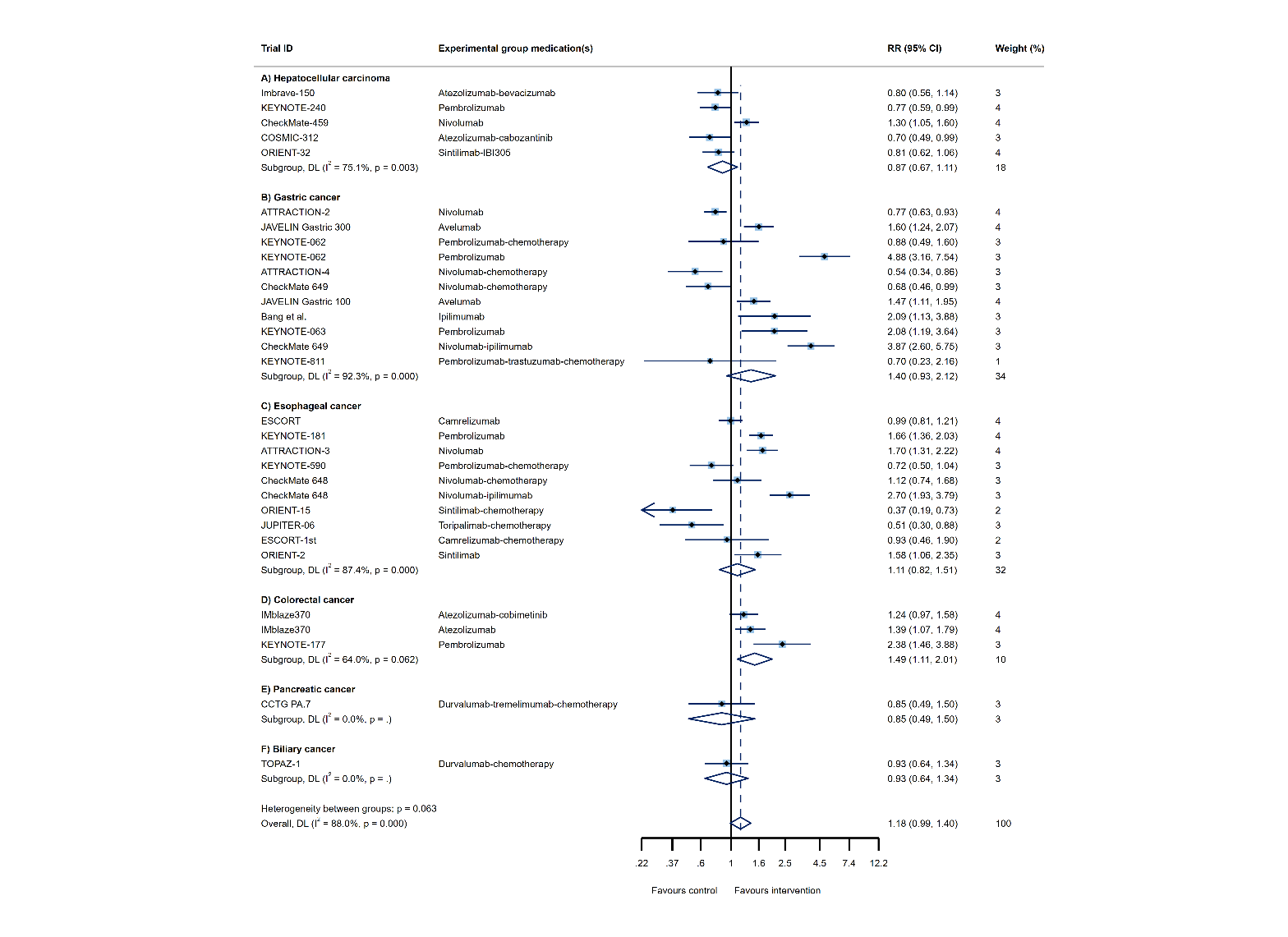


**Figure S2.** Forest plots of PD analysis in different types of GI cancers.


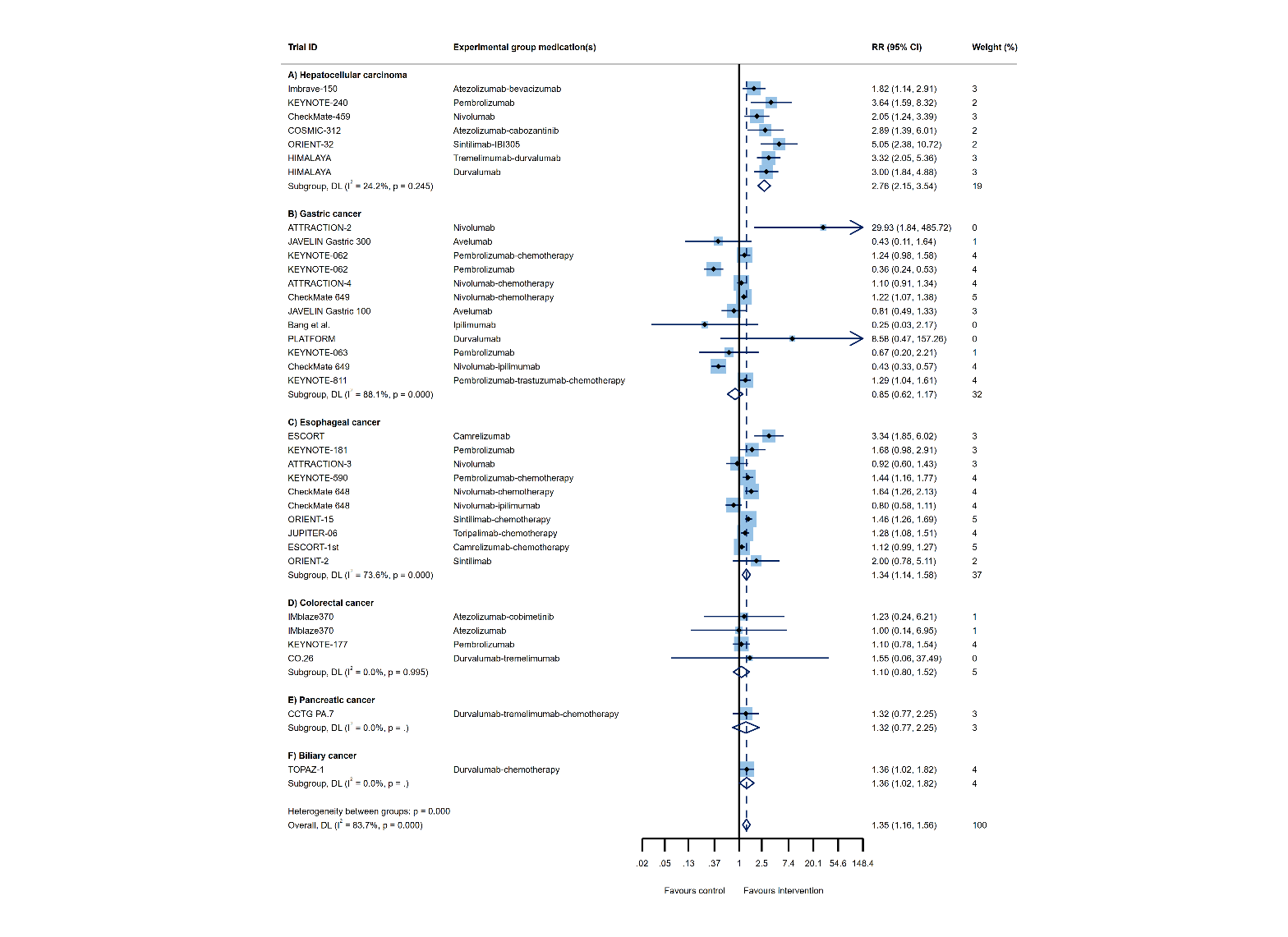


**Figure S3.** Forest plots of PR analysis in different types of GI cancers.


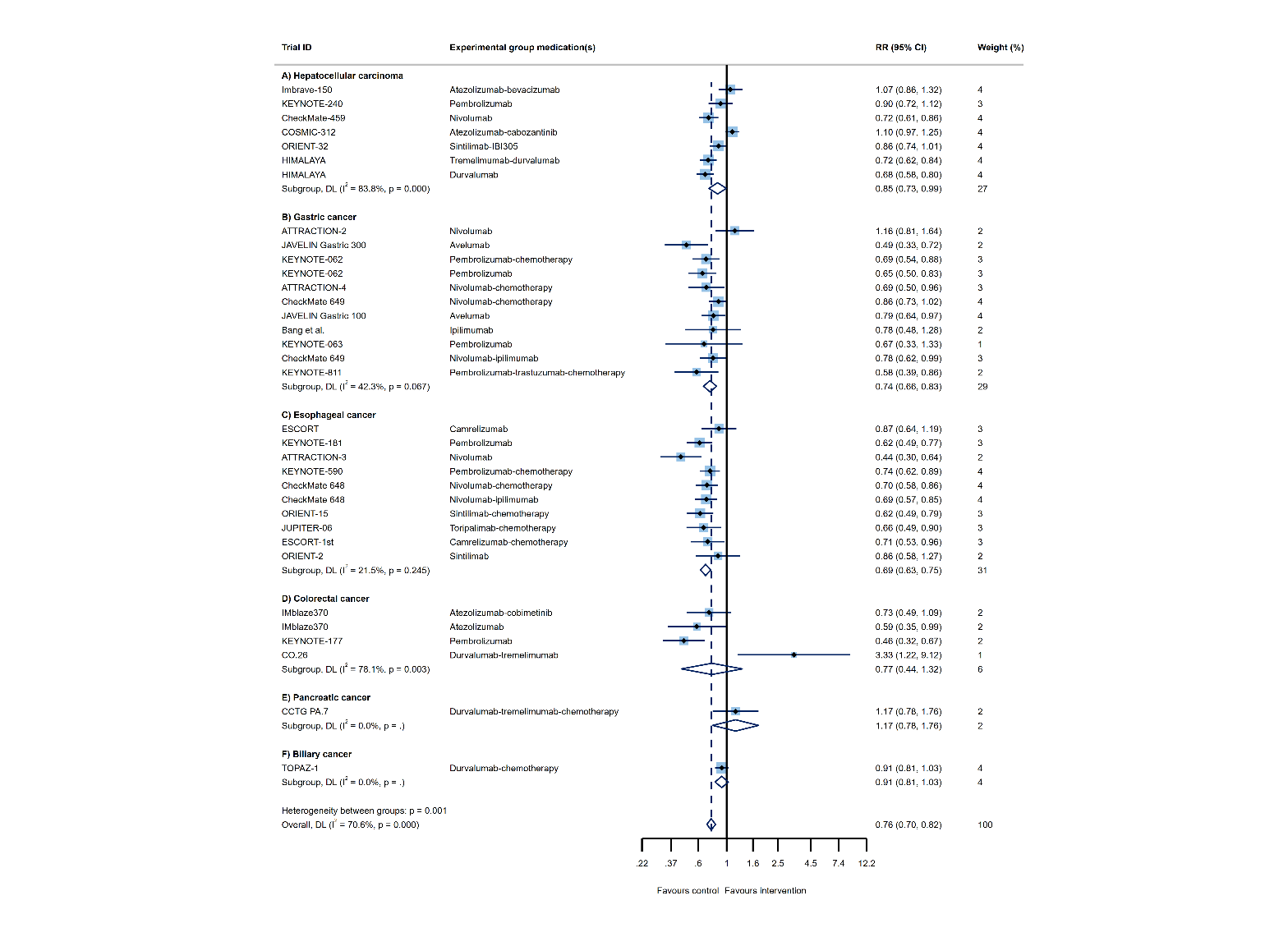


**Figure S4.** Forest plots of SD analysis in different types of GI cancers.

**References**

1. Zeng L, Su J, Qiu W, Jin X, Qiu Y, Yu W. Survival Outcomes and Safety of Programmed Cell Death/Programmed Cell Death Ligand 1 Inhibitors for Unresectable Hepatocellular Carcinoma: Result From Phase III Trials. Cancer Control. 2022;29:10732748221092924.

2. Chen K, Wang X, Yang L, Chen Z. The anti-PD-1/PD-L1 immunotherapy for gastric esophageal cancer: a systematic review and meta-analysis and literature review. Cancer Control. 2021;28:1073274821997430.

3. Formica V, Morelli C, Patrikidou A, Shiu K, Nardecchia A, Lucchetti J, et al. A systematic review and meta-analysis of PD-1/PD-L1 inhibitors in specific patient subgroups with advanced gastro-oesophageal junction and gastric adenocarcinoma. Critical Reviews in Oncology/Hematology. 2021;157:103173.

4. Kamposioras K, Ntellas P, Nikolaou M, Germetaki T, Gazouli I, Dadouli K, et al. Immunotherapy Efficacy in the Initial Lines of Treatment in Advanced Upper Gastrointestinal Malignancies: A Systematic Review of the Literature. JNCI cancer spectrum. 2021;5(6):pkab088.

5. Maoxi Z, Jinmin X, Xiaozhu Z, Yubing Y, Yuxi Z. PD-1/PD-L1 inhibitors versus chemotherapy for previously treated advanced gastroesophageal cancer: A meta-analysis of randomized controlled trials. Journal of oncology. 2021;2021.

6. Oh S, Kim E, Lee H. Comparative impact of PD-1 and PD-L1 inhibitors on advanced esophageal or gastric/gastroesophageal junction cancer treatment: a systematic review and meta-analysis. Journal of clinical medicine. 2021;10(16):3612.

7. Pietrantonio F, Randon G, Di Bartolomeo M, Luciani A, Chao J, Smyth E, et al. Predictive role of microsatellite instability for PD-1 blockade in patients with advanced gastric cancer: a meta-analysis of randomized clinical trials. ESMO open. 2021;6(1):100036.

8. Xie T, Zhang Z, Zhang X, Qi C, Shen L, Peng Z. Appropriate PD-L1 cutoff value for gastric cancer immunotherapy: a systematic review and meta-analysis. Frontiers in oncology. 2021;11:646355.

9. Gu Y-M, Shang Q-X, Zhuo Y, Zhou J-F, Liu B-W, Wang W-P, et al. Efficacy and Safety of Immune Checkpoint Inhibitor in Advanced Esophageal Squamous Cell Carcinoma: A Meta-Analysis. Frontiers in Oncology. 2021:5496.

10. Leone A, Petrelli F, Ghidini A, Raimondi A, Smyth E, Pietrantonio F. Efficacy and activity of PD-1 blockade in patients with advanced esophageal squamous cell carcinoma: a systematic review and meta-analysis with focus on the value of PD-L1 combined positive score. ESMO open. 2022;7(1):100380.

11. Lu Y, Xu M, Guan L, Yang Y, Chen Y, Yang Y, et al. PD-1 Inhibitor Plus Chemotherapy Versus Chemotherapy as First-line Treatment for Advanced Esophageal Cancer: A Systematic Review and Meta-Analysis. Journal of Immunotherapy. 2022;45(5):243-53.

12. Zhu X, Shanzhou Q, Li D, Pang X, Ma D. PD-1 inhibitors versus chemotherapy as second-line treatment for advanced esophageal squamous cell carcinoma: a meta-analysis. BMC cancer. 2021;21(1):1-10.

13. Rotundo MS, Bagnardi V, Rotundo M, Comandè M, Zampino MG. PD‑1/PD‑L1 blockade, a novel strategy for targeting metastatic colorectal cancer: A systematic review and meta‑analysis of randomized trials. Oncology Letters. 2022;23(4):1-14.

14. Zeng T, Fang X, Lu J, Zhong Y, Lin X, Lin Z, et al. Efficacy and safety of immune checkpoint inhibitors in colorectal cancer: A systematic review and meta-analysis. International Journal of Colorectal Disease. 2021:1-8.
